# Cotadutide promotes glycogenolysis in people with overweight or obesity diagnosed with type 2 diabetes

**DOI:** 10.1038/s42255-023-00938-0

**Published:** 2023-12-08

**Authors:** Victoria E. R. Parker, Darren Robertson, Edmundo Erazo-Tapia, Bas Havekes, Esther Phielix, Marlies de Ligt, Kay H. M. Roumans, Julian Mevenkamp, Folke Sjoberg, Vera B. Schrauwen-Hinderling, Edvin Johansson, Yi-Ting Chang, Russell Esterline, Kenneth Smith, Daniel J. Wilkinson, Lars Hansen, Lars Johansson, Philip Ambery, Lutz Jermutus, Patrick Schrauwen

**Affiliations:** 1 Research and Early Development, Cardiovascular, Renal and Metabolism, BioPharmaceuticals R&D, AstraZeneca, Cambridge, UK; 2 Department of Nutrition and Movement Sciences, NUTRIM School for Nutrition and Translational Research in Metabolism, Maastricht University Medical Centre, Maastricht, the Netherlands; 3 Division of Endocrinology and Metabolism, Department of Internal Medicine, Maastricht University Medical Center, Maastricht, the Netherlands; 4 Department of Radiology and Nuclear Medicine, Maastricht University Medical Center, Maastricht, the Netherlands; 5 Clinical Trial Consultants AB, Uppsala, Sweden; 6 Linköping University, Linköping, Sweden; 7 Antaros Medical AB, Mölndal, Sweden; 8 Early Clinical Development, Research and Early Development, Cardiovascular, Renal and Metabolism, BioPharmaceuticals R&D, AstraZeneca, Gaithersburg, MD USA; 9 Late-stage Development, Cardiovascular, Renal and Metabolism, BioPharmaceuticals R&D, AstraZeneca, Gaithersburg, MD USA; 10 Centre of Metabolism, Ageing and Physiology, Royal Derby Hospital Centre, School of Medicine, University of Nottingham, Derby, UK; 11 Late-stage Development, Cardiovascular, Renal and Metabolism, BioPharmaceuticals R&D, AstraZeneca, Gothenburg, Sweden

**Keywords:** Type 2 diabetes, Drug development, Randomized controlled trials, Type 2 diabetes

## Abstract

Cotadutide is a dual glucagon-like peptide 1 and glucagon receptor agonist under development for the treatment of non-alcoholic steatohepatitis and type 2 diabetes mellitus (T2DM) and chronic kidney disease. Non-alcoholic steatohepatitis is a complex disease with no approved pharmacotherapies, arising from an underlying state of systemic metabolic dysfunction in association with T2DM and obesity. Cotadutide has been shown to improve glycaemic control, body weight, lipids, liver fat, inflammation and fibrosis. We conducted a two-part, randomized phase 2a trial in men and women with overweight or obesity diagnosed with T2DM to evaluate the efficacy and safety of cotadutide compared with placebo and liraglutide. The primary endpoints were change from baseline to day 28 of treatment in postprandial hepatic glycogen (part A) and to day 35 of treatment in fasting hepatic glycogen (part B) with cotadutide versus placebo. Secondary endpoints in part B were changes in fasting hepatic glycogen with cotadutide versus the mono glucagon-like peptide 1 receptor agonist, liraglutide, and change in hepatic fat fraction. The trial met its primary endpoint. We showed that cotadutide promotes greater reductions in liver glycogen and fat compared with placebo and liraglutide. Safety and tolerability findings with cotadutide were comparable to those of previous reports. Thus, this work provides evidence of additional benefits of cotadutide that could be attributed to glucagon receptor engagement. Our results suggest that cotadutide acts on the glucagon receptor in the human liver to promote glycogenolysis and improve the metabolic health of the liver. ClinicalTrials.gov registration: NCT03555994.

## Abstract (web summary)

In a two-part randomized phase 2a trial in men and women with overweight or obesity and type 2 diabetes mellitus, cotadutide promoted greater reductions in liver glycogen and fat than placebo and liraglutide.

## Main

Preclinical studies have shown that cotadutide (MEDI0382) potently activates glucagon-like peptide 1 (GLP-1) and glucagon receptors in a ratio of approximately 5:1, respectively, with the activity of GLP-1 effectively counterbalancing glucagon-driven hepatic glucose production^1–4^. As well as eliciting glycaemic control and weight loss in preclinical models, cotadutide reduced hepatic glycogen, fat, inflammation, steatosis and fibrosis, and improved hepatic mitochondrial function in non-alcoholic steatohepatitis rodent models^1,5^. These effects differentiated cotadutide from a mono GLP-1 receptor agonist, revealing the incremental benefit of the combination of GLP-1 and glucagon receptor agonism in the liver^5^. In early phase 2 clinical trials, treatment with cotadutide versus a mono GLP-1 receptor agonist (liraglutide) in patients with type 2 diabetes mellitus (T2DM) and obesity led to clinically relevant reductions in blood glucose, body weight and liver fat, and improvements in liver health markers, including serum transaminases and N-terminal type III collagen pro-peptide compared with GLP-1 receptor agonism alone^4,6–8^. While greater reductions in surrogate biomarkers are suggestive of glucagon receptor agonism, they do not confirm engagement of the glucagon receptor, especially where equal or opposite modes of action coexist. These insights help characterize the mechanism of action and guide the assessment of benefit‒risk profiles during clinical development. In this phase 2a study, we evaluated the effect of cotadutide compared with placebo and liraglutide on hepatic glycogen dynamics using a ^13^C magnetic resonance spectroscopy (MRS)-based approach to estimate liver glycogen, which, to our knowledge, has not previously been used to measure treatment effects in an interventional trial. The aim was to confirm glucagon receptor engagement in the liver and further investigate the beneficial effects on metabolic and hepatic parameters of cotadutide compared with mono GLP-1 receptor agonism. Cotadutide promoted greater reductions in liver glycogen and fat compared with both placebo and liraglutide. Thus, this work provides evidence of additional benefits of cotadutide that could be attributed to glucagon receptor engagement.

**Fig. 1:**
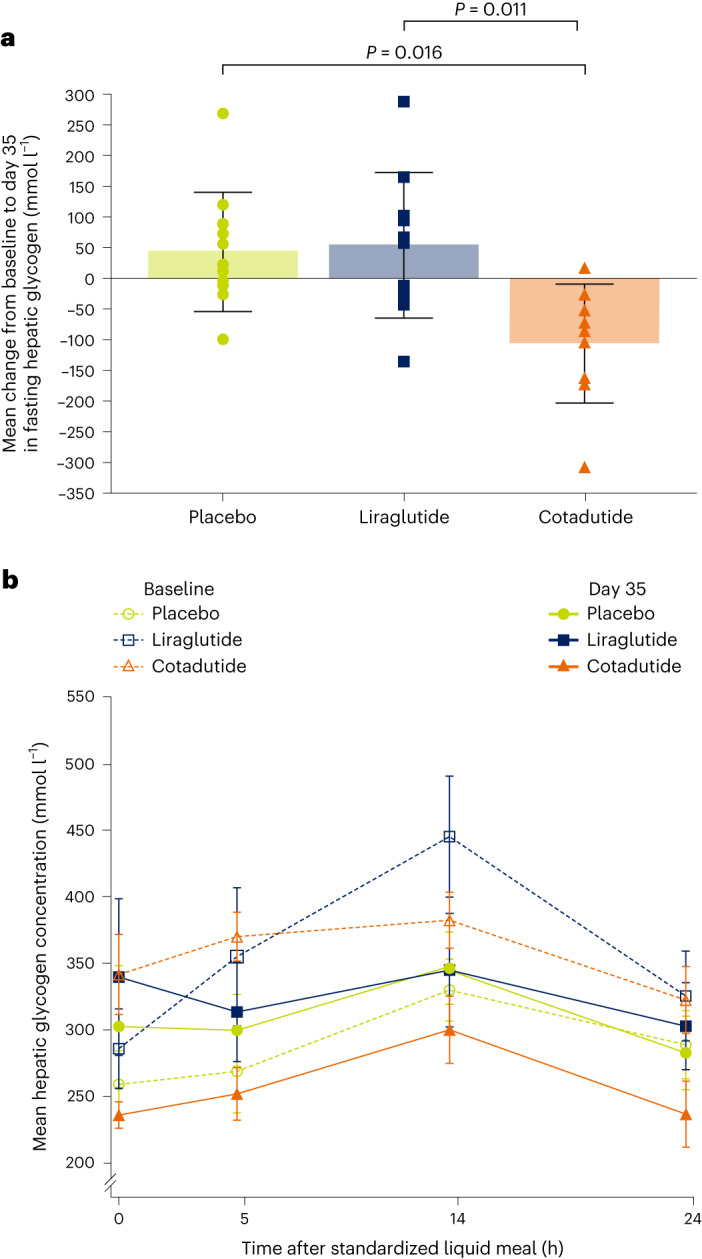
Fasting and postprandial liver glycogen levels (part B). **a**, Mean change from baseline to day 35 of treatment in fasting hepatic glycogen levels in the intent-to-treat population. **b**, Postprandial hepatic glycogen levels across 24 h at baseline and at day 35 of treatment in the intention-to-treat population. Glycogen levels evaluated using ^13^C MRS were adjusted for liver volume. Patients that could be evaluated: placebo, *n* = 11; liraglutide, *n* = 10; cotadutide, *n* = 9. Data in **a** are the mean ± s.d., with overlayed individual data points. Data in **b** are the mean ± s.e.m. In **a**, two-sided *P* values from the analysis of covariance (ANCOVA) model adjusting for baseline value and treatment group are shown, with no corrections for multiple comparisons. Source data

**Extended Data Fig. 1:**
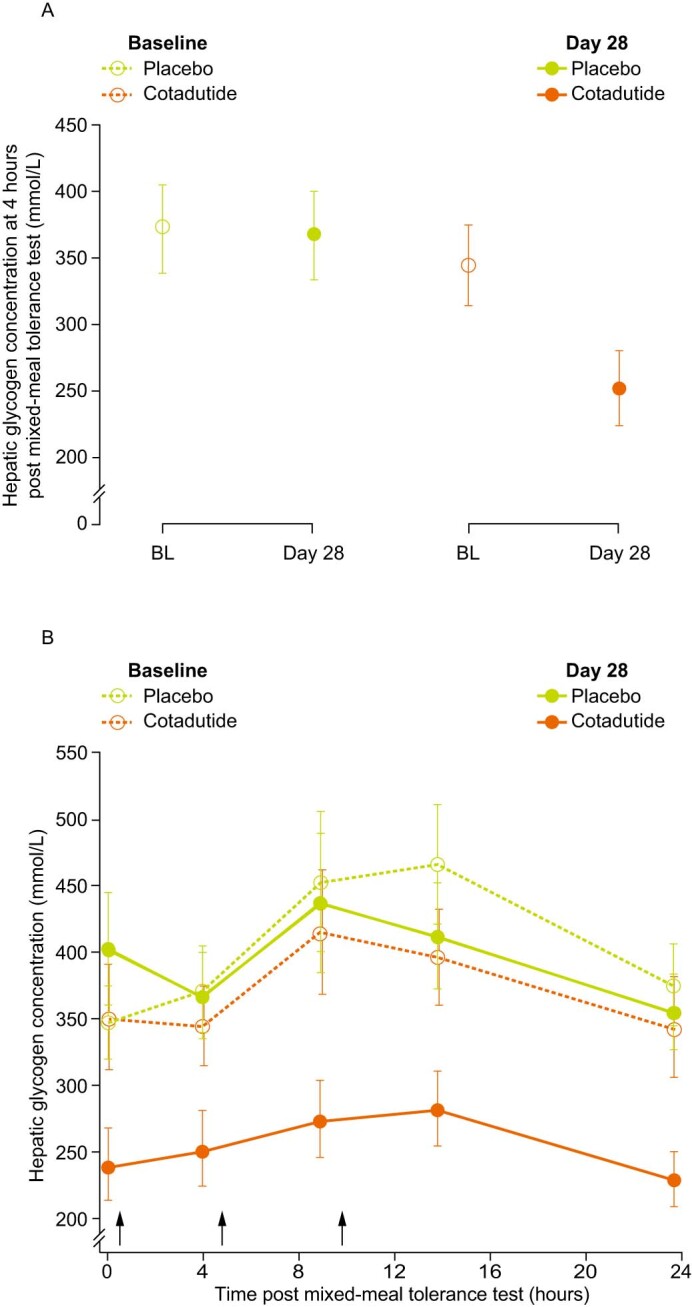
Postprandial glycogen levels at baseline and after 28 days of treatment with cotadutide and placebo (Part A). **a**, Postprandial hepatic glycogen levels at 4 hours after MMTT at baseline and at day 28 of treatment with placebo or cotadutide (part A primary endpoint); **b**, Hepatic glycogen levels across 24 hours at baseline and at day 28 of treatment with placebo or cotadutide. Arrows denote timings of standardized meals. Glycogen levels evaluated by ^13^C MRS were adjusted for liver volume. Evaluable patients: placebo, n = 9; cotadutide, n = 12. Data are means ± SEM. BL, baseline; MMTT, mixed-meal tolerance test; MRS, magnetic resonance spectroscopy; SEM, standard error of the mean.

**Extended Data Fig. 2:**
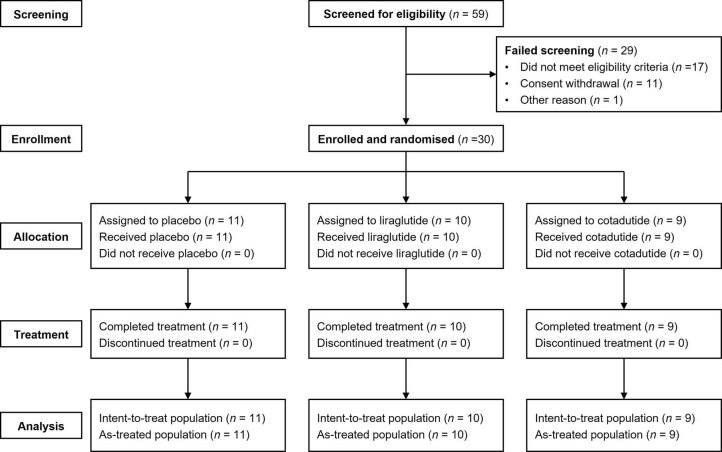
Participant flow diagram (Part B). Flow diagram depicting the number of individuals at each stage of the Part B of the study.

In part A, cotadutide (*n* = 12; see Supplementary Fig. 1 for patient disposition and Supplementary Table 1 for the baseline characteristics) significantly reduced postprandial hepatic glycogen levels compared with placebo (*n* = 9): least squares (LS) mean change from baseline in the cotadutide group was −100.2 mmol l^−1^ (90% confidence interval (CI) = −150.2 to −50.4) versus +5.55 mmol l^−1^ (90% CI = −47.2 to 58.3; *P* = 0.023) in the placebo group (Extended Data Fig. 1; relative change from baseline = −23.6% versus +2.9%). In part B, cotadutide (*n* = 9; see Extended Data Fig. 2 for patient disposition and Extended Data Table 1 for the baseline characteristics) robustly reduced fasting hepatic glycogen by 38% and 41% versus placebo (*n* = 11) and liraglutide (*n* = 10), respectively (Table 1). A significant and physiologically meaningful reduction in LS mean fasting glycogen levels from baseline to day 35 was observed with cotadutide compared with placebo (−129.68 mmol l^−1^ (90% CI = −213.72 to −45.63); *P* = 0.016) and liraglutide (−154.3 mmol l^−1^ (90% CI = −247.7 to −61.0); *P* = 0.011; Table 1 and Fig. 1a). Liraglutide increased fasting glycogen levels after 35 days of treatment (LS mean change from baseline, +51.48 mmol l^−1^ (90% CI = −11.17 to 114.13)). Cotadutide also significantly reduced postprandial LS mean hepatic glycogen levels compared with placebo (−75.05 mmol l^−1^ (90% CI = −114.0 to −36.1); *P* = 0.004) and liraglutide (−63.44 mmol l^−1^ (90% CI = −102.3 to −24.6); *P* = 0.012) (Table 1 and Fig. 1b).

**Table 1:** Primary and exploratory analyses of glycogen levels at the fasting state and postprandial time points (part B)

Time after MMTT	LS mean change from baseline to day 35 (cotadutide versus placebo)		Difference, cotadutide versus placebo	LS mean change from baseline to day 35 (cotadutide versus liraglutide)		Difference, cotadutide versus liraglutide
	Cotadutide (*n* = 9)	Placebo (*n* = 11)		Cotadutide (*n* = 9)	Liraglutide (*n* = 10)	
**Primary outcome**						
**0** **h (fasting)**						
Glycogen concentration, mmol l^−1^	−94.87 (−154.35 to −35.39)	34.81 (−18.41 to 88.02)	−129.68 (−213.72 to −45.63) *P* = 0.016	−102.86 (−169.07 to −36.65)	51.48 (−11.17 to 114.13)	−154.34 (−247.66 to −61.02) *P* = 0.011
**Percentage**	−24.84 (−39.52 to −10.15)	13.11 (−0.03 to 26.25)	−37.95 (−58.70 to −17.20) *P* = 0.005	−25.69 (−42.76 to −8.63)	15.78 (−0.37 to 31.92)	−41.47 (−65.52 to −17.42) *P* = 0.008
**Exploratory outcomes**						
**5** **h**						
Glycogen concentration, mmol l^−1^	−95.15 (−133.77 to −56.54)	12.12 (−22.24 to 46.49)	−107.27 (−162.95 to −51.59)	−114.80 (−149.78 to −79.82)	−44.38 (−77.56 to −11.20)	−70.42 (−118.69 to −22.15)
**Percentage**	−23.10 (−39.13 to −7.07)	11.36 (−2.91 to 25.62)	−34.45 (−57.57 to −11.34)	−31.50 (−40.69 to −22.32)	−7.57 (−16.29 to 1.14)	−23.93 (−36.60 to −11.26)
**14** **h**						
Glycogen concentration, mmol l^−1^	−73.47 (−115.49 to −31.46)	9.2 (−28.54 to 46.95)	−82.68 (−141.06 to −24.29)	−94.57 (−146.91 to −42.22)	−89.19 (−138.74 to −39.64)	−5.38 (−78.91 to 68.16)
**Percentage**	−20.42 (−32.61 to −8.23)	5.36 (−5.59 to 16.31)	−25.78 (−42.72 to −8.84)	−23.47 (−35.13 to −11.81)	−18.40 (−29.44 to −7.37)	−5.07 (−21.45 to 11.31)
**24** **h**						
Glycogen concentration, mmol l^−1^	−83.18 (−111.76 to −54.60)	−8.13 (−33.92 to 17.67)	−75.05 (−114.00 to −36.11)	−85.92 (−114.11 to −57.73)	−22.47 (−49.22 to 4.27)	−63.44 (−102.30 to −24.59)
**Percentage**	−27.02 (−38.04 to −16.01)	−1.15 (−11.09 to 8.79)	−25.87 (−40.88 to −10.86)	−27.31 (−36.42 to −18.20)	−5.33 (−13.97 to 3.32)	−21.99 (−34.55 to −9.43)
**AUC**_**24** **h**_						
**Percentage**	−26.08 (−35.46 to −16.71)	6.72 (−1.67 to 15.10)	−32.80 (−46.03 to −19.57)	−27.05 (−35.22 to −18.89)	−12.18 (−19.92 to −4.44)	−14.88 (−26.14 to −3.61)

Change from baseline to day 35 of treatment and percentage change from baseline in ^13^C MRS-assessed glycogen concentration in the liver at specified time points before and after an MMTT (adjusted for liver volume). All data are the LS mean (90% CI). Two-sided *P* values from an analysis of covariance with effect for the treatment group and baseline as a covariate.

**Fig. 2:**
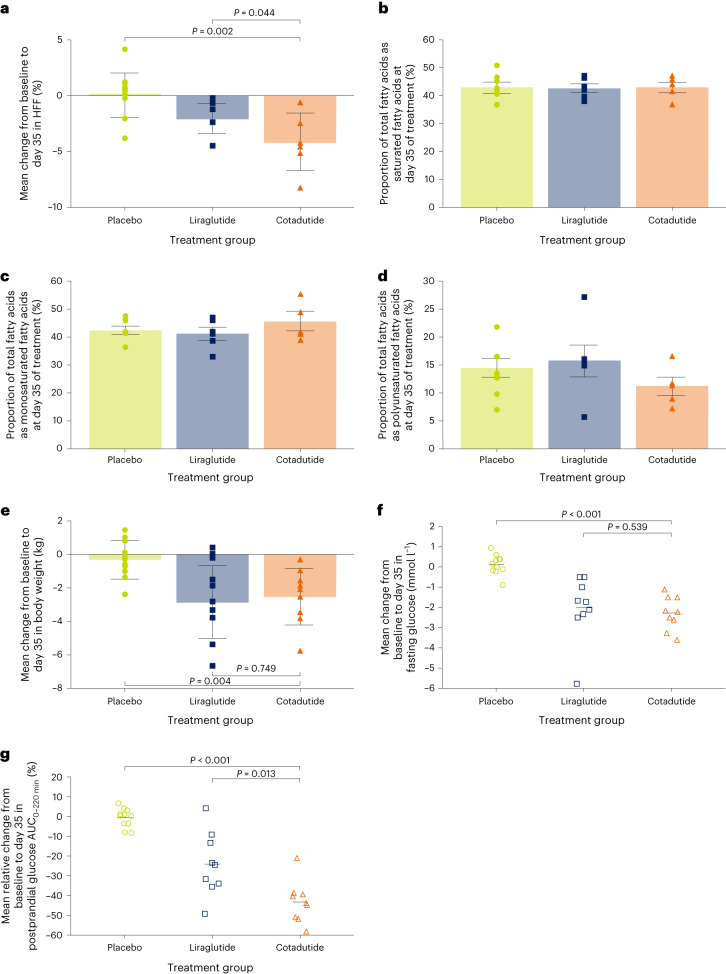
HFF, hepatic fatty acid composition and body weight, and fasting and postprandial blood glucose (part B). **a**, Mean change from baseline to day 35 of treatment in fasting HFF, measured using MRI–PDFF. **b**–**d**, Proportional composition of fatty acids in the liver at day 35 of treatment. **e**, Mean change from baseline to day 35 of treatment in body weight. **f**, Mean change from baseline to day 35 of treatment in fasting plasma glucose. **g**, Mean relative change from baseline to day 35 of treatment in postprandial plasma glucose (AUC_0–220 min_) after the MMTT. Patients who were evaluated in **a**: placebo, *n* = 10; liraglutide, *n* = 7; cotadutide, *n* = 6. Patients who were evaluated in **b**–**d**: placebo, *n* = 7; liraglutide, *n* = 6; cotadutide, *n* = 5. Patients who were evaluated in **e**: placebo, *n* = 11; liraglutide, *n* = 10; cotadutide, *n* = 9. Patients who were evaluated in **f** and **g**: placebo, *n* = 11; liraglutide, *n* = 9; cotadutide, *n* = 9. Data in **a** and **e** are the mean ± s.d., with overlayed individual data points. Data in **b**–**d** are the mean ± s.e.m. Data in **f** and **g** are the mean and overlayed individual data points. Two-sided *P* values from the ANCOVA model adjusting for baseline value and treatment group are shown, with no corrections for multiple comparisons. Source data

**Extended Data Fig. 3:**
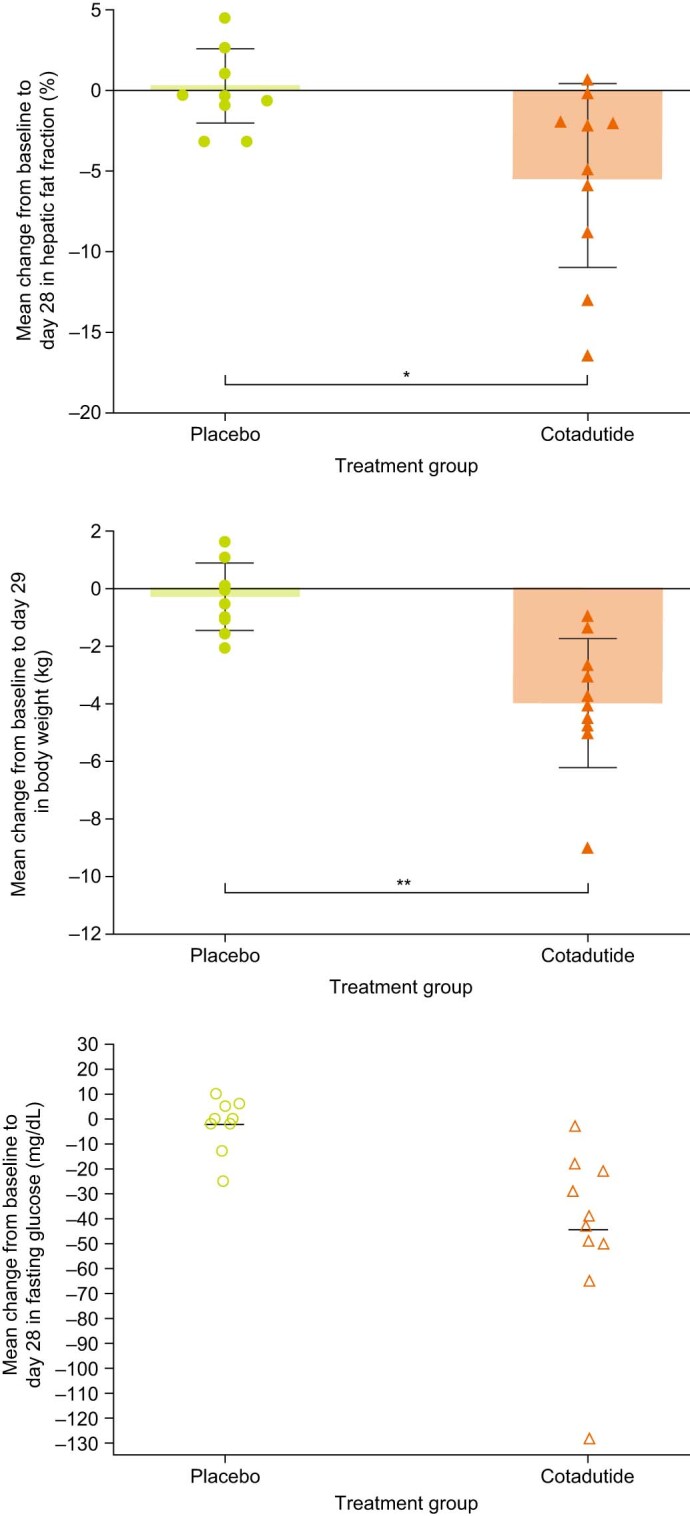
Hepatic fat fraction, body weight and plasma glucose (Part A). **a**, Mean change from baseline to day 28 of treatment in fasting hepatic fat fraction measured by MRI-PDFF. Evaluable patients: placebo, *n* = 9; cotadutide, *n* = 10. Data are individual data points and means ± SD. * *P* < 0.01. **b**, Mean change from baseline to day 29 of treatment in body weight. Evaluable patients: placebo, *n* = 9; cotadutide, *n* = 10. Data are individual data points and means ± SD. ** *P* < 0.001. **c**, Mean change from baseline to day 28 of treatment in fasting plasma glucose. Mean change from baseline to day 28: placebo, ‒2.3 mg/dl (SD, 10.7); cotadutide, ‒44.5 mg/dl (SD, 34.5). Evaluable patients: placebo, n = 9; cotadutide, n = 10. Data are individual data points and mean (horizontal bar). Two-sided P-values from ANCOVA model adjusting for baseline value and treatment group are shown, with no corrections for multiple comparisons applied. MRI-PDFF, magnetic resonance imaging–estimated proton density fat fraction; SD, standard deviation.

We used magnetic resonance imaging–proton density fat fraction (MRI–PDFF) to evaluate liver fat. Treatment with cotadutide for 35 days resulted in a significant absolute reduction in hepatic fat fraction (HFF) compared with both placebo (LS mean absolute change from baseline, −4.1% (90% CI = −6.0 to −2.3); *P* = 0.002) and liraglutide (LS mean absolute change from baseline, −1.8% (90% CI = −3.1 to −0.4); *P* = 0.044; Fig. 2a). These changes corresponded to a 35.1% and 11.7% relative reduction in HFF versus placebo and liraglutide, respectively. The effect versus placebo was similar to that observed in part A (Extended Data Fig. 3a). A post hoc analysis of fatty acids in the liver revealed a nominally lower percentage of polyunsaturated fatty acids and higher percentage of monounsaturated fatty acids in patients treated with cotadutide compared with placebo and liraglutide (Fig. 2b–d).

To explore whether the differential effect of cotadutide versus liraglutide on hepatic glycogen and fat fraction was associated with other health benefits, we analysed body weight and blood glucose during a mixed-meal tolerance test (MMTT). In part B, the mean reduction in body weight from baseline to day 35 was similar for cotadutide versus liraglutide (cotadutide, −2.50 kg (90% CI = −3.34 to −1.66); liraglutide, −2.80 kg (−3.91 to −1.69); *P* = 0.749; Fig. 2e). The effect of cotadutide on body weight was similar to that observed in part A after 29 days of treatment (−4.03 kg (90% CI = −5.02 to −3.04); Extended Data Fig. 3b). Fasting glucose reductions with cotadutide and liraglutide treatment were similar (Fig. 2f), with an LS mean change from baseline to day 35 of −2.23 mmol l^−1^ (90% CI = −2.58 to −1.88) and −2.05 mmol l^−1^ (90% CI = −2.40 to −1.71), respectively; *P* = 0.539. However, cotadutide significantly reduced relative postprandial blood glucose area under the curve (AUC)_0–220 min_ a further 12.5% compared with liraglutide (*P* = 0.013; Fig. 2g). Cotadutide promoted significant reductions in fasting and postprandial glucose versus placebo in parts A (Extended Data Fig. 3c) and B (Fig. 2f,g).

To evaluate whether cotadutide and liraglutide had differential effects on other indices of liver health, we performed post hoc analyses on other metabolic and functional parameters associated with liver health. Treatment with cotadutide led to numerical reductions in lipids, free fatty acids (FFAs) and liver biomarkers compared with placebo and liraglutide (Extended Data Table 2 and Supplementary Table 2). Cotadutide also led to nominal reductions in lysine and alanine compared with placebo and liraglutide.

**Extended Data Fig. 4:**
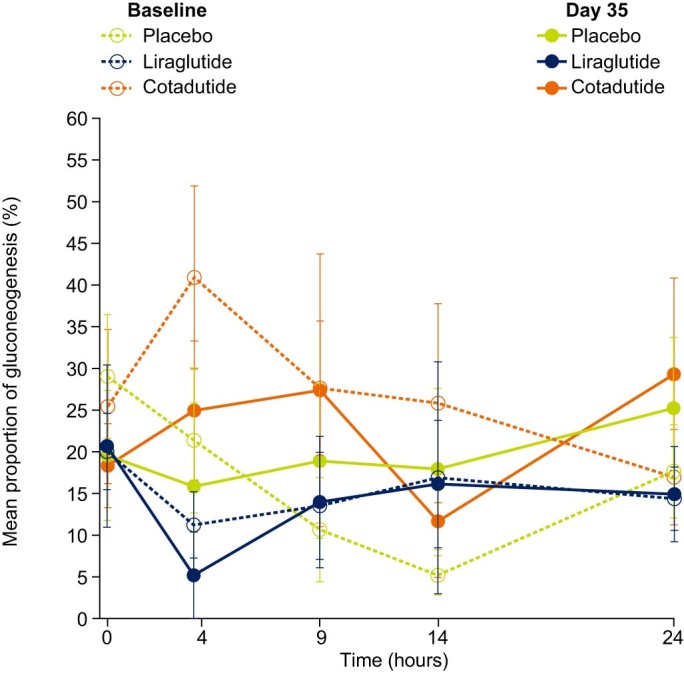
Fasting and postprandial gluconeogenesis measured following ingestion of deuterated water (Part B). Evaluable patients: placebo, *n* = 11; liraglutide, *n* = 10; cotadutide, *n* = 9. Data are means ± SD. SD, standard deviation. This analysis was not performed in Part A of the study.

We evaluated the relative contribution of gluconeogenesis and glycogenolysis to the glucose pool after ingestion of deuterated water. Overall, there were no significant differences in the percentage contribution of gluconeogenesis or glycogenolysis to the glucose pool after treatment with cotadutide, although less postprandial suppression of gluconeogenesis was evident with cotadutide compared with liraglutide and placebo (Extended Data Fig. 4).

Safety and tolerability findings with cotadutide were comparable to those of previous reports (Table 2, Extended Data Table 3 and Supplementary Tables 3 and 4)^4,6,7^. The incidence of treatment-emergent adverse events (TEAEs) in part B was similar with liraglutide and cotadutide (8 of 10 (80%) versus 7 of 9 patients (77.8%); Table 2) and similar to part A (Supplementary Table 3).

**Table 2:** TEAEs during the study (part B)

*n* (%)	Placebo (*n* = 11)	Liraglutide (*n* = 10)	Cotadutide (*n* = 9)
Any TEAE, *n* (%)			
Any grade of TEAE	6 (54.5)	8 (80.0)	7 (77.8)
Treatment-related	3 (27.3)	7 (70.0)	7 (77.8)
Grade ≥3 TEAE	0	0	0
Serious TEAE	0	0	0
Deaths	0	0	0
TEAEs leading to treatment discontinuation	0	0	0
TEAEs occurring at a frequency ≥20% in any group^a^			
Nausea	0	1 (10.0)	5 (55.6)
Fatigue	0	1 (10.0)	3 (33.3)
Constipation	0	2 (20.0)	2 (22.2)
Insomnia	0	0	2 (22.2)
Gastro-oesophageal reflux disease	0	2 (20.0)	0
Decreased appetite	0	2 (20.0)	0
Dizziness	1 (9.1)	2 (20.0)	1 (11.1)
Pollakiuria (urinary frequency syndrome)	2 (18.2)	0	0
Headache	1 (9.1)	1 (10.0)	1 (11.1)
Immunogenicity			
ADA^+^ after baseline, *n* (%)	0	N/A	3 (33.3)
*n*	0	N/A	3
Median of maximum titre^b^	N/A	N/A	40
IQR	N/A	N/A	20–80
Treatment-boosted ADA after baseline, *n* (%)	0	N/A	0

Patients were counted once for each system organ class and preferred term regardless of the number of events. ADA, anti-drug antibody; IQR, interquartile range; MedDRA, Medical Dictionary for Regulatory Activities; N/A, not applicable.

^a^Preferred term (MedDRA v.24.0).

^b^Includes all ADA^+^ assessments with reportable ADA titre results after baseline.

Our findings indicate that treatment with the dual GLP-1 and glucagon receptor agonist cotadutide improves metabolic and functional parameters in the liver compared with placebo and liraglutide in patients with T2DM and overweight or obesity. Cotadutide treatment resulted in a significant and physiologically meaningful reduction in hepatic glycogen compared with liraglutide and placebo, confirming engagement of glucagon receptors in the liver to stimulate glycogenolysis. This was accompanied by a reduction in liver fat and plasma lipids and numerical improvements in other indices of liver health. These results were consistent with part A of the study, performed independently in a patient population with similar characteristics.

Evaluating hepatic glycogen dynamics using a ^13^C MRS-based approach confirmed the differential mechanism of action of dual receptor agonism over mono GLP-1 receptor agonism. A study with similar aims of the dual GLP-1 and glucagon receptor agonist SAR425899 used tracer-enhanced positron emission tomography–computed tomography to evaluate glucagon receptor engagement but did not confirm occupancy. This may have been inherent to the pharmacology of this peptide, but the methodology could not rule out that receptor internalization limited the interpretation of these results^8^.

Cotadutide promoted greater reductions in steatosis than liraglutide despite comparable body weight loss, which is consistent with observations from previous clinical and preclinical studies, highlighting differentiation of the dual receptor agonist^5,7^. We postulate that glucagon receptor-driven increases in hepatic fatty acid oxidation and suppression of lipogenesis account for this observation, whereas GLP-1 receptor effects are indirect and due to body weight loss because GLP-1 receptors are not highly expressed in the human liver^9^. Flux measures of FFAs, cholesterol and glycerol would be required to evaluate this further. A limitation of this comparison was the difference in baseline body mass index (BMI) and HFF between study arms (Extended Data Tables 1 and 2), although baseline differences were accounted for in statistical analyses. In addition, 4 of 30 patients (13%) in part B had PDFF scans at baseline that could not be evaluated.

A global reduction in fasting and postprandial liver glycogen was observed in cotadutide-treated patients compared with placebo and liraglutide. Effects on fasting glycogen are unrelated to changes in weight loss because fasting plasma glucose and body weight reduction were similar between cotadutide- and liraglutide-treated patients. Furthermore, studies indicated that glycogen is a dynamic pool influenced by acute nutrient availability rather than longer-term body weight status^10^. While a strength of this study was the use of standardized meals in the days preceding the scans, with comparable body weight and glucose changes in the cotadutide and liraglutide arms, a limitation was the inability to control for other factors that may influence glycogenolysis, such as endogenous hormonal changes and substrate supply. Hepatic glycogen stores were not completely depleted after cotadutide treatment (reduced to 236.1 mmol l^−1^ at day 35), implying that sufficient glycogen substrate is available for mobilization of glucose during hypoglycaemia.

Cotadutide induced a greater postprandial reduction in glucose compared with placebo and liraglutide. This could, in part, explain lower postprandial glycogen levels where prolonged gastric emptying time may have led to delayed hepatic glucose uptake. However, serial measures of hepatic glycogen over a 24-h period were performed in this study: a delay in glucose delivery to the liver might be expected to induce a rightward shift of the glycogen time curve but this was not observed. A numerical reduction from baseline in hepatic glycogen was observed at 14 h in the liraglutide arm and was probably attributable to a single patient who was unable to complete standardized meals after treatment.

The GLP-1 and glucagon activity ratio in cotadutide was optimized to achieve a maximally beneficial overall effect from the agonism of each receptor^7^. Observed reductions in fasting and postprandial glucose and glycogen levels demonstrate that cotadutide effectively engages the GLP-1 and glucagon receptors. Glucagon receptor-driven glycogenolysis predominates over GLP-1 and insulin-mediated glycogen synthesis; despite this, a net reduction in glucose was observed in the fasted state. Although diurnal variation may occur, it is probable that GLP-1 receptor agonism and insulin release counter increased hepatic glucose output via enhanced glucose uptake to tissues. However, we cannot exclude that in cotadutide-treated patients GLP-1 and insulin-mediated suppression of gluconeogenesis compensates for increased glycogenolysis.

Greater reductions in lactate and alanine with cotadutide treatment versus liraglutide may be suggestive of increased consumption of gluconeogenic substrates; however, no changes in the relative contribution of gluconeogenesis versus glycogenolysis to the glucose pool were detected. This could imply that cotadutide has equipotent effects on gluconeogenesis and glycogenolysis but may also reflect limitations in the analysis of gluconeogenesis in this study, which was underpowered and potentially used an insufficient dose of deuterated water.

In conclusion, treatment with cotadutide in patients with T2DM and overweight or obesity led to significant and physiologically meaningful reductions in liver glycogen and fat compared with placebo and liraglutide, supporting engagement with the glucagon receptor in the human liver to promote offloading of excess energy substrates. Our findings confirm that dual agonism promotes hepatic, metabolic and body weight loss benefits associated with the combined action of glucagon and GLP-1, which could directly underpin the therapeutic effect as a potential treatment for metabolic diseases. This study demonstrates the dual human pharmacology of a unimolecular dual receptor agonist peptide. Dedicated studies in populations with confirmed non-alcoholic steatohepatitis should explore this potential further.

## Methods

### Study conduct

The study was conducted in accordance with the principles of the Declaration of Helsinki (2013), the International Council for Harmonization Guidance for Good Clinical Practice and was approved by the independent ethics committees at Linköping University, Linköping, Sweden, and Maastricht University Medical Centre, Maastricht, the Netherlands. The national regulatory authorities in each country were notified and approved the study. Written informed consent forms and any other written information and materials to be provided to participants were approved by the independent ethics committees. Written informed consent from all participants was obtained before enrolment into the trial. The study is registered at ClinicalTrials.gov (NCT03555994).

### Study design

**Extended Data Fig. 5:**
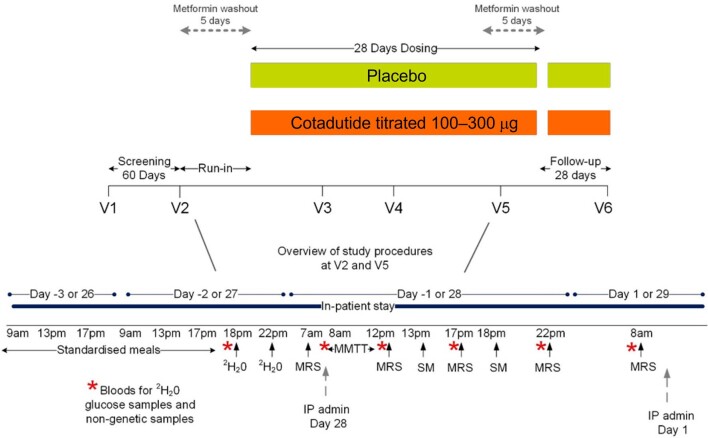
Study design (Part A). Study flow diagrams and assessment schedule for Part A. ^2^H_2_O, deuterated water (administration); IP, investigational product; MMTT, mixed-meal tolerance test; MRS, magnetic resonance spectroscopy; SM, standardized meal; V, visit.

**Extended Data Fig. 6:**
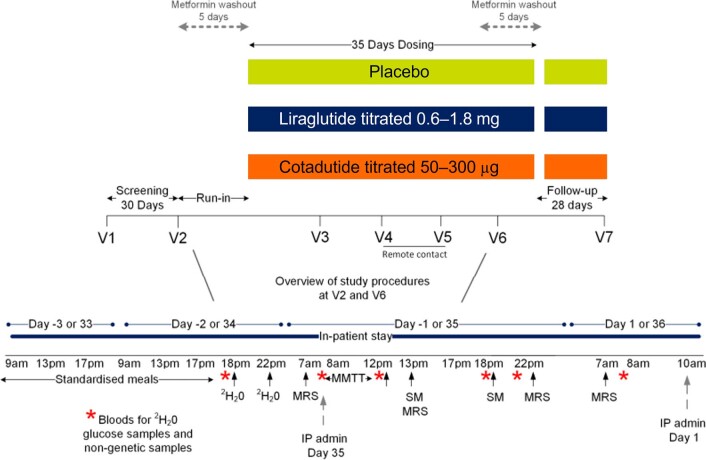
Study design (Part B). Study flow diagrams and assessment schedule for Part B. ^2^H_2_O, deuterated water (administration); IP, investigational product; MMTT, mixed-meal tolerance test; MRS, magnetic resonance spectroscopy; SM, standardized meal; V, visit.

This two-part, randomized, parallel-group phase 2a study was conducted sequentially between June 2018 and April 2021 at two study sites in Sweden and the Netherlands. Part A was an exploratory, double-blind, randomized, placebo-controlled study (Extended Data Fig. 5). Achievement of the primary objective of part A determined continuation into part B, which was a part-blinded, randomized, active-comparator study (Extended Data Fig. 6). Patients were enrolled by study site investigators and randomized after assessment of study eligibility was complete. Patients were randomly assigned to treatment groups and investigation product kit numbers by a computer-generated randomized sequence (Covance), with the use of interactive Web response systems, which assigned a unique randomization code and treatment group to the patient. The overall primary objective (parts A and B) was to evaluate the effect of cotadutide on hepatic glycogen levels compared with placebo, as evidence of glucagon receptor engagement. Secondary objectives were to evaluate the effect of cotadutide on hepatic glycogen levels and HFF compared with liraglutide (part B).

Eligible patients for both parts were adults with a BMI of 27–40 kg m^−^^2^ and T2DM (HbA1c ≤ 8.0% (64 mmol mol^−1^)) receiving metformin monotherapy. Exclusion criteria included previous use of GLP-1 receptor analogue-based therapy, daily insulin and a history of heavy alcohol use. Participants required a negative alcohol test at screening and randomization.

This study included both sexes; gender data were not collected. Owing to the small sample size, sex was not considered in the study design and data were not disaggregated for sex. Ethnicity, ancestry or any other socially relevant groupings were also not considered in the study design.

### Treatment and assessment

In part A, patients were randomized 1:1 to receive either subcutaneous once-daily cotadutide titrated 100–300 μg or matching placebo for 28 days (Extended Data Fig. 4a). In part B, patients were randomized 1:1:1 to receive either subcutaneous once-daily cotadutide titrated 50–300 μg, subcutaneous once-daily liraglutide titrated 0.6–1.8 mg or placebo for 35 days.

Part A was double-blinded, with both investigators, patients and sponsor staff involved in the treatment or clinical evaluation of patients unaware of treatment allocation. An unblinded site monitor, who was not involved in treatment or clinical evaluation of patients, performed investigational product accountability. Part B was a part-blinded, randomized, active-comparator study, with investigators and patients unaware of allocation to either placebo or cotadutide; however, liraglutide was open-label. The cotadutide and placebo multidose pens were indistinguishable.

Hepatic glycogen, HFF, blood glucose, gluconeogenesis and vitals were assessed at baseline and at the end of treatment. A safety follow-up visit took place 28 days after the administration of the last treatment.

Participants underwent a 5-day washout of metformin and were admitted for 3 days before baseline and end of treatment assessment; participants were given identical, standardized meals during admission, without access to additional food or alcohol. Serial measurements of hepatic glycogen levels were performed using ^13^C MRS on 3T MRI scanners (GE Healthcare and Philips Healthcare) at baseline and at the end of treatment before (fasting) and at 5, 14 and 24 h after a liquid mixed meal. HFF was measured using MRI–PDFF (see the extended methods for further details).

#### Imaging assessment

For the ^13^C MRS evaluation of liver glycogen, the signal was generated via a free-induction decay protocol using dedicated ^13^C surface coils (RAPID Biomedical) placed on top of the liver, with the following parameters: flip angle calibrated to 90° at 8-cm depth; no decoupling; repetition time, 280 ms; 4,096 data acquisitions). Quantification was performed by calibrating to a glucose solution (natural abundance) with known glucose concentration and via the inclusion of small reference vials (^13^C-enriched acetone or acetate) placed on top of the coil casing during patient scanning. Peak areas were determined automatically using MATLAB 2014b (MathWorks).

Hepatic volumes were assessed from two-point Dixon water images at each visit. All glycogen levels were adjusted for changes in liver volume to correct for diurnal variations and for changes over the treatment period.

HFF was assessed using whole-liver MRI–PDFF via six-point Dixon scanning at baseline and on day 35 of treatment before a standardized liquid mixed meal. Hepatic fat composition was determined via a single-voxel stimulated echo acquisition mode ^1^H-MRS protocol and controlled breathing as described previously^11^.

#### Deuterated water assessment

Plasma glucose was assessed from blood samples collected at baseline and on day 35 of treatment after an 8-h fast (15 min before consumption of a liquid mixed meal) and at 15, 30, 45, 60, 120, 180 and 220 min after a liquid mixed meal (Ensure Plus, Abbott Nutrition) nutritional supplement containing 86 g carbohydrates (37 g sugars), 18 g fat and 22 g protein; 400 ml; 590 kcal). For plasma analysis of gluconeogenesis, deuterated water (^2^H_2_O, 2.3 ml kg^−1^ body weight) was administered the night before the baseline assessment day and day 35 of treatment; blood samples were collected during fasting and 220 min, and 9, 14 and 24 h after the liquid mixed meal to assess the relative incorporation of deuterium onto carbons C_2_ and C_5_ of newly formed glucose (during which standardized meals were received)^12,13^. In part A, estimates of gluconeogenesis and glycogenolysis could not be obtained because of technical issues. Samples were instead used to support method development and assay validation for part B.

### Endpoints

The primary endpoint in part A was change from baseline to day 28 in postprandial hepatic glycogen with cotadutide versus placebo and was selected based on preclinical findings^5^. Results from part A showed that measurement of fasting rather than postprandial glycogen was optimal from an operational and sample size perspective. Therefore, the primary endpoint of part B was change from baseline to day 35 of treatment in fasting hepatic glycogen adjusted for liver volume (Extended Data Table 4 and Supplementary Table 5) with cotadutide versus placebo. Secondary endpoints were change from baseline to day 35 of treatment in fasting hepatic glycogen adjusted for liver volume and HFF with cotadutide versus liraglutide. Exploratory endpoints in both parts were change from baseline to end of treatment in body weight, fasting and postprandial blood glucose, lipids, FFAs, amino acids and liver biomarkers compared with placebo (and liraglutide in part B) and change from baseline to day 35 of treatment in postprandial hepatic glycogen adjusted for liver volume compared with placebo and liraglutide (part B only).

### Statistical analyses

In part A, the sample size of eight in each study group would provide more than 80% power to detect 19% difference between treatment groups for the primary endpoint, with a two-sided significance level of 0.1 (corresponding to 90% CI, selected to minimize sample size in this exploratory study), assuming a mean baseline glycogen concentration of 283 μmol ml^−1^ (s.d. = 41 μmol ml^−1^) for both groups^14,15^. In part B, the sample size of ten in the cotadutide and placebo groups would provide more than 80% power to detect 24.3% difference between treatment groups for the primary endpoint, with a two-sided significance level of 0.1, assuming an s.d. of 17% for both groups.

Primary efficacy analyses were performed on the intent-to-treat population; safety analyses were performed on the as-treated population. All available data were included in the analyses and missing data were not imputed except as specified in the calculation of the AUC values. For the secondary and exploratory endpoints, only patients who could be evaluated with valid baseline or after baseline measurements were analysed. Changes from baseline were assessed using an ANCOVA model, with treatment group and baseline value as covariates. Separate models comparing cotadutide versus placebo and cotadutide versus liraglutide were generated; adjustments for multiple comparisons were not performed. Summary or descriptive statistics were generated for the analysis of clinical laboratory parameters and vital signs. For primary and secondary endpoints, the assumptions of each ANCOVA model were assessed and confirmed to be valid. Formal statistical testing for exploratory endpoints was not performed. Data analyses were performed using SAS v.9.3 or higher (SAS Institute) within a UNIX environment.

### Reporting summary

Further information on research design is available in the Nature Portfolio Reporting Summary linked to this article.

### Supplementary information

Supplementary Information Supplementary Fig. 1 and Tables 1–5.

Reporting Summary

Supplementary Data CONSORT checklist.

### Source data

Source Data Figs. 1 and 2 and Table 1 Figs. 1 and 2 and Table 1: Statistical source data – adjusted liver glycogen concentration and hepatic fat fraction at baseline and after 35 days of treatment (fasting and postprandial time points) in all patients (indicated by a unique identifier) without treatment assignment. Fig. 2: Source data for monounsaturated, saturated and polyunsaturated fatty acid data in individual patients according to treatment group.

## Figures and Tables

**Extended Data Table 1:**
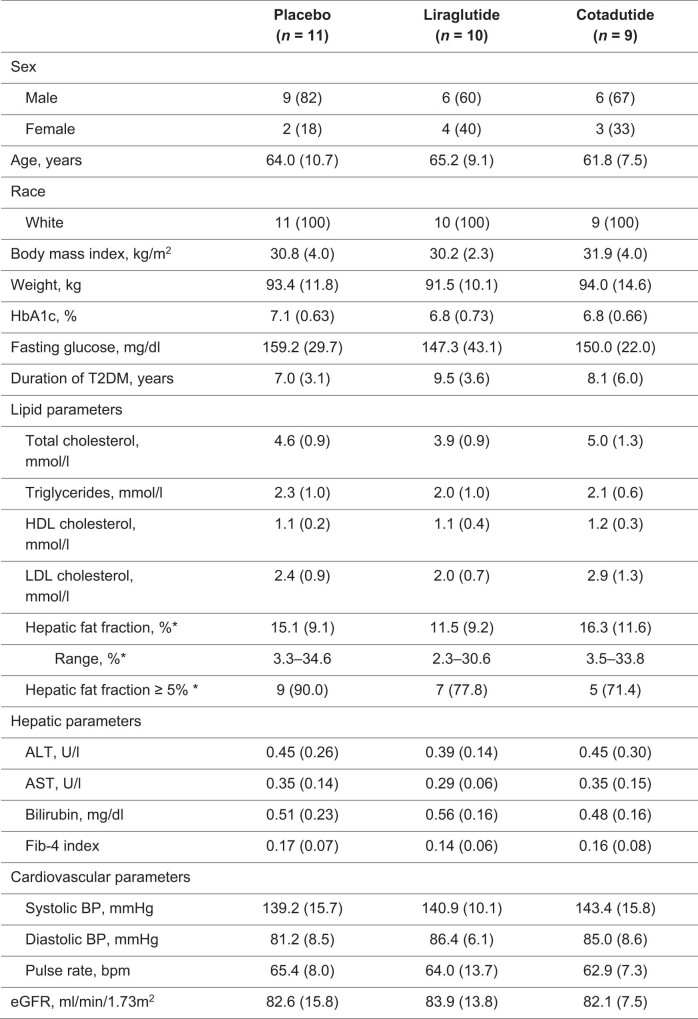
Patient demographics and baseline characteristics (part B)

*Evaluable patients: placebo, *n* = 10; liraglutide, *n* = 9, cotadutide, *n* = 7. All categorical variables are *n* (%) and all continuous variables are mean (SD). ALT, alanine aminotransferase; AST, aspartate aminotransferase; BP, blood pressure; bpm, beats per minute; eGFR, estimated glomerular filtration rate; HbA1_c_, glycated haemoglobin; HDL, high-density lipoprotein; LDL, low-density lipoprotein; T2DM, type 2 diabetes mellitus.

**Extended Data Table 2:**
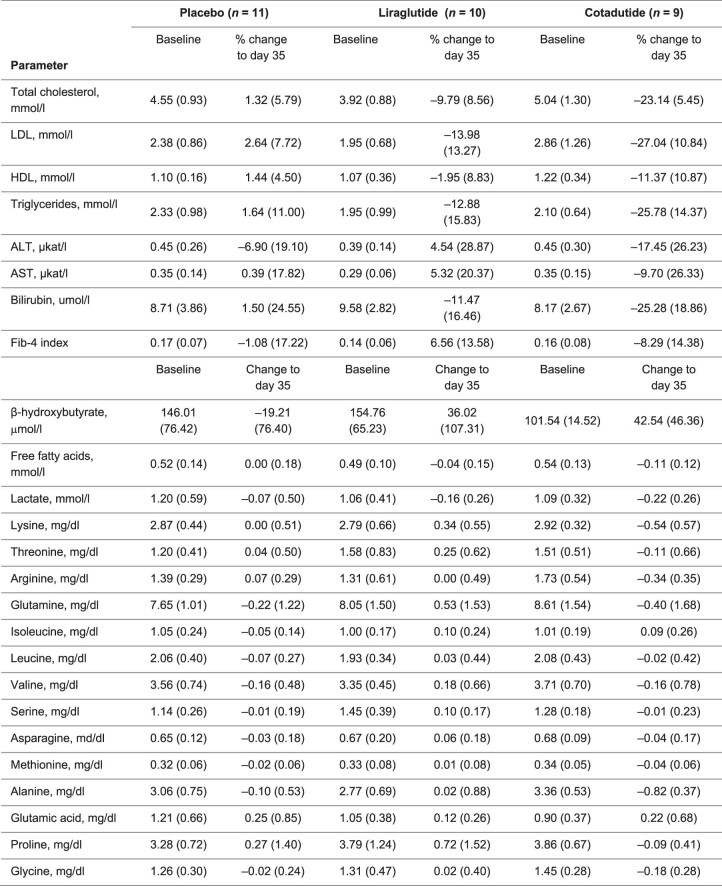
Exploratory metabolic outcomes (part B)

Change from baseline to day 35 of treatment in exploratory outcomes - fasting lipids, free fatty acids, ketones, amino acids and markers of liver health (part B) Data are mean (SD). ALT, alanine aminotransferase; AST, aspartate aminotransferase; Fib-4, fibrosis-4; HDL, high density lipoprotein; LDL, low-density lipoprotein.

**Extended Data Table 3:**
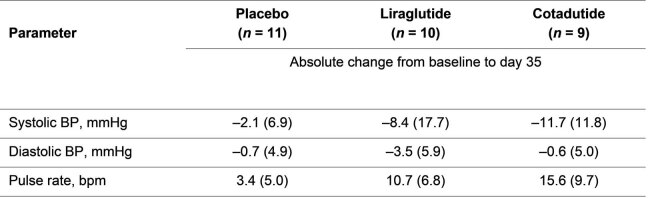
Vitals (part B)

Change from baseline to day 36 in systolic blood pressure, diastolic blood pressure and pulse rate. Data are mean (SD). BP, blood pressure; bpm, beats per minute; ECG, electrocardiogram.

**Extended Data Table 4:**
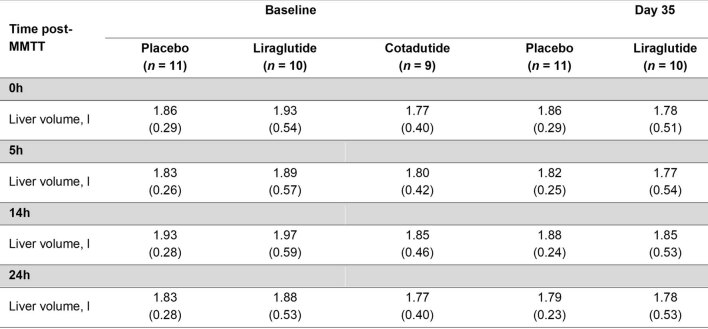
Liver volume (part B)

Liver volume at baseline and day 35 of treatment in part B of the study. Data are mean (SD). MMTT, mixed-meal tolerance test.

## Data Availability

Source files for primary and key secondary outcomes containing data underlying the findings described in this article may be obtained from the corresponding author upon reasonable request in accordance with AstraZeneca’s data sharing policy described at https://astrazenecagrouptrials.pharmacm.com/ST/Submission/Disclosure. Source data are provided with this paper.
