# Supplementary material for: Cotadutide promotes glycogenolysis in people with overweight or obesity diagnosed with type 2 diabetes
Source: Nat Metab. 2023 Dec 8;5(12):2086–93. doi: 10.1038/s42255-023-00938-0 (PMC10730390; doi:10.1038/s42255-023-00938-0)
Supplement: Supplementary file 1 — Supplementary Fig. 1 and Tables 1–5. [file 42255_2023_938_MOESM1_ESM.pdf]

nature metabolism

Letter

https://doi.org/10.1038/s42255-023-00938-0

Cotadutide promotes glycogenolysis in people with overweight or obesity diagnosed with type 2 diabetes

In the format provided by the authors and unedited

Supplementary Fig. S1. Patient disposition (Part A)

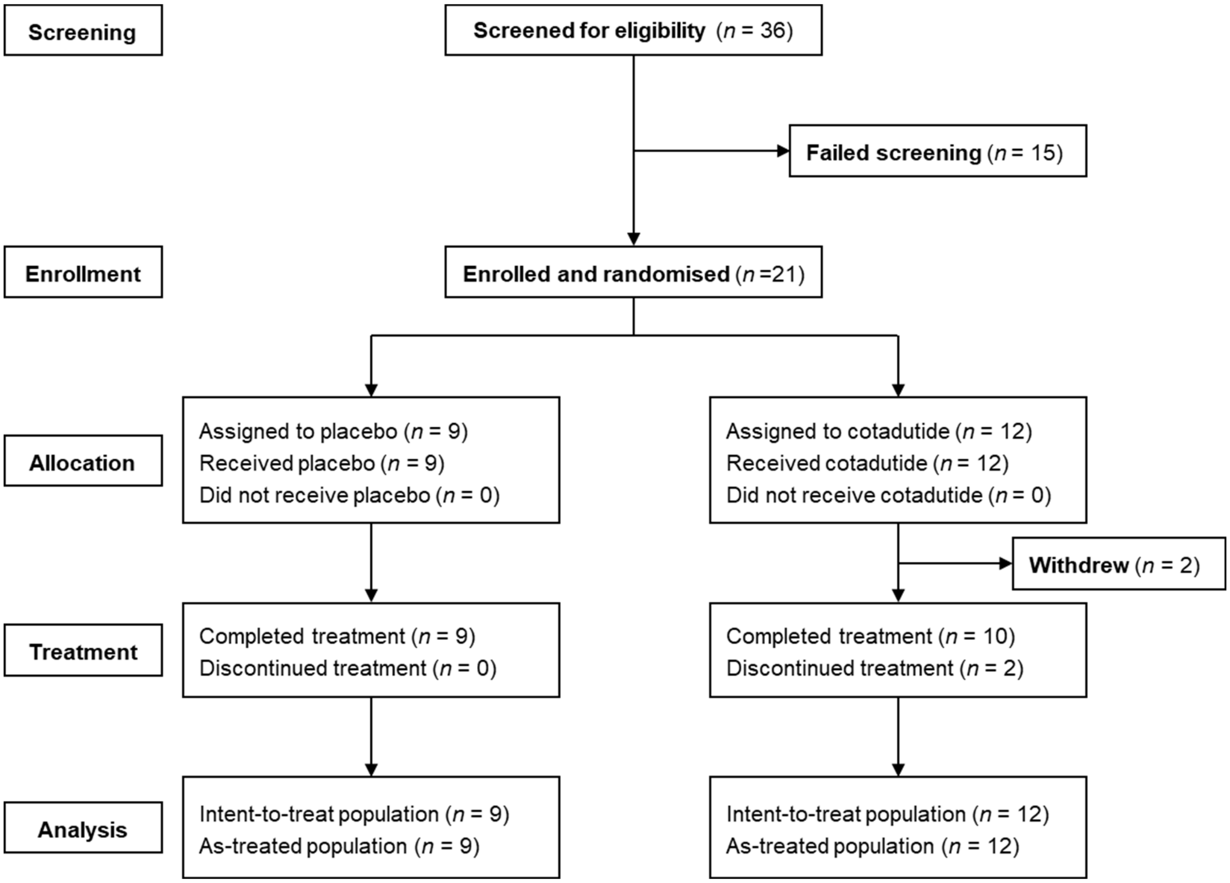

Supplementary Table 1. Demographics and baseline characteristics (Part A)

	**Placebo (*n* = 9)**	**Cotadutide (*n* = 12)**
Sex		
Male	6 (67)	7 (58)
Female	3 (33)	5 (42)
Age, years	69.3 (5.7)	65.8 (7.3)
Race		
White	9 (100)	12 (100)
Body mass index, kg/m^2	32.7 (4.8)	31.8 (3.0)
Weight, kg	99.6 (20.1)	96.2 (7.7)
HbA1c, %	6.3 (0.7)	6.5 (0.6)
Fasting glucose, mg/dl	139.2 (23.1)	153.0 (38.0)
Duration of T2DM, years	9.9 (6.1)	7.7 (5.4)
Lipid parameters		
Total cholesterol, mmol/l	4.9 (1.5)	4.7 (0.8)
Triglycerides, mmol/l	2.4 (1.6)	1.8 (0.8)
HDL cholesterol, mmol/l	1.3 (0.3)	1.3 (0.4)
LDL cholesterol, mmol/l	2.4 (1.1)	2.5 (0.8)
Hepatic fat fraction, %	15.1 (9.1)	16.3 (11.6)
Range of hepatic fat fraction, %	2.9–35.1	2.9–37.9
Hepatic fat fraction ≥ 5%, % evaluable scans	7 (78)	8 (67)
Hepatic parameters		
ALT, U/l	26.1 (13.3)	26.6 (10.6)
AST, U/l	23.2 (9.5)	19.8 (7.4)
Bilirubin, mg/dl	0.54 (0.21)	0.46 (0.18)
Cardiovascular parameters		
Systolic BP, mmHg	136.8 (13.5)	131.6 (9.1)
Diastolic BP, mmHg	77.4 (6.1)	76.9 (8.6)
Pulse rate, bpm	69.3 (8.7)	65.5 (9.5)
eGFR, $ml/min/1.73m^2$	82.8 (13.4)	82.6 (19.3)

All categorical variables are n (%) and all continuous variables are mean (SD). ALT, alanine aminotransferase; AST, aspartate aminotransferase; BP, blood pressure; bpm, beats per minute; eGFR, estimated glomerular filtration rate; $HbA1_c$, glycated haemoglobin; HDL, high-density lipoprotein; LDL, low-density lipoprotein; T2DM, type 2 diabetes mellitus.

Supplementary Table 2. Change from baseline to day 28 of treatment in fasting lipids, free fatty acids, ketones, amino acids and markers of liver health (Part A)

Parameter	Placebo (*n* = 9)		Cotadutide (*n* = 12)	
	Baseline	% change to day 28	Baseline	% change to day 28*
Total cholesterol, mmol/l	4.9 (1.5)	–2.27 (5.37)	4.7 (0.8)	–28.0 (7.59)
LDL, mmol/l	2.35 (1.14)	–1.37 (11.04)	2.54 (0.84)	–30.7 (9.25)
HDL, mmol/l	1.30 (0.32)	–2.10 (6.17)	1.26 (0.44)	–11.49 (8.70)
Triglycerides, mmol/l	2.44 (1.61)	–6.30 (23.33)	1.83 (0.81)	–33.12 (18.23)
ALT, U/l	26.1 (13.3)	–7.7 (16.7)	26.6 (10.6)	–17.5 (21.6)
AST, U/l	23.2 (9.5)	–7.3 (12.8)	19.8 (7.4)	–15.2 (12.2)
Bilirubin, mg/dl	0.54 (0.21)	–2.41 (24.88)	0.46 (0.18)	–2.91 (46.51)
	Baseline	Change to day 28	Baseline	Change to day 28*
Free fatty acids, mmol/l	0.73 (0.15)	–0.09 (0.19)	0.58 (0.10)	–0.06 (0.15)
β-hydroxybutyrate, μmol/l	141.97 (45.23)	30.94 (43.95)	141.72 (62.13)	97.08 (249.15)
Lactate, mmol/l	1.46 (0.66)	–0.20 (0.17)	1.04 (0.23)	–0.34 (0.25)
Lysine, mg/dl	2.82 (0.43)	–0.18 (0.80)	2.93 (0.61)	–1.06 (0.87)
Threonine, mg/dl	1.38 (0.44)	–0.032 (0.29)	1.58 (0.67)	–0.61 (0.67)
Arginine, mg/dl	1.30 (0.29)	–0.26 (0.30)	1.45 (0.31)	–0.60 (0.44)
Glutamine, mg/dl	8.43 (1.39)	–0.98 (1.70)	9.07 (1.57)	–2.22 (2.74)
Isoleucine, mg/dl	0.87 (0.16)	–0.06 (0.18)	0.89 (0.12)	0.08 (0.26)
Leucine, mg/dl	1.94 (0.45)	–0.10 (0.32)	2.14 (0.30)	–0.41 (0.50)
Valine, mg/dl	3.00 (0.55)	–0.14 (0.52)	3.33 (0.51)	–0.75 (0.9)
Serine, mg/dl	1.20 (0.33)	–0.06 (0.24)	1.35 (0.25)	–0.29 (0.33)
Asparagine, md/dl	0.55 (0.11)	–0.07 (0.13)	0.61 (0.10)	–0.16 (0.19)
Methionine, mg/dl	0.34 (0.09)	–0.03 (0.09)	0.34 (0.05)	–0.10 (0.12)
Alanine, mg/dl	3.25 (0.63)	–0.18 (0.62)	3.21 (0.52)	–1.34 (0.76)
Glutamic acid, mg/dl	0.86 (0.66)	–0.05 (0.62)	0.91 (0.36)	–0.46 (0.30)
Proline, mg/dl	2.42 (0.60)	–0.16 (0.72)	2.51 (0.50)	–0.64 (0.63)
Glycine, mg/dl	1.49 (0.35)	0.01 (0.36)	1.80 (0.90)	–0.51 (0.76)

*Evaluable patients *n* = 10

Supplementary Table 3. Treatment-emergent adverse events during the study (Part A)

n (%)	Placebo (*n* = 9)	Cotadutide (*n* = 12)
Any TEAE, n (%)		
Any Grade TEAE	7 (77.8)	11 (91.7)
Treatment-related	5 (55.6)	11 (91.7)
Grade ≥3 TEAE	0	0
Serious TEAE	0	0
Deaths	0	0
TEAE leading to treatment discontinuation	0	2 (16.7)
TEAEs occurring at a frequency ≥20% in any group*		
Nausea	2 (22.2)	8 (66.7)
Fatigue	2 (22.2)	6 (50.0)
Dyspepsia	2 (22.2)	6 (50.0)
Headache	3 (33.3)	5.(41.7)
Constipation	0	3 (25.0)
Decreased appetite	1 (11.1)	3 (25.0)
Dizziness	0	3 (25.0)
Insomnia	0	2 (22.2)
Immunogenicity		
ADA-positive post-baseline, *n* (%)	0	1 (8.3)
n	0	3
Median of maximum titre†	N/A	90
IQR	N/A	80, 80
Treatment-boosted ADA post-baseline, *n* (%)	0	0

Patients were counted once for each System Organ Class and Preferred Term regardless of the number of events.
*Preferred term (MedDRA version 20.0); †Includes all post-baseline, ADA-positive assessments with reportable ADA titre results.
ADA, anti-drug antibody; MedDRA, IQR, interquartile range; Medical Dictionary for Regulatory Activities; TEAE, treatment-emergent adverse event.

Supplementary Table 4. Vitals (Part A)

Parameter	Placebo (*n* = 9)	Cotadutide group (*n* = 12)
	Absolute change from baseline to day 28 of treatment	
Systolic BP, mmHg	0.3 (11.7)	–8.2 (11.7)*
Diastolic BP, mmHg	1.0 (4.9)	–2.6 (5.8)*
Pulse rate, bpm	2.7 (9.3)	13.0 (8.3)*

*Evaluable *n* = 10. Data are mean (SD). BP, blood pressure; bpm, beats per minute; ECG, electrocardiogram; SD, standard deviation.

Supplementary Table 5. Liver volume at baseline and day 28 of treatment (Part A)

Time post-MMTT	Baseline		Day 28	
	Placebo (*n* = 9)	Cotadutide (*n* = 12)	Placebo (*n* = 9)	Cotadutide (*n* = 12)
**0h**				
Liver volume, l	2.23 (0.54)	2.02 (0.44)	2.18 (0.51)	1.71 (0.28)*
**4h**				
Liver volume, l	2.25 (0.60)	2.01 (0.40)	2.19 (0.55)	1.71 (0.27)*
**9h**				
Liver volume, l	2.29 (0.61)	2.04 (0.38)	2.21 (0.55)	1.74 (0.28)*
**14h**				
Liver volume, l	2.30 (0.58)	2.09 (0.42)	2.24 (0.53)	1.75 (0.24)*
**24h**				
Liver volume, l	2.15 (0.54)	1.97 (0.39)	2.12 (0.51)	1.70 (0.27)*

*Evaluable *n* = 10. Data are mean (SD). MMTT, mixed-meal tolerance test; SD, standard deviation.

An Exploratory Phase 2, Randomised, Double-blind, Placebo-controlled, and Open-label Active Comparator Study to Evaluate the Effect of MEDI0382 on Hepatic Glycogen Metabolism in Overweight and Obese Subjects with Type 2 Diabetes Mellitus

Sponsor Protocol Number: D5670C00022

Application Number: EudraCT number 2017-005081-22

Investigational Product: MEDI0382

Sponsor: [redacted]

Medical Monitor: [redacted]

Contract Research Organisation: Covance, Inc.

Protocol History, Date: Original Protocol, 29Jan2018
Protocol Amendment 1, 27Mar2018
Protocol Amendment 2, 29Mar2018
Protocol Amendment 3, 08May2018
Protocol Amendment 4, 24Sep2018
Protocol Amendment 5, 03May2019
Protocol Amendment 6, 15Jul2020

This document contains trade secrets and commercial information that is privileged or confidential and may not be disclosed without prior authorisation from MedImmune.

PROTOCOL SYNOPSIS

TITLE An Exploratory Phase 2, Randomised, Double-blind, Placebo-controlled, and Open-label Active Comparator Study to Evaluate the Effect of MEDI0382 on Hepatic Glycogen Metabolism in Overweight and Obese Subjects with Type 2 Diabetes Mellitus

HYPOTHESES

Primary Hypothesis:

Administration of MEDI0382 once daily subcutaneously (SC) titrated up to a dose level of 300 µg will result in a reduction in postprandial hepatic glycogen levels versus placebo after 28 days of treatment in overweight and obese subjects with type 2 diabetes mellitus (T2DM). (Part A only).

Administration of MEDI0382 once daily SC titrated up to a dose level of 300 µg will result in a reduction in fasting hepatic glycogen levels versus placebo after 35 days of treatment in overweight and obese subjects with T2DM (Part B only).

Secondary Hypothesis:

- Administration of MEDI0382 once daily SC titrated up to a dose level of 300 µg will result in a reduction in fasting hepatic glycogen levels versus liraglutide 1.8 mg administered SC once daily after 35 days of treatment in overweight and obese subjects with T2DM (Part B only).
- Administration of MEDI0382 once daily SC titrated up to a dose level of 300 µg once daily SC will result in a reduction in hepatic fat fraction versus placebo after 35 days of treatment in overweight and obese subjects with T2DM (Part B only).
- Administration of MEDI0382 once daily SC titrated up to a dose level of 300 µg will be well tolerated in overweight and obese subjects with T2DM.

OBJECTIVES AND ENDPOINTS

Objectives	Endpoints
**Primary**	
To assess the effect of MEDI0382 on hepatic glycogen levels versus placebo after 28 days (Part A) and 35 days (Part B) of treatment	• Change in hepatic glycogen concentration adjusted for liver volume as measured by magnetic resonance spectroscopy (MRS) at Time (T) = 4 hours post standardised morning meal from baseline (Day -1) to the end of 28 days of treatment (Part A)<br>• Percentage change in fasting hepatic glycogen concentration adjusted for liver volume as measured by magnetic resonance spectroscopy (MRS) at Time (T) = 24 hours post standardised morning meal from baseline (Day 1) to the end of 35 days of treatment (Day 36) (Part B)
**Secondary**	
To assess the effect of MEDI0382 on hepatic glycogen levels versus liraglutide after 35 days of treatment (Part B only)	Percentage change in fasting hepatic glycogen concentration adjusted for liver volume as measured by MRS at T = 24 hours post standardised morning meal from baseline (Day 1) to the end of 35 days of treatment (Day 36) (Part B only)
To assess the effect of MEDI0382 on hepatic fat fraction versus placebo after 35 days of treatment (Part B only)	Change in hepatic fat fraction from baseline as measured by magnetic resonance imaging (Day -1) to the end of 35 days of treatment (Part B only)

To evaluate the safety and tolerability of MEDI0382 titrated up to a dose level of 300 µg	Measures of safety and tolerability (vital signs, electrocardiograms [ECGs], laboratory test results, adverse events [AEs])
To characterise the immunogenicity profile of MEDI0382 exposure titrated up to a dose level of 300 µg	Development of anti-drug antibodies (ADA) and titre (if confirmed positive)
**Exploratory**	
[redacted]	[redacted]
[redacted]	[redacted]
[redacted]	[redacted]
[redacted]	[redacted]
[redacted]	[redacted]

[redacted]	[redacted]
[redacted]	[redacted]
[redacted]	[redacted]
[redacted]	[redacted]

STUDY DESIGN

This is a 2-part (Part A and Part B) exploratory Phase 2 study.

Part A is a randomised, double-blind, placebo-controlled study to evaluate the effect of MEDI0382 administered once daily SC for 28 days on hepatic glycogen metabolism in overweight and obese subjects with T2DM. Part A is planned to randomise up to 20 subjects. Subjects will be consented, screened for suitability, and randomised within 60 days if eligible. Subjects from Part A will not be re-enrolled in Part B.

Part B is an exploratory Phase 2 randomised, double-blind, placebo-controlled and an open-label active comparator study to evaluate the effect of MEDI0382 on hepatic glycogen metabolism in overweight and obese subjects with T2DM. Part B is planned to randomise approximately 30 subjects (not to exceed a maximum of 35 subjects). Subjects in Part B will be randomised to receive double-blind MEDI0382 [redacted] or placebo, or open-label liraglutide titrated from 0.6 to 1.8 mg once daily for 35 days.

The study will involve measurement of hepatic glycogen content using a carbon (C)-13 MRS based technique before and after completion of the treatment period. In Part A, MEDI0382 will be [redacted] in comparison to placebo. In Part B MEDI0382 will be titrated [redacted] and compared to placebo and liraglutide at a dose of 1.8 mg once daily titrated from 0.6 mg to 1.8 mg in 7-day intervals.

In Parts A and B subjects will undergo a 5-day washout period where metformin therapy will be suspended at the beginning of the study starting from Day -4 (metformin dosing to resume on Day 2). This washout is repeated at the end of the treatment period starting from Day 24 in Part A and Day 32 in Part B.

Across Part A of the study (up to 126 days in total including screening) subjects will have a total of 6 study visits, 6 nights of inpatient stay and will undergo a total of 10 MRS scans alongside additional assessments and blood sampling. In Part B, subjects will participate in the study for approximately 133 days and will have a total of 7 study visits (including 2 remote contacts), 6 nights of inpatient stay and will undergo a total of 8 MRS scans alongside additional assessments and blood sampling. The duration of MRS scans will be approximately 40 minutes with the exception of the baseline and end of treatment scans used for liver fat evaluation (Part B only) which will be prolonged and up to 1 hour 45 minutes in duration. Subjects will also be given the option to stay overnight prior to study visits if more convenient for them.

From Day -2 to Day 1 (and again on Day 8 to Day 15, Day 27 to Day 29 [Part A] and Day 34 to Day 36 [Part B] subjects will be asked to provide a stool sample for microbiome research purposes; this component of the study is optional.

On Day -3 in both parts of the study, subjects will be admitted to the clinical unit for 3 nights inpatient stay and will undergo initial safety assessments and receive training in SC injection administration. On Day -3 and Day -2, subjects will be provided with standardised solid meals (balanced with respect to nutrient content, but not calorie restricted) for breakfast, lunch, and evening meal and be expected to consume the entire meal and abstain from consumption of additional meals during this period. On the evening of Day -2, subjects will undergo a baseline blood test for ^{2}H-glucose prior to consuming deuterated water ($^{2}H_2O$) divided into two aliquots and separated by a 4-hour interval. Subjects will be expected to fast for 14 hours overnight (except for drinks of deuterated water).

On Day -1 subjects will undergo a baseline MRS scan to measure liver volume and glycogen (and liver fat in part B only) and will have blood samples collected for ^{2}H-glucose and baseline assessments prior to undergoing a standardised mixed-meal tolerance test (MMTT). The MMTT will consist of a liquid meal (400 mL Ensure Plus milkshake) (at Time [T] = 0). Blood will then be collected at specified time points to measure glucose levels. In Part A, at T = 4 hours after the MMTT a repeat MRS scan will be performed to measure liver volume and glycogen. For the remainder of the day serial MRS scans will be performed and blood samples will be collected at T = 9, and 14 hours and subjects will be given standardised solid meals for lunch and evening meal. In Part B, a repeat MRS scan to measure liver volume, fat and glycogen will be taken at T= 5 hours after the MMTT. A further MRS scan will be performed, and blood samples will be collected at T = 14 and subjects will be given standardised solid meals for lunch and evening meal.

On the morning of Day 1, following an overnight fast of at least 14 hours, subjects will have a final MRS scan performed (T = 24 hours) and blood sampling. On Day -1 or Day 1, eligibility criteria will be verified (the final eligibility check may occur at any time from Day -3 to Day 1) and subjects will be randomised to receive investigational product (MEDI0382 or placebo in Part A or MEDI0382, placebo, or liraglutide in Part B). Following predose safety measures, the subjects will receive their first dose of investigational product via SC injection and will be discharged from the clinical unit with a sufficient supply of investigational product to self-administer once daily by SC injection at home in the morning.

In Part A subjects will return for an outpatient visit at weekly intervals (Day 8 and Day 15) for safety assessments, dose up-titration until a dose of 300 µg MEDI0382 is reached in Part A. . Subjects will receive sufficient supply of investigational product at each outpatient visit.

On Day 26 subjects will be re-admitted to the clinical unit for a further 3 nights inpatient stay and as before will receive standardised solid meals during this period. On the evening of Day 27, subjects will fast and be given $^{2}H_2O$ water to drink at 2 times during the overnight period. On Day 28, following an MRS scan and blood sampling, subjects will receive an SC dose of investigational product and then undergo a MMTT as before at T = 0 and serial MRS scans and blood samples will be collected at T = 4, 9, and 14 hours. On Day 29, following an overnight fast, a final MRS scan will be performed alongside a blood test at T = 24 hours.

In Part B subjects will return for an outpatient visit on Day 8 for safety assessments, dose up-titration, and will receive an adequate supply of investigational product. On Days 14/15 and 21/22 remote contacts will be performed to advise on further dose uptitration as required and to collect AE/serious adverse events (SAEs) and concomitant medication information.

On Day 33 subjects will be re-admitted to the clinical unit for a further 3 nights inpatient stay and as before will receive standardised solid meals during this period. On the evening of Day 34, subjects will fast and be given $^{2}H_2O$ water to drink at 2 times during the overnight period. On Day 35, following an MRS scan and blood sampling, subjects will receive an SC dose of investigational product and then undergo a MMTT as

before at T = 0 and serial MRS scans and blood samples will be collected at T = 5 and 14 hours. On Day 36, following an overnight fast, a final MRS scan will be performed alongside a blood test at T = 24 hours.

A follow-up visit will be performed 28 days after the last dose of investigational product for safety assessments.

TARGET SUBJECT POPULATION

Subjects aged ≥ 18 years, with body mass index ≥ 27 and ≤ 40 kg/m^2, a diagnosis of T2DM on metformin monotherapy, and a glycated haemoglobin of ≤ 8.0% will be enrolled in the study. Females must not be pregnant and lactating females will be excluded. Females of childbearing potential should be using appropriate contraception.

TREATMENT GROUPS AND REGIMENS

Part A:

Following a screening period of up to 60 days, subjects will be randomised to receive either MEDI0382 or placebo once daily in the morning via SC injection as follows:

- MEDI0382 [redacted]
- Placebo for 28 days (N = 10)

Part B:

Following a screening period of up to 60 days, subjects will be randomised to receive either MEDI0382, placebo, or open-label liraglutide once daily in the morning via SC injection as follows:

- MEDI0382 [redacted]
- Open label liraglutide 0.6 mg for 7 days, followed by 1.2 mg for 7 days, followed by 1.8 mg for 21 days (N = 10)

STATISTICAL METHODS

Sample size:

In Part A the sample size of 8 completers in each MEDI0382 and placebo groups provides ~80% power to detect a difference of 19% reduction in the baseline glycogen concentration adjusted for liver volume (as measured by MRS at T = 4 hours post standardised liquid morning meal) after 28 days of treatment in the MEDI0382 versus placebo arms. This is under the assumption of baseline glycogen content of 283 µmol/mL and standard deviation of 41 µmol/mL for both groups and a two-sided significance level at 0.1 (Krssak et al, 2004; Rothman et al, 1991).

In Part B the sample size of 10 completers in each MEDI0382 and placebo groups provides > 80% power to detect a 24.2% reduction in fasting glycogen concentration adjusted for liver volume (as measured by MRS at T = 24 hours post standardised liquid morning meal) after 35 days of treatment in the MEDI0382 versus placebo arms. This is under the assumption of a common standard deviation of 17.0 % for both groups, a two-sided significance level at 0.05 and is based on the results of Part A.

Statistical analyses:

The efficacy analysis will be based on the intent-to-treat population, which is defined as all subjects that are randomised and received at least one dose of investigational product, and subjects will be analysed according to randomised treatment assignments. For the primary endpoint in Part A, change in glycogen concentration adjusted for liver volume as measured by MRS at T = 4 hours post standardised morning meal from baseline (Day -1) to the end of 28 days of treatment will be compared between the MEDI0382 and placebo groups using an analysis of covariance model adjusting for baseline value and treatment group. For Part B, the primary endpoint of percentage change in fasting glycogen concentration adjusted for liver volume measured by MRS at T = 24 hours post standardised morning meal from baseline (Day 1) to the end of 35 days of

treatment (Day 36) will be compared between MEDI0382 and placebo. A similar comparison between the MEDI0382 and liraglutide groups will be made as a secondary efficacy analysis.

Safety analyses:

The safety analyses will be based on the as-treated population. Treatment-emergent AEs and SAEs will be summarised by type, incidence, severity and relationship to investigational product by system organ class and preferred term. Other safety data, such as vital signs, clinical laboratory data, and electrocardiogram findings will be descriptively summarised at each time point. Change from baseline to each postbaseline time point in these data will also be summarised, where appropriate.

Pharmacokinetic analysis:

The MEDI0382 pharmacokinetic (PK) population includes all subjects who received at least one dose of MEDI0382 and had at least one PK blood sample taken that is above the lower limit of quantitation. Plasma C_{trough} concentrations of MEDI0382 will be summarised by dose level and by day in Part A and Part B separately.

Immunogenicity analysis:

The number and percentage of subjects with confirmed positive ADA against MEDI0382 and titre (if confirmed positive) will be reported.

Interim analysis:

An interim analysis will be performed after the last subject completes their last visit in Part A of the study.

TABLE OF CONTENTS

PROTOCOL SYNOPSIS 2
LIST OF ABBREVIATIONS 13
1 INTRODUCTION 15
1.1 Disease Background 15
1.2 MEDI0382 Background 15
1.3 Summary of Nonclinical Experience 15
1.4 Summary of Clinical Experience 16
1.5 Rationale for Conducting the Study 16
1.6 Benefit-Risk and Ethical Assessment 18
1.7 Research Hypotheses 18
1.7.1 Primary Hypothesis 18
1.7.2 Secondary Hypotheses 19
2 OBJECTIVES AND ENDPOINTS 20
2.1 Primary Objective and Associated Endpoint 20
2.2 Secondary Objectives and Associated Endpoints 20
2.3 Exploratory Objectives and Associated Endpoints 21
3 STUDY DESIGN 23
3.1 Description of the Study 23
3.1.1 Overview 23
3.1.2 Treatment 27
3.1.3 Dose Escalation 28
3.1.4 Management of Study Medication Related Toxicities 28
3.1.4.1 Tolerability 28
3.1.4.2 Hypoglycaemia 28
3.1.4.3 Hyperglycaemia 28
3.2 Rationale for Dose, Population, and Endpoints 29
3.2.1 Dose Rationale 29
3.2.2 Rationale for Study Population 30
3.2.3 Rationale for Endpoints 30
3.2.3.1 Primary Endpoint 30
3.2.3.2 Secondary Endpoints 31
3.2.3.3 Exploratory Endpoints 31
4 MATERIALS AND METHODS 32
4.1 Subjects 32
4.1.1 Number of Subjects 32
4.1.2 Inclusion Criteria 32
4.1.3 Exclusion Criteria 33
4.1.4 Subject Enrolment and Randomisation 35
4.1.5 Withdrawal from the Study 36

4.1.6 Discontinuation of Investigational Product 36
4.1.7 Replacement of Subjects 36
4.1.8 Withdrawal of Informed Consent for Data and Biological Samples 36
4.2 Schedule of Study Procedures 38
4.2.1 Enrolment/Screening Period 38
4.2.2 Randomised Treatment Period (Part A and Part B) 39
4.2.3 Follow-up Period 56
4.2.3.1 Early Discontinuation Visit or Unscheduled Study Visit 57
4.3 Description of Study Procedures 57
4.3.1 Efficacy 57
4.3.1.1 Mixed-meal Tolerance Tests 57
4.3.1.2 Magnetic Resonance Spectroscopy Scans 58
4.3.2 Safety Assessments 58
4.3.2.1 Medical History and Physical Examination 58
4.3.2.2 Assessment of the Injection Site 59
4.3.2.3 Electrocardiograms 59
4.3.2.4 Vital Signs 60
4.3.2.5 Weight, Height, and Body Mass Index Calculation 60
4.3.3 Clinical Laboratory Tests 60
4.3.3.1 Glucose Meter Measured Capillary Plasma Glucose Readings 61
4.3.4 Pharmacodynamic Evaluation and Measures 62
4.3.5 Pharmacokinetic Evaluation and Methods 62
4.3.6 Immunogenicity Evaluation and Methods 63
4.3.7 Biomarker Evaluation and Methods 63
4.3.8 Storage, Re-use, and Destruction of Biological Samples 63
4.3.9 Estimate of Volume of Blood to Be Collected 64
4.4 Study Suspension or Termination 64
4.5 Investigational Products 65
4.5.1 Identity of Investigational Product(s) 65
4.5.1.1 Investigational Product Handling 66
4.5.1.2 Investigational Product Inspection 67
4.5.1.3 Treatment Administration 68
4.5.1.4 Monitoring of Dose Administration 69
4.5.1.5 Reporting Product Complaints 69
4.5.2 Additional Study Medications 70
4.5.3 Labelling 70
4.5.4 Storage 70
4.5.5 Treatment Compliance 70
4.5.6 Accountability 70
4.6 Treatment Assignment and Blinding 71
4.6.1 Methods for Assigning Treatment Groups 71
4.6.2 Methods to Ensure Blinding 71
4.6.3 Methods for Unblinding 72
4.6.3.1 Unblinding in the Event of a Medical Emergency 72
4.7 Restrictions During the Study and Concomitant Treatment(s) 72

4.7.1 Permitted Concomitant Medications 72
4.7.2 Prohibited Concomitant Medications 72
4.8 Statistical Evaluation 73
4.8.1 General Considerations 73
4.8.1.1 Analysis Populations 73
4.8.2 Sample Size 74
4.8.3 Efficacy 74
4.8.3.1 Primary Efficacy Analysis 74
4.8.3.2 Secondary Efficacy Analyses 74
4.8.3.3 Exploratory Analyses 75
4.8.4 Safety 75
4.8.4.1 Analysis of Adverse Events 75
4.8.4.2 Analysis of Clinical Laboratory Parameters 75
4.8.5 Analysis of Pharmacokinetics 75
4.8.6 Analysis of Immunogenicity 75
4.8.7 Interim Analysis 75
5 ASSESSMENT OF SAFETY 76
5.1 Definition of Adverse Events 76
5.2 Definition of Serious Adverse Events 76
5.2.1 Hepatic Function Abnormalities 77
5.3 Recording of Adverse Events 77
5.3.1 Time Period for Collection of Adverse Events 77
5.3.2 Follow-up of Unresolved Adverse Events 77
5.4 Reporting of Serious Adverse Events 77
5.5 Other Events Requiring Immediate Reporting 78
5.5.1 Overdose 78
5.5.2 Hepatic Function Abnormality 79
5.5.3 Pregnancy 79
5.5.3.1 Maternal Exposure 79
6 STUDY AND DATA MANAGEMENT 80
6.1 Training of Study Site Personnel 80
6.2 Monitoring of the Study 80
6.2.1 Source Data 80
6.2.2 Study Agreements 80
6.2.3 Archiving of Study Documents 81
6.3 Study Timetable and End of Study 81
6.4 Data Management 81
6.5 Medical Monitor Coverage 81
7 ETHICAL AND REGULATORY REQUIREMENTS 82
7.1 Subject Data Protection 82
7.2 Ethics and Regulatory Review 82
7.3 Informed Consent 83

7.4 Changes to the Protocol and Informed Consent Form 83
7.5 Audits and Inspections 84
8 REFERENCES 85
9 CHANGES TO THE PROTOCOL 87
9.1 Protocol Amendment 6, 15Jul2020 87
9.2 Protocol Amendment 5, 03May2019 88
9.3 Protocol Amendment 4, 24Sep2018 89
9.4 Protocol Amendment 3, 08May2018 90
9.5 Protocol Amendment 2, 29Mar2018 91
9.6 Protocol Amendment 1, 27Mar2018 92
10 APPENDICES 93
10.1 Appendix 1 - Signatures 93
10.2 Appendix 2 – Contraception Guidance 94
10.3 Appendix 3 - Additional Safety Guidance 94
10.4 Appendix 4 - National Institute of Allergy and Infectious Disease and Food Allergy and Anaphylaxis Network Guidance for Anaphylaxis Diagnosis 98
10.5 Appendix 5 - Actions Required in Cases of Increases in Liver Biochemistry and Evaluation of Hy's Law 99
10.5.1 Introduction 99
10.5.2 Definitions 99
10.5.2.1 Potential Hy's Law 99
10.5.2.2 Hy's Law 99
10.5.3 Identification of Potential Hy's Law Cases 100
10.5.4 Follow-up 100
10.5.4.1 Potential Hy's Law Criteria Are Not Met 100
10.5.4.2 Potential Hy's Law Criteria Are Met 100
10.5.5 Review and Assessment of Potential Hy's Law Cases 101
10.5.6 Laboratory Tests 102
10.5.7 References 103
10.6 Appendix 6 - Genetic Research 104
10.7 Appendix 7 - Biological Samples 107
10.8 Appendix 8 – Example Timetable for In-clinic Standardised Meals, Deuterated Water, MMTTs, and Serial MRS Scans (Study Part A) 108

LIST OF TABLES

Table 1 Fasting Glucose and Liver Glycogen Content at Termination (Day 14) in DIO Mice, Group Means ± SEM 17
Table 2 Primary Objective and Associated Endpoint 20
Table 3 Secondary Objectives and Associated Endpoints 20

Table 4 Exploratory Objectives and Associated Endpoints 21
Table 5 Proposed Study Doses and Predicted Safety Margins Based on Cynomolgus Monkey Data 30
Table 6 Schedule of Screening Procedures 38
Table 7 Schedule of Treatment Period Study Procedures (Day -4 through Day 15) (Part A) 40
Table 8 Schedule of Treatment Period Study Procedures (Day 25 through Day 29) (Part A) 45
Table 9 Schedule of Treatment Period Study Procedures (Day -4 through Day 22) (Part B) 48
Table 10 Schedule of Treatment Period Study Procedures (Day 32 through Day 36) (Part B) 53
Table 11 Schedule of Follow-up Procedures 56
Table 12 Identification of Investigational Products 65
Table 13 Highly Effective Methods of Contraception 94
Table 14 Example Time Table for In-clinic Standardised Meals, Deuterated water, MMTTs, and Serial MRS Scans (Part A) 108

LIST OF IN-TEXT FIGURES

Figure 1 Study Flow Diagram Part A 26
Figure 2 Study Flow Diagram Part B 27

LIST OF ABBREVIATIONS

Abbreviation or Specialised Term	Definition
AE	adverse event
ADA	Anti-drug antibody/antibodies
ALT	alanine transaminase
ANCOVA	analysis of covariance
AST	aspartate transaminase
AUC	area under the concentration-time curve
BMI	body mass index
BP	blood pressure
C	carbon
C2	carbon 2
C5	carbon 5
C6	carbon 6
CI	confidence interval
C_{max}	maximal plasma concentration
COVID-19	coronavirus disease
C_{trough}	Trough plasma concentration, measured at the end of a dosing interval taken directly before next administration
ECG	electrocardiogram
eCRF	electronic case report form
EDC	electronic data capture
eGFR	estimated glomerular filtration rate
GI	gastrointestinal
^2H	deuterium
^2H_2O	deuterated (heavy) water
HbA1c	glycated haemoglobin
GLP-1	glucagon-like peptide-1
HBsAg	hepatitis B surface antigen
HIV	human immunodeficiency virus
ICF	informed consent form
ICH	International Council on Harmonisation
IEC	Independent Ethics Committee
IFU	instructions for use

Abbreviation or Specialised Term	Definition
ITT	intent-to-treat
IXRS	interactive voice/web response system
MedDRA	medical dictionary for regulatory activities
MMTT	mixed-meal tolerance test
MRI	Magnetic resonance imaging
MRS	magnetic resonance spectroscopy
NASH	non-alcoholic steatohepatitis
NOAEL	no-observed-adverse-effect level
PK	pharmacokinetic(s)
SAE	serious adverse event
SC	subcutaneous
SID	subject identification
SOC	system organ class
T	Time
T2DM	type 2 diabetes mellitus
ULN	upper limit of normal

1 INTRODUCTION

1.1 Disease Background

The rising prevalence of type 2 diabetes mellitus (T2DM) and obesity is a cause of substantial health and economic burden worldwide. In many cases of T2DM, significant weight loss (typically 5% of body weight or more) can promote improvements in glycaemic control, cardiovascular risk, and mortality rates, and may even slow or reverse disease progression (Petersen et al, 2005). Many existing therapies for T2DM focus upon lowering blood glucose; however, there is a major unmet need for treatments that both improve glycaemic control and achieve disease-modifying weight loss.

1.2 MEDI0382 Background

MEDI0382 is a synthetic peptide with both glucagon-like peptide-1 (GLP-1) and glucagon receptor agonist activity which is under development for the treatment of T2DM and non-alcoholic steatohepatitis (NASH). GLP-1 receptor agonists are established treatments for T2DM that improve glycaemic control, delay gastric emptying, and depress appetite leading to modest, but often non-sustained weight loss (typically 3% versus baseline at one year). Glucagon has similar effects to GLP-1 on gastric emptying and appetite, and has also been shown to promote increased energy expenditure (Lynch et al, 2014; Habegger et al, 2013). Oxyntomodulin, a naturally occurring peptide with GLP-1 and glucagon receptor co-agonist activity, has been shown to promote weight loss through effects on appetite and energy expenditure (Wynne et al, 2006) and co-infusion of GLP-1 and glucagon has synergistic effects on reducing food intake and promoting weight loss in human subjects (Bagger et al, 2015).

1.3 Summary of Nonclinical Experience

[redacted]

[redacted]

[redacted]

1.4 Summary of Clinical Experience

Prior clinical studies with MEDI0382 have included a single ascending dose study (D5670C00001) in healthy subjects, and a multiple ascending dose and a Phase 2a study (D5670C00002) conducted in obese and overweight subjects with T2DM. In the Phase 2a study (Cohort 4 of Study D5670C00002), treatment with MEDI0382 at 200 µg (uptitrated from a starting dose of 100 µg for 41 days with 2 titration steps of 4 days duration) was associated with a mean reduction from baseline in body weight of 4.1% (90% confidence interval [CI] -4.5, -3.4) in MEDI0382-treated subjects versus 1.8% (90% CI -2.5, -1.0) in placebo, $p < 0.001$, and a mean reduction from baseline in glucose $AUC_{MMT.4h}$ of 32.8% (90% CI -37.0, -28.6) vs 10.2% (90% CI -14.1, -6.2) in placebo, $p \leq 0.0001$. While a reduction in blood glucose area under the concentration-time curve (AUC) is also anticipated in this study, given the difference in baseline subject characteristics (eg, metformin washout) and the higher carbohydrate content of the mixed-meal tolerance test (MMTT) assessment in this study, it is expected that the magnitude of AUC glucose reduction will be different.

MEDI0382 was also shown to have an acceptable safety profile in Study D5670C00002. There were no unexpected safety signals and no increases in systolic or diastolic BP across the study. In Cohort 4, dosing over 41 days was associated with increased gastrointestinal (GI) adverse events (AEs) with 40 AEs of vomiting observed in 8 (32%) subjects; however, the number of events diminished in frequency with up-titration (N = 19 at 100 µg, N = 10 at 150 µg, and N = 11 at 200 µg). The rates of nausea and vomiting were further reduced by lengthening the interval between titration steps to 5 days in Cohorts 5 and 6 of the same study dosing up to a dose level of 300 µg.

Refer to the current MEDI0382 IB for a complete summary of clinical information.

1.5 Rationale for Conducting the Study

MEDI0382, a GLP-1 receptor and glucagon receptor co-agonist is under development for the treatment of T2DM and NASH. Clinical studies to date have shown significant reductions in blood glucose levels in treated subjects, despite the known action of glucagon receptor agonists to increase blood glucose levels. The study of glucose and glycogen metabolism is therefore important to gain an increased understanding of the mechanism of action of MEDI0382.

Glucagon primarily raises blood glucose levels via increased hepatic glucose output. This is initially achieved via an increase in glycogenolysis, and later enhanced gluconeogenesis (Cherrington et al, 1981). In contrast, GLP-1 promotes glucose-dependent insulin release which both suppresses gluconeogenesis and promotes glycogen synthesis and hepatic glucose uptake. However, the relative contribution of each of these counter-regulatory mechanisms to hepatic glucose and glycogen metabolism in the setting of dual glucagon and GLP-1 receptor agonism is at present unclear. Furthermore, an added level of complexity is that subjects with T2DM exhibit dysregulated hepatic glucose production in the fasted state, with an imbalance towards heightened gluconeogenesis over glycogenolysis in contrast to healthy subjects (Savage et al, 2007). Finally, as hepatic expression of the glucagon receptor is far greater than that of the GLP-1 receptor, results obtained in this study may provide evidence of target engagement of MEDI0382 with the glucagon receptor, and could serve to differentiate MEDI0382 from GLP-1 receptor mono-agonists (Uhlén et al, 2015).

[redacted]

Table 1 Fasting Glucose and Liver Glycogen Content at Termination (Day 14) in DIO Mice, Group Means ± SEM

[redacted]	[redacted]	[redacted]	[redacted]
[redacted]	[redacted]	[redacted]	[redacted]
[redacted]	[redacted]	[redacted]	[redacted]
[redacted]	[redacted]	[redacted]	[redacted]

DIO = diet-induced obesity; eqv = equivalent; SEM = standard error of the mean
*p < 0.05; **p < 0.01; ***p < 0.001; ****p < 0.0001
N = 6 female mice/group
Source: Study AZM110628-01-18

In prior clinical studies in overweight and obese subjects with T2DM dosed with MEDI0382 for 41 days, marked reductions in both fasting and postprandial blood glucose levels were observed; however, measures of hepatic glycogen content were not performed.

The main aim of this study is to establish if the glucagon-receptor agonist component of MEDI0382 has a measurable impact on hepatic glycogen levels in subjects with T2DM across a 24-hour period in comparison to placebo in Part A, and to placebo and liraglutide (a GLP-1 receptor mono agonist) in Part B. This study will also determine if MEDI0382 modulates the ratio of hepatic gluconeogenesis to glycogenolysis via incorporation of

deuterium (^{2}H) into glucose at a different position in the molecule across a 24-hour period, and to ascertain if this differs with respect to placebo in Part A, and to placebo and liraglutide in Part B.

1.6 Benefit-Risk and Ethical Assessment

The study will be performed in accordance with ethical principles that have their origin in the Declaration of Helsinki and are consistent with International Council on Harmonisation (ICH)/Good Clinical Practice, and applicable regulatory requirements.

MEDI0382 is a GLP-1 receptor and glucagon receptor co-agonist that promotes glucose lowering and weight loss and is targeted at subjects with T2DM. MEDI0382 has the potential to deliver improvements in glycaemic control and lipid homeostasis, and is anticipated to be a useful therapy for T2DM. However, it should be noted that given the short treatment duration in this study little direct benefit to the patient's underlying T2DM should be expected.

Nausea and vomiting events have been observed in other clinical studies with MEDI0382. The study protocol includes strategies to manage gastrointestinal AEs if they arise, including reduction of meal sizes and maintaining adequate hydration. As clinical data for MEDI0382 is still limited, potential risks for MEDI0382 are based on published data for GLP-1 receptor agonists and glucagon receptor co-agonists in addition to available nonclinical data and clinical data for MEDI0382. According to the latest version of the IB, potential risks for MEDI0382 include an increase in heart rate, alterations in blood pressure, QT interval prolongation, anaphylactic-type reactions, injection site reactions, skin rash, pancreatitis, pancreatic carcinoma, and thyroid cancer.

The study design aims to minimise potential risks to subjects participating in this study based on the proposed inclusion/exclusion criteria, safety monitoring, and up-titration dosing schedule. All subjects will be monitored throughout the study to ensure adequate glycaemic control. Subjects will be given appropriate training in SC injection administration as well as use of any devices. Subjects with contraindications to magnetic resonance imaging (MRI) scanning are excluded from the study.

Refer to Section 1.4 and the current IB for further information on the potential benefits of MEDI0382 and an assessment of the potential and known risks.

1.7 Research Hypotheses

1.7.1 Primary Hypothesis

- Administration of MEDI0382 once daily SC titrated up to a dose level of [redacted] will result in a reduction in postprandial hepatic glycogen levels versus placebo after 28 days of treatment in overweight and obese subjects with T2DM (Part A only).

- Administration of MEDI0382 once daily SC titrated up to a dose level of [redacted] will result in a reduction in fasting hepatic glycogen levels versus placebo after 35 days of treatment in overweight and obese subjects with T2DM (Part B only).

1.7.2 Secondary Hypotheses

- Administration of MEDI0382 once daily SC titrated up to a dose level of [redacted] will result in a reduction in fasting hepatic glycogen levels versus liraglutide 1.8 mg once daily SC after 35 days of treatment in overweight and obese subjects with T2DM (Part B only).
- Administration of MEDI0382 once daily SC titrated up to a dose level of [redacted] once daily SC will result in a reduction in hepatic fat fraction versus placebo after 35 days of treatment in overweight and obese subjects with T2DM (Part B only).
- Administration of MEDI0382 once daily SC titrated up to a dose level of [redacted] will be well tolerated in overweight and obese subjects with T2DM.

2 OBJECTIVES AND ENDPOINTS

2.1 Primary Objective and Associated Endpoint

Table 2 Primary Objective and Associated Endpoint

Type	Objective	Endpoint
Efficacy	To assess the effect of MEDI0382 on hepatic glycogen levels versus placebo after 28 days (Part A) and 35 days (Part B) of treatment	• Change in hepatic glycogen concentration adjusted for liver volume as measured by MRS at T = 4 hours post standardised morning meal from baseline (Day -1) to the end of 28 days of treatment (Part A only)<br>• Percentage change in fasting hepatic glycogen concentration adjusted for liver volume as measured by MRS at T = 24 hours post standardised morning meal from baseline (Day 1) to the end of 35 days of treatment (Day 36) (Part B)

MRS= magnetic resonance spectroscopy; T = time.

2.2 Secondary Objectives and Associated Endpoints

Table 3 Secondary Objectives and Associated Endpoints

Type	Objective	Endpoint
Efficacy	To assess the effect of MEDI0382 on hepatic glycogen levels versus liraglutide after 35 days of treatment (Part B only)	Percentage change in fasting hepatic glycogen concentration adjusted for liver volume as measured by MRS at T = 24 hours post standardised morning meal from baseline (Day 1) to the end of 35 days of treatment (Day 36, Part B only)
Efficacy	To assess the effect of MEDI0382 on hepatic fat fraction versus placebo after 35 days of treatment (Part B only)	Change in hepatic fat fraction from baseline as measured by magnetic resonance imaging (Day -1) to the end of 35 days of treatment (Part B only)
Safety	To evaluate the safety and tolerability of MEDI0382 titrated up to a dose level of 300 μg	Measures of safety and tolerability (vital signs, ECGs, laboratory test results, AEs)
Immunogenicity	To characterise the immunogenicity profile of MEDI0382 titrated up to a dose level of 300 μg	Development of ADA and titre (if confirmed positive)

AE = adverse event; ADA = anti-drug antibodies; ECG = electrocardiogram; MRS = magnetic resonance spectroscopy; T = time.

2.3 Exploratory Objectives and Associated Endpoints

Table 4 Exploratory Objectives and Associated Endpoints

Type	Objective	Endpoint
Efficacy	[redacted]	[redacted]
[redacted]	[redacted]	[redacted]
[redacted]	[redacted]	[redacted]
[redacted]	[redacted]	[redacted]
[redacted]	[redacted]	[redacted]

Table 4 Exploratory Objectives and Associated Endpoints

Type	Objective	Endpoint
		[redacted]
[redacted]	[redacted]	[redacted]
[redacted]	[redacted]	[redacted]
[redacted]	[redacted]	[redacted]
[redacted]	[redacted]	[redacted]

[redacted]

3 STUDY DESIGN

3.1 Description of the Study

3.1.1 Overview

This is a 2-part (Part A and Part B) exploratory Phase 2 study.

Part A is a randomised, double-blind, placebo-controlled study to evaluate the effect of MEDI0382 administered once daily SC for 28 days on hepatic glycogen metabolism in overweight and obese subjects with T2DM. Part A is planned to randomise up to 20 subjects (Figure 1). Subjects will be consented, screened for suitability, and randomised within 60 days if eligible. Subjects from Part A will not be re-enrolled in Part B.

Part B is an exploratory Phase 2 randomised, double-blind, placebo-controlled and open-label active comparator study to evaluate the effect of MEDI0382 on hepatic glycogen metabolism in overweight and obese subjects with T2DM (Figure 2). In Part B it is planned to randomise approximately 30 subjects (not to exceed a maximum of 35 subjects). Subjects in Part B will be randomised to receive double-blind MEDI0382 [redacted] or placebo, or open-label liraglutide titrated from 0.6 to 1.8 mg once daily for 35 days.

Part A:
Subjects will undergo a 5-day washout period where metformin therapy will be suspended at the beginning of the study starting from Day -4 (metformin dosing to resume on Day 2) and the end of the study starting from Day 25 (metformin dosing to resume on Day 30). Across the course of the study (up to 126 days in total including screening) subjects will have a total of 6 study visits, 6 nights of inpatient stay and will undergo a total of 10 MRS scans alongside additional assessments and blood sampling. Subjects will be given the option to stay overnight prior to study visits if more convenient for them. From Day -2 to Day 1 (and again on Day 8 to Day 15 and Day 27 to Day 29) [redacted]

On Day -3 subjects will be admitted to the clinical unit for 3 nights of inpatient stay and will undergo initial safety assessments and receive training in SC injection administration. On Day -3 and Day -2, subjects will be provided with standardised solid meals (balanced with respect to nutrient content, but not calorie restricted) for breakfast, lunch, and evening meal and be expected to consume the entire meal and abstain from consumption of additional meals during this period.

On the evening of Day -2, subjects will undergo a baseline blood test for ^{2}H-glucose prior to consuming deuterated water ($^{2}H_2O$) divided into two aliquots and separated by a 4-hour interval. Subjects will be expected to fast for 14 hours overnight (except for drinks of $^{2}H_2O$). The volume of each drink of $^{2}H_2O$ will be 2.3 mL per kg of body water (body water is

calculated as 50% of total body weight for women and 60% of total body weight for men) and should ideally be consumed within 15 minutes.

On Day -1, subjects will undergo a baseline MRS scan to measure liver volume, hepatic glycogen, and fat fraction (the latter in Part B only). After the MRS scan, subjects will undergo a standardised mixed-meal tolerance test (MMTT). Prior to consumption of a liquid meal (400 ml Ensure Plus milkshake) while subjects are still fasted, blood samples will be collected for baseline assessments including ^{2}H enriched glucose and non-enriched glucose, energy-related and gluconeogenic substrates, pharmacokinetic (PK), and nongenetic future research. Following these blood sample collections, the Ensure Plus milkshake will be administered (at Time [T] = 0). Blood samples will then be collected at specified time points to measure glucose levels and at T = 220 minutes, additional blood samples for ^{2}H labelled glucose and nongenetic samples will be collected at the same time. After the MMTT at T = 4 hours, a repeat MRS scan will be performed. For the remainder of the day serial MRS scans and blood sample collections for ^{2}H labelled glucose and non-genetic research will be performed at T = 9 and 14 hours and subjects will be given standardised solid meals for lunch and evening meal.

On the morning of Day 1, following an overnight fast of at least 14 hours, subjects will have a final MRS scan and blood sampling performed (T = 24 hours). On Day -1 or Day 1, eligibility criteria will be verified (the final eligibility check may occur at any time from Day -3 to Day 1) and subjects will be randomised to receive investigational product (MEDI0382 or placebo). Following predose safety measures after the final MRS scan, the subjects will receive the first dose of investigational product via SC injection and be discharged from the clinical unit with sufficient supply of investigational product to self-administer once daily by SC injection at home in the morning.

Subjects will return for an outpatient visit at weekly intervals (Day 8 and Day 15) for safety assessments, dose up-titration until a dose of 300 µg. Sufficient drug supply for home dosing will be provided at each visit.

On Day 26 subjects will be re-admitted to the clinical unit for a further 3 nights inpatient stay and as before will receive standardised solid meals during this period. On the evening of Day 27, subjects will fast and be given $^{2}H_2O$ to drink at 2 times during the overnight period. On Day 28, following an MRS scan and blood sampling, subjects will be administered an SC dose of investigational product and then undergo an MMTT as before at T = 0 with serial blood samples collected as before and MRS scans performed at T = 9 and 14 hours.

On Day 29, following an overnight fast, a final MRS scan and blood sampling will be performed at T = 24 hours. A follow up visit will be performed 28 days after the last dose of investigational product for safety assessments.

Part B:

In Part B a comparison will be made between MEDI0382, [redacted], placebo and liraglutide 1.8 mg once daily titrated from 0.6 to 1.8 mg in 7-day intervals. The treatment duration will be 35 days in each treatment group, with subjects randomised to liraglutide spending 21 days at 1.8 mg, in contrast subjects randomised to MEDI0382 will spend 14 days at the top dose of 300 µg.

The procedures for subjects who go through to the completion of the visit on Day 8, are identical to Part A; aside from the MRS scanning and related activities that will occur at baseline prior to treatment, then at T = 0, 5, 14, and 24 hours and at the end of the 35 day treatment period as detailed in the schedule of events. The duration of MRS scans will be approximately 40 minutes with the exception of the baseline and end of treatment scans used for liver fat evaluation which will be prolonged and up to 1 hour 45 minutes in duration. Subjects will undergo a 5-day washout period where metformin therapy will be suspended at the beginning of the study starting from Day 4 (metformin dosing to resume on Day 2) and the end of the study starting from Day 32 (metformin dosing to resume on Day 37).

Subjects will leave the clinical unit on Day 8 with sufficient investigational product to dose themselves every day until returning to the clinical unit on Day 33. Site staff will make remote contact with subjects on Days 14 or 15 and 21 or 22 to remind them to make an uptitration step if required per randomisation and to collect information regarding adverse events and concomitant medications. Contact may be made the day prior to the titration step being made to serve as a reminder, ideally by telephone, but any means of contact where confirmation of information receipt is acceptable and should be made prior to the time that the dose is scheduled on Days 15 and 22.

On Day 33 subjects will be re-admitted to the clinical unit for a further 3 night inpatient stay and as before will receive standardised solid meals during this period. On the evening of Day 34, subjects will fast and be given $^{2}H_2O$ to drink at 2 timepoints during the overnight period. On Day 35, following an MRS scan and blood sampling, subjects will administer their final SC dose of investigational product and then undergo an MMTT as before at T = 0 with serial blood samples collected as before and MRS scans performed at T = 5 and 14 hours.

On Day 36, following an overnight fast, a final MRS scan and blood sampling will be performed at T = 24 hours. A follow up visit will be performed 28 days after the last dose of investigational product for safety assessments.

Figure 1 Study Flow Diagram Part A

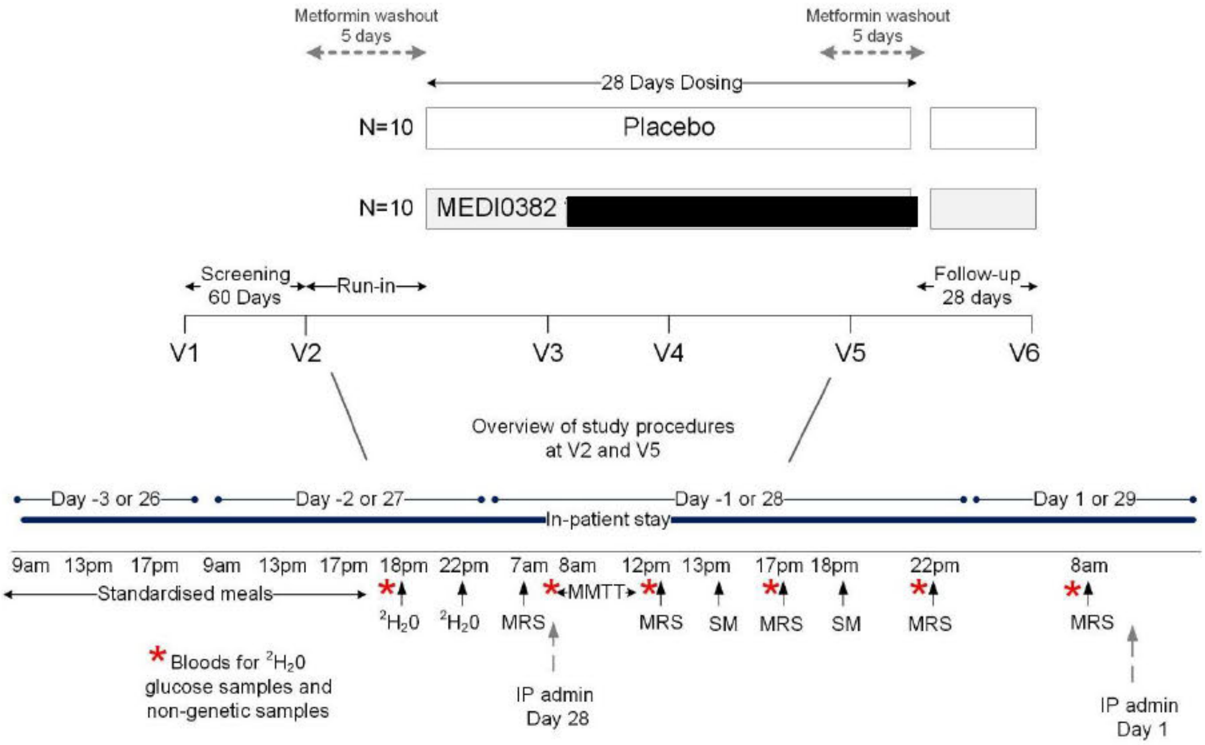

^2H_2O = deuterated water (administration); IP = investigational product; MMTT = mixed-meal tolerance test; MRS = magnetic resonance spectroscopy; SM = standardised meal.
Note that timings may change according to schedule – see schedule of procedures (Table 6, Table 7, and Table 8) and in an example timetable provided in Section 10.8.

Figure 2 Study Flow Diagram Part B

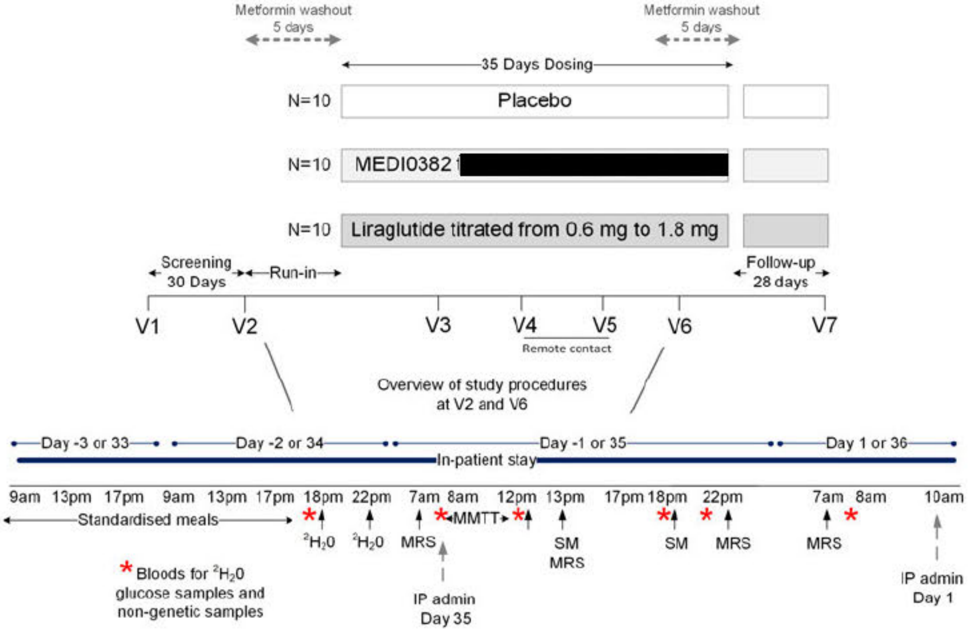

^2H_2O = deuterated water (administration); IP = investigational product MMTT = mixed-meal tolerance test; MRS = magnetic resonance spectroscopy; SM = standardised meal.
Note that timings may change according to schedule of study procedures (Table 6, Table 7, Table 8, Table 9, Table 10, and Table 11) and in an example timetable provided in Section 10.8.

The endpoints to be measured in this study are described in Section 2.

3.1.2 Treatment

Part A

Subjects will be randomised using a ratio of 1:1 to receive either MEDI0382 titrated from [redacted] or placebo. A sufficient number of subjects will be invited to participate in the study such that 8 subjects will complete dosing in the MEDI0382 arm and 8 subjects will complete dosing in the placebo arm.

Part B

Subjects will be randomised using a ratio of 1:1:1 to receive either MEDI0382 [redacted], liraglutide titrated in 7-day intervals from 0.6 mg up to 1.8 mg, or placebo, respectively. A sufficient number of subjects will be invited to participate in the study such that approximately 10 subjects will complete dosing in each treatment arm (MEDI0382, liraglutide, and placebo). Approximately 30 subjects (maximum of 35) will be enrolled into Part B of the study.

Approximately 50 subjects will participate in the study (Part A and Part B).

3.1.3 Dose Escalation

Part A: Doses will escalate from an initial dose of [redacted] for the MEDI0382 and placebo arms. Dose reduction is not permitted in this study.

Part B: Doses will escalate from an initial dose of [redacted] for the MEDI0382 and placebo arms. Doses will escalate from an initial dose of 0.6 to 1.8 mg in 7-day intervals for the liraglutide arm. Dose reduction is not permitted in this study.

3.1.4 Management of Study Medication Related Toxicities

3.1.4.1 Tolerability

If a subject experiences nausea and vomiting in relation to investigational product, in the first instance, conservative measures should be advised including reducing meal size and maintaining adequate hydration. Where necessary, if there is persistent vomiting, a subject may be given an antiemetic to control his/her symptoms; a 5HT3 receptor antagonist (eg, ondansetron) or cyclizine is preferable in this situation, rather than antiemetics which may affect gastric emptying, and in particular dopamine receptor antagonists (eg, metoclopramide or domperidone).

3.1.4.2 Hypoglycaemia

A hypoglycaemic event is considered severe if associated with severe cognitive impairment requiring external assistance for recovery, as defined by the American Diabetes Association. Spontaneous and clinically significant hypoglycaemia defined as blood glucose < 3.0 mmol/L or 54 mg/dL with or without symptoms (Skyder et al, 2017) has not been experienced in prior studies with MEDI0382 up to a dose of 300 µg alongside metformin treatment and is uncommon with liraglutide and metformin co-administration. Clinically significant hypoglycaemia is therefore unlikely to occur in this study. All subjects will be provided with a diary and a glucose meter and will be advised to check their capillary plasma glucose level if they have symptoms of hypoglycaemia (hunger, dizziness, shaking, sweating, or irritability) or feel unwell and will be expected to record the level in their diary. Local protocols for treatment and follow-up of any hypoglycaemic episode should be followed. Any blood glucose level of < 3.0 mmol/L (54 mg/dL) is considered to be clinically significant hypoglycaemia and should be reported by investigators as an AE, regardless of whether subjects have symptoms or not. Pharmacological treatments administered for hypoglycaemia, eg, dextrose/glucose tablets, glucagon etc, should be recorded in the electronic case report form (eCRF) as concomitant medications.

3.1.4.3 Hyperglycaemia

In the event of suspected persistent hyperglycaemia in a subject based on either symptoms of hyperglycaemia (eg, thirst, polyuria, blurred vision) or capillary plasma glucose readings (eg, 3 readings > 260 mg/dL [14.4 mmol/L] within one week), the investigator should perform additional fasting blood glucose levels as necessary. If 2 or more laboratory plasma fasting

glucose levels of > 260 mg/dL [14.4 mmol/L] more than 3 days apart are detected, the investigator should consider rescue therapy following discussion with the medical monitor.

3.2 Rationale for Dose, Population, and Endpoints

3.2.1 Dose Rationale

PK information collected in previous single and multiple dose clinical studies (Studies D5670C00001 and D5670C00002) suggest that MEDI0382 has a linear profile in the dose range 5 to 300 µg with a half-life of approximately 8 to 13 hours, which allows minimal accumulation after repeat daily administration and achievement of steady state between 4 and 7 days. Based on PK/pharmacodynamic (PD) modelling conducted using available clinical data and from a literature review of GLP-1 receptor and glucagon receptor modulators, the maximal clinically efficacious dose for glucose control of MEDI0382 was predicted to be in the range of 50 to 300 µg/day.

[redacted]

[redacted]

[redacted]

[redacted]

Table 5 [redacted]

[redacted]	[redacted]	[redacted]

[redacted]

3.2.2 Rationale for Study Population

Prior studies in subjects with T2DM have revealed differences in glucose and glycogen homeostasis in comparison to healthy subjects (Savage et al, 2007). For this reason, subjects who are overweight or obese with T2DM will be studied to ensure that any results or insights gained are relevant and applicable to the future intended treatment population. As sustained hyperglycaemia and poor glycaemic control may influence hepatic glycogen levels, subjects with likely early stage T2DM on metformin monotherapy in the absence of poor glycaemic control will be recruited to minimise variability in the primary endpoint.

In addition, metformin will be washed out prior to measurement of the primary endpoint as metformin therapy leads to suppression of gluconeogenesis and may therefore indirectly influence glycogen turnover.

3.2.3 Rationale for Endpoints

3.2.3.1 Primary Endpoint

The primary endpoint of change in hepatic glycogen adjusted for liver volume 4 hours (Part A) after a standardised meal has been chosen as this corresponds to the time at which the greatest effect of MEDI0382 on glycogen levels would be predicted to occur. Studies in subjects evaluating hepatic glycogen stores (in some cases using a C-13 labelled glucose) have shown that peak glycogen levels are observed at 4 to 5 hours post-ingestion of a meal (Krssak et al, 2004, Moore et al, 2017, Magnusson et al, 1992), and this timing coincides with the time of maximal plasma concentration of MEDI0382 at approximately 4 hours.

The primary endpoint of % change in fasting glycogen adjusted for liver volume has been selected for Part B as a statistically significant reduction in fasting glycogen (at both T = 0 and T = 24 hours) was observed in Part A and variability at these time-points was lower than other time-points permitting a smaller sample size. The T = 24 hour fasting time-point has been selected rather than the T = 0 hour time point to perform the analysis as this is optimal from an operational perspective.

3.2.3.2 Secondary Endpoints

Hepatic Glycogen Concentrations

Estimations of hepatic glycogen concentration will be measured by MRS and adjusted for liver volume at multiple time points across the study to establish the effects of MEDI0382 on glycogen in the fasted and postprandial state, and at different time points in the day.

Safety, Tolerability, and Immunogenicity

As MEDI0382 remains under development in Phase 2 with limited exposure to date; safety, tolerability, and immunogenicity will be assessed throughout this study.

3.2.3.3 [redacted]

[redacted]

[redacted]

[redacted]

[redacted]

[redacted]

Measures of MEDI0382 Exposure and Immunogenicity

Trough plasma concentrations (C_{trough}) of MEDI0382 at 200 and 300 µg in Part A will be measured at the end of a dosing interval to check that subjects have been exposed to the drug.

Anti-drug antibody (ADA) incidence rate and titre will be tabulated for each treatment to monitor immunogenicity. Tiered analyses will be performed to include screening, confirmatory, and titre assay components; samples confirmed positive for ADA will be tested and analysed for antibody titre and may be utilised for further characterisation of the ADA response.

4 MATERIALS AND METHODS

4.1 Subjects

4.1.1 Number of Subjects

Part A**:** Enrolment of up to 20 subjects across one or more study sites is planned.

Part B**:** Enrolment of approximately 30 subjects (maximum 35 subjects) across three or more study sites is planned.

Overall, approximately 50 subjects will be enrolled in the study (Part A and Part B, inclusive).

4.1.2 Inclusion Criteria

Subjects must meet all the following criteria:

1 Male and female subjects aged ≥ 18 years at screening

2 Provision of signed and dated written informed consent (except for consent for genetic research and stool sample microbiome research) prior to any study-specific procedures

3 Body mass index (BMI) ≥ 27 and ≤ 40 kg/m^2 (inclusive) at screening

4 Glycated haemoglobin (HbA1c) ≤ 8.0% at screening*

5 Diagnosed with T2DM with glucose control managed with metformin monotherapy where no significant dose change (increase or decrease ≥ 500 mg/day) has occurred in the 3 months prior to screening

6 Female subjects of childbearing potential must have a negative pregnancy test at screening and randomisation, and must not be lactating

7 Female subjects of childbearing potential who are sexually active with a non-sterilised male partner must be using at least one highly effective method of contraception from screening and up to 4 weeks after the last dose of investigational product. A highly effective method of contraception is defined as one that results in a low failure rate (ie, less than 1% per year) when used consistently and correctly (see Section 10.2 for definition of females of childbearing potential and for a description of highly effective methods of contraception).

*Subjects may be re-tested for the HbA1c entry criterion only once.

4.1.3 Exclusion Criteria

Any of the following would exclude the subject from participation in the study:

1 History of, or any existing condition that, in the opinion of the investigator, would interfere with evaluation of the investigational product, put the subject at risk, influence the subject's ability to participate or affect the interpretation of the results of the study and/or any subject unable or unwilling to follow study procedures. Specific examples include: dislike/unable to eat any of the standardised meals that will be used during the study and poor venous access.

2 Any subject who has received another investigational product as part of a clinical study or a GLP-1 analogue-containing preparation within the last 30 days or 5 half-lives of the drug (whichever is longer) at the time of screening

3 Any subject who has received any of the following medications within the specified time frame prior to the start of the study as detailed in Section 4.7.2 .

- Herbal preparations or drugs licensed for control of body weight or appetite (eg, orlistat, bupropion naltrexone, phentermine-topiramate, phentermine, lorcaserin)
- Opiates, domperidone, metoclopramide or other drugs known to alter gastric emptying
- Glucagon

   - Warfarin

4. Concurrent participation in another study with an investigational product and repeat randomisation in this study is prohibited; subjects randomised into Part A of the study may not be randomised into Part B of the study
5. Severe allergy/hypersensitivity to any of the proposed study treatments, excipients, C-13 labelled glucose, deuterated water (^2H_2O), or ingredients of standardised meals
6. Any contraindication to magnetic resonance imaging/MRS scanning including claustrophobia or dislike of confined spaces
7. Symptoms of acutely decompensated blood glucose control (eg, thirst, polyuria, weight loss), a history of type 1 diabetes mellitus (T1DM) or diabetic ketoacidosis, or if the subject has been treated with daily SC insulin within 90 days prior to screening
8. Recurrent unexplained hypoglycaemic episodes (defined as glucose < 3.0 mmol/L or < 54 mg/dL on more than 2 occasions in 6 months prior to screening)
9. Significant inflammatory bowel disease, gastroparesis, or other severe disease or surgery affecting the upper GI tract (including weight-reducing surgery and procedures) which may affect gastric emptying or could affect the interpretation of safety and tolerability data
10. Acute or chronic pancreatitis
11. Significant hepatic disease (except for NASH or nonalcoholic fatty liver disease without portal hypertension or cirrhosis) and/or subjects with any of the following results at screening:
    - Aspartate transaminase (AST) ≥ 3 × upper limit of normal (ULN)
    - Alanine transaminase (ALT) ≥ 3 × ULN
    - Total bilirubin ≥ 2 × ULN
12. Impaired renal function defined as estimated glomerular filtration rate (eGFR) < 30 mL/minute/1.73m^2 at screening (glomerular filtration rate estimated according to Modification of Diet in Renal Disease (MDRD) using MDRD Study Equation IDMS-traceable (International System of Units [SI] units)
13. Poorly controlled hypertension defined as:
    - Systolic blood pressure (BP) > 180 mm Hg
    - Diastolic BP > 105 mm Hg
14. After 10 minutes of supine rest and confirmed by repeated measurement at screening. Subjects who fail BP screening criteria may be considered for 24-hour ambulatory blood pressure monitoring at the discretion of the investigator. Subjects who maintain a mean 24-hour BP ≤ 180/105 mm Hg with a preserved nocturnal dip of > 15% will be considered eligible.

15 Unstable angina pectoris, myocardial infarction, transient ischemic attack or stroke within 3 months prior to screening, or subjects who have undergone percutaneous coronary intervention or a coronary artery bypass graft within the past 6 months or who are due to undergo these procedures at the time of screening

16 Severe congestive heart failure (New York Heart Association Class III or IV)

17 Basal calcitonin level > 50 ng/L at screening or history/family history of medullary thyroid carcinoma or multiple endocrine neoplasia

18 History of neoplastic disease within 5 years prior to screening, except for adequately treated basal cell, squamous cell skin cancer, or *in situ* cervical cancer

19 Any positive results for serum hepatitis B surface antigen (HBsAg), hepatitis C antibody and human immunodeficiency virus (HIV) antibody

20 Substance dependence or history of alcohol abuse and/or excess alcohol intake (defined as > 21 units per week for a male subject, and >14 units per week for a female subject). Subjects must have a negative alcohol test result at screening and prior to randomisation.

21 Involvement of any AstraZeneca, MedImmune, contract research organisation, or study site employee or their close relatives

4.1.4 Subject Enrolment and Randomisation

Study participation begins (ie, a subject is "enrolled") once written informed consent is obtained. Thereafter, a subject identification (SID) number will be assigned by a central system (eg, an interactive voice/web response system [IXRS]), and the screening evaluations may begin to assess study eligibility (inclusion/exclusion) criteria. The SID number will be used to identify the subject during the screening process and throughout study participation, if applicable.

A master log of all consented subjects will be maintained at the site and will document all screening failures (ie, subjects who are consented but do not meet study eligibility criteria and/or are not randomised), including the reason(s) for screening failure.

Subjects who fail to meet the eligibility criteria (ie, screening failures) should not be randomised or receive investigational product. Subjects who are enrolled, but subsequently found not to meet all the eligibility criteria must not be randomised or initiated on treatment, and must be withdrawn from the study.

Subjects may be rescreened for Part B if they were found to be eligible for Part A but were unable to participate in the study and were not randomised. Otherwise, subjects may be rescreened once if, in the opinion of the investigator, there is a reason to believe they may be eligible.

4.1.5 Withdrawal from the Study

Subjects are at any time free to withdraw from the study (investigational product and assessments) without prejudice to future treatment (withdrawal of consent). At the time of withdrawing consent, subjects will be asked about the reason(s) for withdrawing and the presence of any AEs. After consent is withdrawn, no further study visits or data collection should take place.

4.1.6 Discontinuation of Investigational Product

An individual subject will not receive any further investigational product if any of the following occur in the subject in question (applies for both Part A and Part B of the study):

- Withdrawal of consent from further treatment with investigational product or lost to follow-up
- An AE that, in the opinion of the investigator or the sponsor, contraindicates further dosing
- Subject is determined to have met one or more of the exclusion criteria or failed to meet all of the inclusion criteria for study participation at study entry and continuing investigational product, in the decision of the investigator, might constitute a safety risk
- Subject noncompliance that, in the opinion of the investigator or sponsor, warrants withdrawal (eg, refusal to adhere to scheduled visits).
- Pregnancy in a female subject

Subjects who are permanently discontinued from receiving investigational product will be followed for protocol-specified assessments including follow-up of any AEs unless consent is withdrawn from further study participation (Section 4.1.5), the subject is lost to follow-up, the subject starts alternative treatment, or the subject is enrolled in another clinical study.

4.1.7 Replacement of Subjects

Subjects who withdraw from the study will be replaced where possible. Up to a maximum of 3 subjects will be replaced in Part A and 5 subjects in Part B.

4.1.8 Withdrawal of Informed Consent for Data and Biological Samples

Biological Samples Obtained for the Main Study

Study data are protected by the use of a SID number, which is a number specific to the subject. The investigator is in control of the information that is needed to connect a study sample to a subject. A subject's consent to the use of data does not have a specific expiration date, but the subject may withdraw consent at any time by notifying the investigator. If consent is withdrawn, any samples collected prior to that time may still be given to and used by the sponsor but no new data or samples will be collected unless specifically required to monitor safety of the subject.

Plasma and Serum Samples Obtained for Future Non-genetic Research

Plasma and serum samples obtained for future non-genetic research will be used for hypothesis-driven metabolomic studies and may also be analysed for exploratory biomarkers to assess correlations with disease activity, effects of MEDI0382, clinical outcomes, and toxicity according to the results obtained for the main study. Study data are protected by the use of a SID number, which is a number specific to the subject. The investigator is in control of the information that is needed to connect a study sample to a subject. A subject's consent to the use of data does not have a specific expiration date, but the subject may withdraw consent at any time by notifying the investigator. If consent is withdrawn, any samples collected prior to that time may still be given to and used by the sponsor but no new data or samples will be collected unless specifically required to monitor safety of the subject.

Blood Samples Obtained for Future Genetic Research [redacted]

Plasma and serum samples obtained for future genetic research or [redacted] earch will be labelled with a sample identification number linked to the SID number but will not be labelled with personal identifiers such as the subject's name. If the subject withdraws consent for participating in the genetic research [redacted], the sponsor will locate the subject's sample and destroy it. The coding of samples and results is to ensure that these research results are kept confidential by keeping the subject's identity and these results separate.

If the subject consents to have his/her samples used for genetic research or future microbiome research, this additional research may not start immediately and may start at any time during the storage period. The subject's sample(s) including any specimens of extracted deoxyribonucleic acid will be stored by the sponsor with similar samples from other subjects at a secure central laboratory. The subject's samples will not be kept for more than 15 years after the end of the study in which they were collected. If the subject chooses not to allow his/her study samples to be used for genetic research or [redacted], the samples will be destroyed by the sponsor once they are no longer required for the main study.

If consent is withdrawn after a sample has been taken but before the subject's sample is sent to the sponsor for genetic research or [redacted] the investigator will arrange to have it destroyed. If consent is withdrawn after the subject's sample(s) have been sent to the sponsor for genetic research or [redacted], the sponsor and the investigator will ensure that these sample(s) are destroyed unless the sample identification number has been removed and the subject can no longer be linked to any sample(s). However, if the subject's samples have already been used for research, the sponsor is not required to destroy results of this research. In this case only the remaining sample(s) will be destroyed.

4.2 Schedule of Study Procedures

The coronavirus disease (COVID-19) pandemic and associated guidelines, recommendations, national laws, and local restrictions are constantly evolving. Thus, where possible, other measures for carrying out protocol-related activities, such as but not limited to home nursing, may be required to ensure the safety of subjects.

4.2.1 Enrolment/Screening Period

Table 6 shows all study procedures to be conducted at the screening visit.

Whenever vital signs, 12-lead ECGs, and blood draws are scheduled for the same nominal time, the blood draws should occur last. The timing of the first 2 assessments should be such that it allows the blood draw (eg, pharmacokinetics [PK] blood sample) to occur at the proper nominal time.

Table 6 Schedule of Screening Procedures

**Study Period**	**Screening**
**Visit Number**	**V1**
**Procedure / Study Day**	**Day -65 to Day -5**
Written informed consent/assignment of SID number	X
Optional informed consent for sample for future genetic research	X
Optional informed consent for stool samples for future microbiome research	X
Medical history, including smoking and alcohol history	X
Physical examination (full) including structured neurological examination [a]	X
Verify eligibility criteria	X
Weight, height and BMI calculation	X
Demographics	X
12-lead ECG [b]	X
Vital signs (BP[c], pulse, body temperature, respiratory rate)	X
**Collect blood samples for:**	
LFTs, Cr and eGFR calculation only	X
HbA1c	X
Calcitonin	X

Table 6 Schedule of Screening Procedures

**Study Period**	**Screening**
**Visit Number**	**V1**
**Procedure / Study Day**	**Day -65 to Day -5**
HIV-1 and -2 antibodies; hepatitis B and C serology	X
**Collect urine samples for:**	
Urinalysis	X
Urine drug screen and alcohol breath screen	X
Urine or serum pregnancy test (females of childbearing potential only)	X
SC self-injection training (including priming and dosing instruction for the pen in Part B) [d]	X
Concomitant medications	X

BMI = body mass index; BP = blood pressure; Cr = creatinine; ECG = electrocardiogram; eGFR = estimated glomerular filtration rate; FBC = full blood count; HbA1c = glycated haemoglobin; HIV = human immunodeficiency virus; LFTs = liver function tests (albumin, alanine transaminase, aspartate transaminase; alkaline phosphatase and total bilirubin); SID = subject identification; SC = subcutaneous.

[a] Only the screening physical examination will be a full examination including a structured neurological examination. For all time points thereafter, only an abbreviated physical examination is required.

[b] A single digital ECG recording should be performed after the subject has rested for 10 minutes.

[c] Blood pressure should be measured once at heart level in the non-dominant arm where possible, with the subject supine and rested for 10 minutes prior to the measurement.

[d] Subject's ability to self-administer investigational product will be verified using normal saline SC injections.

4.2.2 Randomised Treatment Period (Part A and Part B)

Table 7, Table 8, Table 9, and Table 10 show all procedures to be conducted during the treatment period.

In addition, an example timetable for standardised meals, $^{2}H_2O$ administration, MMTTs, and serial MRS scans is included in Section 10.8.

Whenever vital signs, 12-lead ECGs, and blood draws are scheduled for the same nominal time, blood draws should occur last. The timing of the first 2 assessments should be such that it allows the blood draw (eg, PK blood sample) to occur at the proper nominal time.

Table 7 Schedule of Treatment Period Study Procedures (Day -4 through Day 15) (Part A)

Study Period	Outpatient	Baseline Measurements				Treatment Phase	
Visit Number		V2				V3	V4
		Inpatient				Outpatient	
Procedure / Study Day	Day -4	Day -3	Day -2	Day -1	Day 1[a]	Day 8	Day 15
Metformin washout for 5 days [b]	X	X	X	X	X		
Admission to clinical unit		X					
Discharge from clinical unit					X		
Outpatient visit to clinical unit						X	X
Fasting overnight required [c]			X	X		X (Day 7)	
Physical examination (abbreviated)		X					
Weight					X	X	X
ECG					X		
Vital signs (BP, pulse, body temperature, respiratory rate)		X			X	X	X
**Collect blood samples for: [a]**							
Serum chemistry		X			X		X
Haematology		X			X		
Serum calcitonin, amylase, and lipase					X		
Fasting amino acids, FFAs, lipids, BHB, acetoacetate, and lactate				X			
Optional blood sample for future genetic research [d]					X		

Table 7 Schedule of Treatment Period Study Procedures (Day -4 through Day 15) (Part A)

Study Period	Outpatient	Baseline Measurements				Treatment Phase	
Visit Number		V2				V3	V4
		Inpatient				Outpatient	
**Procedure / Study Day**	**Day -4**	**Day -3**	**Day -2**	**Day -1**	**Day 1[a]**	**Day 8**	**Day 15**
Blood samples for non-genetic future research [e]				X	X	X	
MEDI0382 Pharmacokinetics [f]					X		X
Immunogenicity (ADA)					X	X	
^{2}H labelled glucose samples [g]			X	X	X		
[redacted]			X			X	
Urinalysis					X		
Urine or serum pregnancy test (females of child bearing potential only)		X					X
Alcohol and illicit drug test		X					
Standardised meals [i]		X	X	X			
Deuterated water (2H2O) administration [j]			X				
MMTT and serial blood sampling for glucose levels [k]				X			
Serial MRS scans [l]				X	X		
Assessment of AEs/SAEs [m]		X	X	X	X	X	X
Concomitant medications		X	X	X	X	X	X
SC self-injection training [n]		X					
Verify eligibility criteria		X	X	X	X		

Table 7 Schedule of Treatment Period Study Procedures (Day -4 through Day 15) (Part A)

Study Period	Outpatient	Baseline Measurements				Treatment Phase	
Visit Number		V2				V3	V4
		Inpatient				Outpatient	
**Procedure / Study Day**	**Day -4**	**Day -3**	**Day -2**	**Day -1**	**Day 1[a]**	**Day 8**	**Day 15**
Randomisation [o]				X	X		
**Glucose meter and diary provision [p]**					X		
**Investigational product administration in clinic [q]**					X	X	X
**Dispense investigational product [r]**					X	X	X
**Dose up-titration**						X	X

ADA = anti-drug antibody; AE = adverse event; BHB = beta hydroxybutyrate; BP = blood pressure; C = carbon; SC = subcutaneous; ECG = electrocardiogram; FFAs = fasting free fatty acids; hr = hours; min = minutes; MMTT = mixed-meal tolerance test; MRS = magnetic resonance spectroscopy; PK = pharmacokinetic; SAE = serious adverse event; SC = subcutaneous; T = time; V = Visit.

a All blood samples (including blood samples for PK) collected on Day 1 should be collected prior to investigational product administration.

b Subjects should only commence washout of metformin if eligibility has been confirmed at Visit 1 and may resume taking metformin on Day 2. If the subject fails to meet inclusion/exclusion criteria between Day -3 and the final eligibility check (which may take place at any time from Day -3 to Day 1(predose)) the subject should re-commence the metformin with immediate effect. The site should inform the subject to ensure that they stop taking metformin from the morning of Day -4.

c For the overnight fast on Day -2, subjects should fast for 14 hr with no solids or liquids (including water); they are permitted to drinks sips of deuterated water during this period. On Day -1, subjects should again fast for 14 hr overnight but they are permitted to drink clear liquids during this time. On Day 7 prior to their outpatient visit, subjects should be asked to fast for at least 8 hr.

d Separate consents for collection of optional blood samples for genetic research and stool samples must be obtained before these samples are collected.

e Blood samples for non-genetic testing should be collected from subjects on Day -1 and Day 1 at the same time as blood samples are collected for 2H labelled glucose at T = 0, 220 min (± 5 min), 9 hr (± 30 min), 14 hr (± 30 min) and 24 hr (± 30 min) post morning standardised liquid meal administered at 0800 on Day -1. Note that the time of blood sampling may be adjusted to 1 hr before or 1 hr after provided all other related procedures are performed at the same time intervals. A single sample will be taken at Day 8 (Visit 3).

f PK blood sample is to be collected from subjects predose on Day 1 and Day 15.

g Blood samples for measurement of 2H labelled glucose should be collected from subjects just prior to deuterated water administration on Day -2 at 1800 (± 10 min). Further blood samples for measurement of 2H labelled glucose will then be collected at the same time as the blood samples for nongenetic testing (see footnote e) following the morning (0800 ± 15 min) standardised liquid meal at T = 0, 220 min (± 5 min), 9 hr (± 30 min), 14 hr (± 30 min) and 24 hr. Note that the time of blood sampling may be adjusted to 1 hr before or 1 hr after provided all other related procedures are performed at the same time intervals.

Table 7 Schedule of Treatment Period Study Procedures (Day -4 through Day 15) (Part A)

Study Period	Outpatient	Baseline Measurements				Treatment Phase	
Visit Number		V2				V3	V4
		Inpatient				Outpatient	
Procedure / Study Day	Day -4	Day -3	Day -2	Day -1	Day 1[a]	Day 8	Day 15

h [redacted] on Day -2 at 1800 (± 15 min) and 2200 (± 15 min). The subject should then avoid drinking any additional water until 0800 the next morning. Note that the start time of the deuterated water administration may be adjusted to 1hr before or 1 hr after provided all other related procedures are performed at the same time intervals.

k Following a minimum 14 hr fast, a MMTT will be performed at 0800 ± 15 min on Day -1; baseline blood samples will be collected at T = 0 (alongside samples for other blood tests and ^{2}H labelled glucose); a standardised liquid meal co-administered with C-13 labelled glucose (as required) will be given to subjects and consumed in entirety within 5 min. Further blood samples for glucose levels will be collected at T = 15, 30, 45, 60, 120, 180 and 220 min (± 5 min). Note that the start time of the MMTT may be adjusted to 1 hr before or 1 hr after provided all other related procedures are performed at the same time intervals.

l MRS scans will be performed on Day -1 at 0700, 1200, 1700 and 2200 (± 15 min) and on Day 1 at 0800 (± 15 min). Note that times may be adjusted to 1 hr before or 1 hr after provided all other related procedures are performed at the same time intervals.

m AEs will be assessed from the start of investigational product administration; SAEs will be assessed from the time of signed informed consent form.

n Subcutaneous injection training may be performed with saline prior to Day 1 and with the investigational product on Day 1; subjects should be provided with as much training as they need to be comfortable with SC self-injection between Day -3 through Day 1.

o On Day -1 or Day 1, subjects will be randomised to receive investigational product: MEDI0382 or placebo. Subjects enrolled in Part A will not be re-enrolled in Part B.

p A glucose meter, test strips, and a diary should be provided to the subject. The subject should be trained in its use and advised to test their capillary blood glucose at any time the subject feels unwell or has symptoms of hypoglycaemia (hunger, dizziness, shaking, sweating, or irritability) and they should record these symptoms and a capillary plasma glucose level in their diary only when symptomatic. Subjects should do this until their final study visit and bring the diary card with them to their next visit if they recorded symptoms or capillary plasma glucose levels while at home.

Table 7 Schedule of Treatment Period Study Procedures (Day -4 through Day 15) (Part A)

Study Period	Outpatient	Baseline Measurements				Treatment Phase	
Visit Number		V2				V3	V4
		Inpatient				Outpatient	
Procedure / Study Day	Day -4	Day -3	Day -2	Day -1	Day 1[a]	Day 8	Day 15

[q] Investigational product administration should occur in the clinic on Day 1 (Visit 2) after the final MRS scan and blood sample collection (Day 1 first dose of MEDI0382 100 µg Daily investigational product administration should then be performed by the subject at home on Day 2 through Day 7 (Day 7 is the last dose of MEDI0382 at a dosage level of 100 µg. The subject will receive the first dose of MEDI0382 200 µg in clinic on Day 8 (Visit 3) at any time in the morning during the clinic visit. The subject will then self-administer investigational product at home daily on Day 9 through Day 14 (Day 14 is the last dose of MEDI0382 at a dose level of 200 µg. The subject will receive the first dose of MEDI0382 300 µg in the clinic on Day 15 (Visit 4) at any time in the morning after blood sampling for PK. The subject will then dose at home daily on Day 16 through to Day 25. MEDI0382 300 µg will be administered in the clinic at on Days 26, 27 and 28; the final dose is on Day 28 (Visit 5).

[r] MEDI0382 or placebo will be dispensed on Days 1, 8, and 15.

Table 8 Schedule of Treatment Period Study Procedures (Day 25 through Day 29) (Part A)

Study Period	Outpatient	Treatment Phase			
Visit Number		V5			
		Inpatient			
**Procedure / Study Day**	**Day 25**	**Day 26**	**Day 27**	**Day 28 [a]**	**Day 29**
Metformin washout for 5 days [b]	X	X	X	X	X
Admission to the clinical unit		X			
Assessment of AE/SAEs [c] and review of concomitant medications		X	X	X	X
Discharge from the clinical unit					X
Fasting overnight for a minimum of 14 hours required			X	X	
Physical examination (abbreviated), ECG, and urine (or serum) pregnancy test		X			
Weight and urinalysis					X
Vital signs (BP, pulse, body temperature, respiratory rate)		X			X
**Collection of blood samples for: [a]**					
Serum chemistry, haematology, HbA1c, serum calcitonin, amylase, and lipase					X
Fasting amino acids, FFAs, lipids, BHB, acetoacetate and lactate				X	
MEDI0382 Pharmacokinetics [d]				X	
Immunogenicity (ADA) [a]				X	
Blood samples for non-genetic future research [e]				X	X
Blood samples for 2H labelled glucose [f]			X	X	X
[redacted]			X		

Table 8 Schedule of Treatment Period Study Procedures (Day 25 through Day 29) (Part A)

Study Period	Outpatient	Treatment Phase			
Visit Number		V5			
		Inpatient			
**Procedure / Study Day**	**Day 25**	**Day 26**	**Day 27**	**Day 28** [a]	**Day 29**
Standardised meals [h]		X	X	X	
Deuterated water (2H2O) administration [i]			X		
MMTT and serial blood sampling for glucose levels [j]				X	
Serial MRS scans [k]				X	X
**Investigational product administration in clinic** [l]		X	X	X	
**Dispense investigational product** [m]		X			

ADA = anti-drug antibody; AE = adverse event; BHB = beta hydroxybutyrate; BP = blood pressure; ECG = electrocardiogram; eCRF = electronic case report form; FFAs = fasting free fatty acids; HbA1c = glycated haemoglobin; hr = hours; IP = investigational product; min = minutes; MMTT = mixed-meal tolerance test; PK = pharmacokinetic; SAE = serious adverse event; V = Visit.

[a] Baseline blood samples (including blood samples for PK) collected on Day 28 should be collected prior to investigational product administration

[b] The site should inform the subject to stop taking metformin from the morning of Day 25. Subjects may resume taking metformin on Day 30.

[c] AEs will be assessed from the start of investigational product administration; SAEs will be assessed from the time of signed informed consent form.

[d] PK blood sample is to be taken predose on Day 28.

[e] Blood samples for non-genetic testing will be collected on Day 28 and Day 29 at the same time as blood samples are collected for 2H labelled glucose and at T = 0, 4 (± 30 min), 9 hr (± 30 min), 14 hr (± 30 min) and 24 hr (± 30 min) post morning standardised liquid meal administered at 0800 on Day 28. Note that the time of blood sampling may be adjusted to ± 1 hr provided all other related procedures are performed at the same time intervals.

[f] Blood samples for measurement of 2H labelled glucose should be collected just prior to deuterated water administration on Day 27 at 1800 (± 10 min). Further blood samples for measurement of 2H labelled glucose (along with blood samples for non-genetic testing, see footnote e) should be collected on Day 28 and Day 29 following the 0800 morning standardised liquid meal administered on Day 28 at T = 0, 220 min (± 5 min), 9 hr (± 30 min), 14 hr (± 30 min) and 24 hr. Note that the time of sampling may be adjusted to ± 1 hr provided all other related procedures are performed at the same time intervals.

[g] [redacted]

Table 8 Schedule of Treatment Period Study Procedures (Day 25 through Day 29) (Part A)

Study Period	Outpatient	Treatment Phase			
Visit Number		V5			
		Inpatient			
Procedure / Study Day	Day 25	Day 26	Day 27	Day 28 [a]	Day 29

h [redacted]

i [redacted] water should be administered on Day 27 at 1800 and 2200 (± 15 min). The subject should then avoid drinking any additional water until 0800 the next morning. Subjects are permitted to drink small quantities of deuterated water during the fast if thirsty. Note that the start time of the deuterated water administration may be adjusted to 1 hr before or 1 hr after provided all other related procedures are performed at the same time intervals.

j Following a minimum 14-hour fast, a MMTT will be performed at 0800 (± 15 min) on Day 28. Baseline samples will be collected at T = 0 (alongside samples for other blood tests and 2H labelled glucose); a standardised liquid meal co-administered with C-13 labelled glucose (as required) and will be given to subjects and should be consumed in entirety within 5 min. Further blood samples will be collected at T = 15, 30, 45, 60, 120, 180 and 220 min (± 5 min) for 2H labelled glucose levels. Note that the start time of the MMTT may be adjusted ± 1 hr after provided all other related procedures are performed at the same time intervals.

k MRS scans will be performed on Day 28 at 0700, 1200, 1700 and 2200 (± 15 min) and on Day 29 at 0800 (± 15 min). Note that times may be adjusted to ± 1 hr provided all other related procedures are performed at the same time intervals.

l Investigational product administration should occur at home by the subject on Day 16 through 25 and in the clinic at 0800 (± 15 min) on Day 26, Day 27, and Day 28. Note that times may be adjusted to ± 1 hr on Day 28 provided all other related procedures are performed at the same time intervals. Investigational product should be administered on Day 28 just after blood samples have been collected for PK, ADA, 2H labelled glucose, gluconeogenic substrates, and non-genetic research.

m MEDI0382 or placebo will be dispensed on Day 26

Table 9 Schedule of Treatment Period Study Procedures (Day -4 through Day 22) (Part B)

Study Period	Outpatient	Baseline Measurements				Treatment Phase		
Visit Number		V2				V3	V4	V5
		Inpatient				Outpatient		
**Procedure / Study Day**	**Day -4**	**Day -3**	**Day -2**	**Day -1**	**Day 1[a]**	**Day 8**	**Day 14/ 15**	**Day 21/22**
Metformin washout for 5 days [b]	X	X	X	X	X			
Admission to clinical unit		X						
Discharge from clinical unit					X			
Outpatient visit to clinical unit						X		
Remote contact							X	X
Fasting overnight required [c]			X	X		X (Day 7)		
Physical examination (abbreviated)		X						
Weight					X	X		
ECG					X			
Vital signs (BP, pulse, body temperature, respiratory rate)		X			X	X		
**Collect blood samples for: [a]**								
Serum chemistry		X			X	X		
Haematology		X			X			
Serum calcitonin, amylase, and lipase					X			
Fasting amino acids, FFAs, lipids, BHB, acetoacetate, and lactate				X				
Optional blood sample for future genetic research [d]					X			
Blood samples for non-genetic future research [e]				X	X	X		

Table 9 Schedule of Treatment Period Study Procedures (Day -4 through Day 22) (Part B)

**Study Period**	**Outpatient**	**Baseline Measurements**				**Treatment Phase**		
**Visit Number**		**V2**				**V3**	**V4**	**V5**
		**Inpatient**				**Outpatient**		
**Procedure / Study Day**	**Day -4**	**Day -3**	**Day -2**	**Day -1**	**Day 1[a]**	**Day 8**	**Day 14/ 15**	**Day 21/22**
MEDI0382 Pharmacokinetics [f]					X			
Immunogenicity (ADA) [f]					X			
2H labelled glucose samples [g]			X	X	X			
[redacted]			X			X		
Urinalysis					X			
Urine or serum pregnancy test (females of child bearing potential only)		X				X		
Alcohol and illicit drug test		X						
Standardised meals [i]		X	X	X				
Deuterated water (2H2O) administration [j]			X					
MMTT and serial blood sampling for glucose levels [k]				X				
Serial MRS scans [l]				X	X			
Assessment of AEs/SAEs [m]		X	X	X	X	X	X	X

Table 9 Schedule of Treatment Period Study Procedures (Day -4 through Day 22) (Part B)

Study Period	Outpatient	Baseline Measurements				Treatment Phase		
Visit Number		V2				V3	V4	V5
		Inpatient				Outpatient		
Procedure / Study Day	Day -4	Day -3	Day -2	Day -1	Day 1[a]	Day 8	Day 14/ 15	Day 21/22
Concomitant medications		X	X	X	X	X	X	X
Training in use of pen (including priming and dosing instructions for the pen) [n]		X						
Verify eligibility criteria		X	X	X	X			
Randomisation [o]				X	X			
**Glucose meter and diary provision [p]**					X			
**Investigational product administration in clinic [q]**					X	X		
**Dispense investigational product [r]**					X	X		
**Dose up-titration [s]**						X	X	X#

ADA = anti-drug antibody; AE = adverse event; BHB = beta hydroxybutyrate; BP = blood pressure; C = carbon; SC = subcutaneous; ECG = electrocardiogram; FFAs = fasting free fatty acids; hr = hours; min = minutes; MMTT = mixed-meal tolerance test; MRS = magnetic resonance spectroscopy; PK = pharmacokinetic; SAE = serious adverse event; SC = subcutaneous; T = time; V = Visit.

a All blood samples (including blood samples for PK) collected on Day 1 should be collected prior to investigational product administration.

b Subjects should only commence washout of metformin if eligibility has been confirmed at Visit 1 and may resume taking metformin on Day 2. If the subject fails to meet inclusion/exclusion criteria between Day -3 and the final eligibility check (which may take place at any time from Day -3 to Day 1(predose)) the subject should re-commence the metformin with immediate effect. The site should inform the subject to ensure that they stop taking metformin from the morning of Day -4.

Table 9 Schedule of Treatment Period Study Procedures (Day -4 through Day 22) (Part B)

Study Period	Outpatient	Baseline Measurements				Treatment Phase		
Visit Number		V2				V3	V4	V5
		Inpatient				Outpatient		
Procedure / Study Day	Day -4	Day -3	Day -2	Day -1	Day 1[a]	Day 8	Day 14/ 15	Day 21/22

c For the overnight fast on Day -2, subjects should fast for 14 hr with no solids or liquids (including water); they are permitted to drinks sips of deuterated water during this period. On Day -1, subjects should again fast for 14 hr overnight but they are permitted to drink normal water during this time. On Day 7 prior to their outpatient visit, subjects should be asked to fast for at least 8 hr.

d Separate consents for collection of optional blood samples for genetic research and stool samples must be obtained before these samples are collected.

e Blood samples for non-genetic testing should be collected from subjects on Day -1 and Day 1 at the same time as blood samples are collected for 2H labelled glucose at T = 0, 220 min (± 5 min), 9 hr (± 30 min), 14 hr (± 30 min) and 24 hr (± 30 min) post morning standardised liquid meal administered at 0800 on Day -1. Note that the time of blood sampling may be adjusted to 1 hr before or 1 hr after provided all other related procedures are performed at the same time intervals. A single sample will be taken at Day 8 (Visit 3).

f PK and ADA samples should be collected predose on Day 1 and do not need to be collected in subjects assigned to liraglutide

g Blood samples for measurement of 2H labelled glucose should be collected from subjects just prior to deuterated water administration on Day -2 at 1800 (± 10 min). Further blood samples for measurement of 2H labelled glucose will then be collected at the same time as the blood samples for nongenetic testing (see footnote e) following the morning (0800 ± 15 min) standardised liquid meal at T = 0, 220 min (± 5 min), 9 hr (± 30 min), 14 hr (± 30 min) and 24 hr. Note that the time of blood sampling may be adjusted to 1 hr before or 1 hr after provided all other related procedures are performed at the same time intervals.

h [redacted]

i Standardised solid meals are to be given to subjects 3 times daily on Day -3 and Day -2 at 0900 (± 30 min), 1300 (± 30 min), and 1700 (± 30 min); each meal is to be consumed within 15 min. On Day -1, a standardised liquid meal (as part of a MMTT) should be administered at 0800 (± 15 min). Following the MMTT, subjects will be given standardised solid meals at 1400 (± 5 min) and 1800 (± 5 min). Note that the time of standardised meals may be adjusted to 1hr before or 1 hr after provided all other related procedures are performed at the same time intervals. On Day -1 after the evening meal at 1800 (± 5 min), subjects should remain fasted overnight for 14 hours until after the T=24h MRS scan and predose laboratory assessments are performed on Day 1. Subjects are permitted to drink normal water during this period.

j Deuterated water should be administered on Day -2 at 1800 (± 15 min) and 2200 (± 15 min). The subject should then avoid drinking any additional water until 0800 the next morning. Note that the start time of the deuterated water administration may be adjusted to 1hr before or 1 hr after provided all other related procedures are performed at the same time intervals.

Table 9 Schedule of Treatment Period Study Procedures (Day -4 through Day 22) (Part B)

Study Period	Outpatient	Baseline Measurements				Treatment Phase		
Visit Number		V2				V3	V4	V5
		Inpatient				Outpatient		
Procedure / Study Day	Day -4	Day -3	Day -2	Day -1	Day 1[a]	Day 8	Day 14/ 15	Day 21/22

[k] Following a minimum 14 hr fast, a MMTT will be performed at 0800 ± 15 min on Day -1; baseline blood samples will be collected at T = 0 (alongside samples for other blood tests and 2H labelled glucose); a standardised liquid meal will be given to subjects and consumed in entirety within 5 min. Further blood samples for glucose levels will be collected at T = 15, 30, 45, 60, 120, 180 and 220 min (± 5 min). Note that the start time of the MMTT may be adjusted to 1 hr before or 1 hr after provided all other related procedures are performed at the same time intervals.

[l] MRS scans will be performed on Day -1 at 0700, 1300, 2200 (± 15 min) and on Day 1 at 0800 (24 hours post MMTT) (± 15 min). Note that times may be adjusted to 1 hr before or 1 hr after provided all other related procedures are performed at the same time intervals. The MRS scan that will be used to evaluate liver fat content should be performed whilst the subject is fasted and may occur on Day -1 or Day 1.

[m] AEs will be assessed from the start of investigational product administration; SAEs will be assessed from the time of signed informed consent form.

[n] Injection training should be performed with Placebo MEDI0382 pens prior to Day 1 and with the randomised investigational product on Day 1; subjects should be provided with as much training as they need to be comfortable with self-injection using the pen device between Day -3 through Day 1.

[o] On Day -1 or Day 1, subjects will be randomised to receive investigational product: MEDI0382 or placebo or liraglutide. Subjects enrolled in Part A will not be re-enrolled in Part B.

[p] A glucose meter, test strips, and a diary should be provided to the subject. The subject should be trained in its use and advised to test their capillary blood glucose at any time the subject feels unwell or has symptoms of hypoglycaemia (hunger, dizziness, shaking, sweating, or irritability) and they should record these symptoms and a capillary plasma glucose level in their diary only when symptomatic. Subjects should do this until their final study visit and bring the diary card with them to their next visit if they recorded symptoms or capillary plasma glucose levels while at home.

[q] Investigational product administration should occur in the clinic on Day 1 (Visit 2) after the final MRS scan and blood sample collection (Day 1 first dose of MEDI0382 50 µg or 0.6 mg liraglutide). Daily investigational product administration should then be performed by the subject at home on Day 2 through Day 7 (Day 7 is the last dose of MEDI0382 at a dosage level of 50 µg or liraglutide 0.6 mg). The subject will receive the first dose of MEDI0382 100 µg or liraglutide 1.2 mg in clinic on Day 8 (Visit 3) at any time in the morning The subject will then self-administer investigational product at home daily on Day 9 through to Day 14 (Day 14 is the last dose of MEDI0382 at a dose level of 100 µg or liraglutide 1.2 mg). On Day 14 or the morning of Day 15 prior to dosing, the site will make remote contact with the subject and remind them to make the up-titration in the MEDI0382 or placebo arms to 200 µg or liraglutide to 1.8 mg and to continue daily dosing at home. On Day 22 subjects in the MEDI0382 or placebo arms will be asked to uptitrate to 300 µg and to continue this dose until their next visit. Subjects in the liraglutide arm will be advised to continue at a dose at 1.8 mg and to continue this dose until their next visit.

[r] MEDI0382 or placebo and liraglutide will be dispensed on Days 1 and 8.

[s] #Up-titration in blinded arms only

Table 10 Schedule of Treatment Period Study Procedures (Day 32 through Day 36) (Part B)

**Study Period**	**Outpatient**	**Treatment Phase**			
**Visit Number**		**V6**			
		**Inpatient**			
**Procedure / Study Day**	**Day 32**	**Day 33**	**Day 34**	**Day 35** [a]	**Day 36**
Metformin washout for 5 days [b]	X	X	X	X	X
Admission to the clinical unit		X			
Assessment of AE/SAEs [c] and review of concomitant medications		X	X	X	X
Discharge from the clinical unit					X
Fasting overnight for a minimum of 14 hours required			X	X	
Physical examination (abbreviated), ECG, and urine (or serum) pregnancy test		X			
Weight and urinalysis					X
Vital signs (BP, pulse, body temperature, respiratory rate)		X			X
**Collection of blood samples for:** [a]					
Serum chemistry, haematology, HbA1c, serum calcitonin, amylase, and lipase					X
Fasting amino acids, FFAs, lipids, BHB, acetoacetate and lactate				X	
MEDI0382 Pharmacokinetics [d]				X	
Immunogenicity (ADA) [e]				X	
Blood samples for non-genetic future research [f]				X	X
Blood samples for ^{2}H labelled glucose [g]			X	X	X
[redacted] [h]			X		

Table 10 Schedule of Treatment Period Study Procedures (Day 32 through Day 36) (Part B)

**Study Period**	**Outpatient**	**Treatment Phase**			
**Visit Number**		**V6**			
		**Inpatient**			
**Procedure / Study Day**	**Day 32**	**Day 33**	**Day 34**	**Day 35** [a]	**Day 36**
Standardised meals [i]		X	X	X	
Deuterated water (2H2O) administration [j]			X		
MMTT and serial blood sampling for glucose levels [k]				X	
Serial MRS scans [l]				X	X
**Investigational product administration in clinic** [m]		X	X	X	
**Dispense investigational product** [n]		X			

ADA = anti-drug antibody; AE = adverse event; BHB = beta hydroxybutyrate; BP = blood pressure; ECG = electrocardiogram; eCRF = electronic case report form; FFAs = fasting free fatty acids; HbA1c = glycated haemoglobin; hr = hours; IP = investigational product; min = minutes; MMTT = mixed-meal tolerance test; MRS = magnetic resonance spectroscopy; PK = pharmacokinetic; SAE = serious adverse event; V = Visit.

[a] Baseline blood samples (including blood samples for PK) collected on Day 35 should be collected prior to investigational product administration

[b] The site should inform the subject to stop taking metformin from the morning of Day 32. Subjects may resume taking metformin on Day 36 after MRS and laboratory assessments on this day.

[c] AEs will be assessed from the start of investigational product administration; SAEs will be assessed from the time of signed informed consent form.

[d] PK blood sample is to be taken predose on Day 35.

[e] ADA samples do not need to be collected in subjects assigned to liraglutide

[f] Blood samples for non-genetic testing will be collected on Day 35 and Day 36 at the same time as blood samples are collected for 2H labelled glucose and at T = 0, 220 min (± 5 min), 9 hr (± 30 min), 14 hr (± 30 min) and 24 hr (± 30 min) post morning standardised liquid meal administered at 0800 on Day 35. Note that the time of blood sampling may be adjusted to ± 1 hr provided all other related procedures are performed at the same time intervals.

[g] Blood samples for measurement of 2H labelled glucose should be collected just prior to deuterated water administration on Day 34 at 1800 (± 10 min). Further blood samples for measurement of 2H labelled glucose (along with blood samples for non-genetic testing, see footnote e) should be collected on Day 35 and Day 36 following the 0800 morning standardised liquid meal administered on Day 35 at T = 0, 220 min (± 5 min), 9 hr (± 30 min), 14 hr (± 30 min) and 24 hr. Note that the time of sampling may be adjusted to ± 1 hr provided all other related procedures are performed at the same time intervals.

[h] [redacted]

Table 10 Schedule of Treatment Period Study Procedures (Day 32 through Day 36) (Part B)

Study Period	Outpatient	Treatment Phase			
Visit Number		V6			
		Inpatient			
Procedure / Study Day	Day 32	Day 33	Day 34	Day 35 [a]	Day 36

[i] Standardised solid meals are to be given to subjects 3 times daily on Day 33 and Day 34 at 0900 (± 30 min), 1300 (± 30 min), and 1700 (± 30 min); each meal is to be consumed within 15 minutes. On Day 35, as part of the MMTT, a standardised liquid meal is to be given to subjects in the morning at 0800 (± 15 min). Following the MMTT, subjects will be given standardised solid meals for lunch (1400 ± 5 min) and evening meal (1800 ± 5 min). Note that the time of standardised meals may be adjusted ± 1 hr provided all other related procedures are performed at the same time intervals. On Day 35 after the evening meal at 1800 (± 5 min), subjects should remain fasted overnight for 14 hours until after the T=24h MRS scan and predose laboratory assessments are performed on Day 36. Subjects are permitted to drink normal water during this period. On Day 36, after the final MRS at 0800 (± 15 min), the subject may resume their normal diet.

[j] Deuterated water should be administered on Day 34 at 1800 and 2200 (± 15 min). The subject should then avoid drinking any additional water until 0800 the next morning. Subjects are permitted to drink small quantities of deuterated water during the fast if thirsty. Note that the start time of the deuterated water administration may be adjusted to 1 hr before or 1 hr after provided all other related procedures are performed at the same time intervals.

[k] Following a minimum 14-hour fast, a MMTT will be performed at 0800 (± 15 min) on Day 35. Baseline samples will be collected at T = 0 (alongside samples for other blood tests and 2H labelled glucose); a standardised liquid meal will be given to subjects and should be consumed in entirety within 5 min. Further blood samples will be collected at T = 15, 30, 45, 60, 120, 180 and 220 min (± 5 min) for 2H labelled glucose levels. Note that the start time of the MMTT may be adjusted ± 1 hr after provided all other related procedures are performed at the same time intervals.

[l] MRS scans will be performed on Day 35 at 0700, 1300 and 2200 (± 15 min) and on Day 36 at 0800 (± 15 min). Note that times may be adjusted to ± 1 hr provided all other related procedures are performed at the same time intervals. The MRS scan that will be used to evaluate liver fat content should be performed whilst the subject is fasted and may occur on Day 35 or Day 36.

[m] Investigational product administration should occur at home by the subject on Day 9 through 32 and in the clinic at 0800 (± 60 min) on Day 33, Day 34, and Day 35. Note that times may be adjusted to ± 1 hr on Day 35 provided all other related procedures are performed at the same time intervals. Investigational product should be administered on Day 35 just after blood samples have been collected for PK, ADA, 2H labelled glucose, gluconeogenic substrates, and non-genetic research.

[n] Investigational product will be dispensed on Day 33

4.2.3 Follow-up Period

Table 11 shows all procedures to be conducted during the follow-up period.

Whenever vital signs, 12-lead ECGs, and blood draws are scheduled for the same nominal time, blood draws should occur last. The timing of the first 2 assessments should be such that it allows the blood draw (eg, PK blood sample) to occur at the proper nominal time.

Table 11 Schedule of Follow-up Procedures

Study Period	Follow-up Period
**Visit Number**	**Final (V6 (Part A) and V7 (Part B)**
**Procedure / Study Day**	**28 days post last dose (± 3 days)**
Physical examination (abbreviated)	X
Weight	X
ECG	X
Vital signs (BP, pulse, body temperature, respiratory rate) [a]	X
Serum chemistry panel	X
Calcitonin	X [c]
Haematology	X
Urinalysis	X
HbA1c	X
Urine or serum pregnancy test (females of child bearing potential only)	X
Immunogenicity (ADA) [b]	X
Assessment of AEs/SAEs	X
Concomitant medications	X

AE = adverse event; ADA = anti-drug antibody; BP = blood pressure; ECG = electrocardiogram; HbA1c = glycated haemoglobin, SAE = serious adverse event

[a] Blood pressure should be measured once at heart level in the non-dominant arm where possible, with the subject supine or seated and rested for 10 minutes prior to the measurement.

[b] Only subjects with an ADA titre ≥ 10 at the safety follow-up visit (28 days post last dose) will be asked to return to provide another ADA sample at approximately 3 months after the follow-up visit (3-month post-study sample). Subjects with a 3-month post-study ADA titre ≥ 10 will be asked to return for a sample at 3-month intervals until the titre is < 10. Subjects assigned to liraglutide do not need to have ADA samples collected (Part B only).

[c] Only to be repeated if the calcitonin level was > ULN in the sample taken at the end of dosing.

[redacted]

[redacted]

- [redacted]
- [redacted]
- [redacted]
- [redacted]
- [redacted]
- [redacted]
- [redacted]
- [redacted]
- [redacted]
- [redacted]

[redacted] [redacted]

[redacted] [redacted]

[redacted] [redacted]

[redacted]

[redacted]

[redacted]

[redacted]

[redacted]

[redacted]

[redacted]

4.3.1.2 Magnetic Resonance Spectroscopy Scans

The change in hepatic glycogen concentration will be measured using an MRS scanner with C-13 imaging capability; serial scans will be performed on Day -1, Day 1 prior to investigational product administration and on Days 28 and 29 (Part A) and Days 35 and 36 (Part B) at the time points specified in the schedules of procedures. MRS/MRI) will also be used to evaluate liver volume and hepatic fat fraction (the latter in Part B of the study only). Investigators should refer to the study manual provided for further information detailing the procedure for performing MRS and MRI scans.

4.3.2 Safety Assessments

4.3.2.1 Medical History and Physical Examination

Complete medical history will include history (including smoking and alcohol history) and current medical conditions, past or present cardiovascular disorders, respiratory, GI, renal,

hepatic, neurological, endocrine, lymphatic, haematologic, immunologic, dermatological, psychiatric, genitourinary, drug and surgical history, or any other diseases or disorders.

Physical examinations will be performed by a physician or qualified designee and will include examination of the following body systems: immunologic/allergy; head, ears, eyes, nose, and throat; respiratory; cardiovascular; GI; musculoskeletal; neurological (structured neurological examination to encompass mental status, cranial nerves, nystagmus; motor system-muscle strength, sensory system-sensation, bowel and bladder function, deep tendon reflexes, gait, station, coordination, fundoscopy, and cerebellar function); psychiatric (to the extent of determining whether or not the subject is willing and able to cooperate with the required study procedures in the investigator's judgment); dermatological; haematologic/lymphatic; and, endocrine.

Any focal deficit identified at baseline should be documented in the eCRF.

The full physical examination including a structured neurological examination is required at screening (Table 6). Abbreviated physical examinations (evaluation of selective body systems at the judgment of the physician or qualified designee based on subject presentation) are sufficient for the remaining time points (ie, randomised treatment and follow-up periods) (Table 7, Table 8, Table 9, Table 10, and Table 11).

Clinically significant abnormal findings will be recorded.

4.3.2.2 Assessment of the Injection Site

Study centre staff will check the injection site for injection site reactions during study visits as required. Injection site reactions may include (but are not limited to) local erythema, pain, tenderness, induration, swelling, pruritus, ulceration, and pigmentation.

4.3.2.3 Electrocardiograms

At the visits specified in the schedules of procedures, ECGs will be obtained after 10 minutes supine rest. Only a single digital ECG recording will be required at the specified time points.

The same recorder will be used for each subject at each time point, if possible. Date and time settings should be checked at the start of each study day and aligned with an official timekeeper for all machines used in the study.

Skin preparation should be thorough and electrode positions should be according to standard 12-lead ECG placement.

In this study lead V2 or II will be analysed and reported as primary. Lead V5 will be analysed, for all visits, as backup for the individual where analysis in lead V2 or II is not deemed possible for predose or significant parts of whole visits or whole visits.

The following variables will be reported: heart rate, RR, PR, QRS, and QT intervals from the primary lead of the digital 12-lead ECG. The investigator may add extra 12-lead ECG safety assessments if there are any abnormal findings or if the investigator considers it is required for any other safety reason.

4.3.2.4 Vital Signs

Vital sign measurements (BP, heart rate, body temperature, and respiration rate) will be obtained after the subject has rested in either a seated or supine position for at least 10 minutes at the time points specified in the schedules of procedures (for time points where ECG recording precedes vital sign measurement, the 10-minute rest in the supine position prior to the ECG suffices for the rest prior to vital sign measurement). Blood pressure should be measured once at heart level in the non-dominant arm where possible, with the subject seated or supine and rested for 10 minutes prior to the measurement. Route of body temperature measurement will be according to local protocols.

4.3.2.5 Weight, Height, and Body Mass Index Calculation

Weight (kg), height (cm), and BMI (kg/m^2) will be measured at screening. Weight will be measured at all other time points specified in the schedules of study procedures.

4.3.3 Clinical Laboratory Tests

A Laboratory Manual will be provided to the sites that specifies the procedures for collection, processing, storage, and shipment of samples, as well as laboratory contact information, specific to this clinical research study.

Clinical laboratory safety tests including serum pregnancy tests will be performed in a licensed central or licensed local clinical laboratory. Urine pregnancy tests may be performed at the site using a licensed test (dipstick). Abnormal laboratory results should be repeated as soon as possible (preferably within 24 to 48 hours).

The following clinical laboratory tests will be performed (refer to time points in the schedules of procedures):

Haematology Panel

White blood cell count with differential	Platelet count
Red blood cell count	Mean corpuscular volume
Haematocrit	Mean corpuscular haemoglobin concentration
Haemoglobin	

Serum Chemistry Panel

Calcium	ALP
Potassium	Total bilirubin

Sodium	Creatinine
Bicarbonate*	Blood urea
AST	Albumin
ALT	Magnesium

ALP = alkaline phosphatase; ALT = alanine transaminase; AST = aspartate transaminase.
Notes:
Liver function tests = AST, ALT, ALP, total bilirubin, and albumin
Tests for AST, ALT, ALP, and total bilirubin must be conducted concurrently and assessed concurrently.
* may be measured via serum chemistry sample of blood gas analyser per local procedures.

Urinalysis

Protein	Urine drug screen*
Glucose	Ketones
Blood	

Note: Urinalysis for protein, glucose, ketones, and blood may be performed at the site using a licensed test (dipstick).
*Illicit drug panel will test for the following drugs; amphetamines, barbituates, benzodiazepines, cocaine, opiates (morphine, heroin), methamphetamine, cannabinoids (marijuana), methodone, phencyclidene, methylenedioxy-methamphetamine (ecstasy), propoxyphene, tricyclic antidepressants. Subjects who use tricyclic antidepressants or benzodiazepines for an established clinical indication may be permitted to enter the study based upon the judgement of the investigator.

Other Laboratory Tests

- Calcitonin
- Alcohol screening test (breath or urine based test)
- Pancreatic amylase, lipase
- HbA1c
- Anti-drug antibodies (immunogenicity)
- Serum or urine pregnancy test (for females with childbearing potential only)
- HBsAg, hepatitis C antibody (screening only)
- HIV-1, -2 antibodies (screening only)
- Glucose metabolism panel for MMTT: Timed glucose
- Fasting lipid profile: high-density lipoprotein, low-density lipoprotein, triglycerides
- Gluconeogenic substrates: free fatty acids, beta-hydroxybutyrate, acetoacetate, amino acids, and lactate
- Glucose samples to measure ^{2}H labelled glucose

4.3.3.1 Glucose Meter Measured Capillary Plasma Glucose Readings

At the start of the study, each subject will be issued a standardised glucose meter, testing strips, and a diary. Subjects will be encouraged to perform finger-prick tests if they feel unwell and in particular if they feel the symptoms may be due to hypoglycaemia for the duration of the study, but they will not be required to test routinely. If the investigator/site staff feel that a subject could be experiencing hypo- or hyperglycaemia, capillary plasma glucose should be tested with a standardised glucose meter. Capillary plasma glucose levels of < 3 mmol/L (54 mg/dL) should be recorded as an AE regardless of whether the subject has symptoms or not.

4.3.4 Pharmacodynamic Evaluation and Measures

Deuterated water (^2H_2O) assessment

The test should commence approximately 1 hour after the start of the evening standardised meal. Subjects will be expected to fast after they have finished the meal until they receive the standardised meal the following morning. Subjects should avoid drinking water or other liquids during this period, but are permitted to drink 0.5% enriched ^2H_2O in small quantities if required for comfort. Subjects should be advised that $^2H_20$ may occasionally cause mild transient tinnitus.

A baseline blood test should be taken just prior to administration of the first dose of ^2H_2O (70%). The first dose of ^2H_2O is 3.25 g or 3.0mL per kg of body water; body water is calculated as 50% of total body weight for women and 60% of total body weight for men and should ideally be consumed within 15 minutes. Four hours later a second dose of ^2H_2O (3.25 g or 3.0 mL per kg of body water) should be administered to the subject and consumed within 15 minutes (Landau et al, 1995).

<u>Example dosing schedule:</u>

90 kg male subject:

1800 hours: First dose ^2H_2O; $3.0 \times 90 \times 0.6 = 162$ mL ^2H_2O

2200 hours: Second dose ^2H_2O; $3.0 \times 90 \times 0.6 = 162$mL ^2H_2O

Blood tests to be taken as detailed in (Table 7 and Table 8 [Part A] and Table 9 and Table 10 [Part B]) and will be used to measure the following:

- Change in estimated percentage gluconeogenesis as measured by the ratio of ^2H enrichment in C5 or C6 and C2 of glucose at T = 0, 4, 9, 14 and 24 hours from baseline (Day -1) to the end of 28 or 35 days of treatment
- Change in estimated percentage glycogenolysis as measured by 1- the ratio of ^2H enrichment in C5 or C6 and C2 of glucose at T = 0, 4, 9, 14 and 24 hours from baseline (Day -1) to the end of 28 or 35 days of treatment

Blood samples will be collected, labelled, stored, and shipped as detailed in the Laboratory Manual.

4.3.5 Pharmacokinetic Evaluation and Methods

Blood will be collected at protocol-specified time points to evaluate plasma concentrations of MEDI0382 up-titrated to 300 µg (Table 7 and Table 8). Blood sampling within the specified window around the specified time will not be considered a protocol deviation but the exact

time of blood sampling should be recorded. The PK of MEDI0382 in plasma will be measured utilising a validated liquid chromatography-tandem mass spectrometry method.

Blood samples will be collected to measure plasma C_{trough} concentrations of MEDI0382 at steady state (Part A, Days 15 and 28, Part B, Days 8 and 35).

Blood samples will be collected, labelled, stored, and shipped as detailed in the Laboratory Manual. Residual PK samples will be retained until 5 years after completion of the clinical study report.

4.3.6 Immunogenicity Evaluation and Methods

Blood samples will be collected at protocol-specified time points to evaluate serum ADA responses to MEDI0382 (Table 7, Table 8, Table 9, Table 10, and Table 11). Evaluations will be performed using a validated immunoassay. Samples will be analysed for development of ADAs and titre (if confirmed positive).

Serum samples collected for ADA should be stored for 2 years after marketing approval, and they may be utilised for further characterisation of the antibody response.

4.3.7 Biomarker Evaluation and Methods

The subject's consent for provision of [redacted] is optional. If the subject's consent is obtained, [redacted] will be collected at the beginning, middle (Day 8 to 15 [Part A] or Day 8 to 33 [Part B]), and the end of the study as detailed in the schedule of treatment period study procedures (Table 7 and Table 8 [Part A] and Table 9 and Table 10 [Part B]) and will be used to evaluate changes in the gut microbiome following treatment with MEDI0382.

In addition, blood samples will be collected for future nongenetic research at the time points as detailed in the schedule of treatment period study procedures (Table 7 and Table 8 [Part A] and Table 9 and Table 10 [Part B]). The blood samples are part of the main study and will be primarily used to perform hypothesis-driven metabolomic studies, and may also be analysed for exploratory biomarkers to assess correlations with disease activity, effects of MEDI0382, clinical outcomes, and toxicity. See Section 10.7 for further information.

4.3.8 Storage, Re-use, and Destruction of Biological Samples

Samples will be stored for a maximum of 15 years from the date of the last subject's last visit, after which they will be destroyed. The results of any investigation will be reported either in the clinical study report itself or as an addendum, or separately in a scientific report or publication.

4.3.9 Estimate of Volume of Blood to Be Collected

The estimated volume of blood to be collected from each subject over the entire course of their participation in the study is approximately 404 mL. Additional blood samples may be collected at the discretion of the investigator in the event of abnormal laboratory findings or an AE.

4.4 Study Suspension or Termination

The sponsor reserves the right to temporarily suspend or permanently terminate this study at any time during part A or B of the study. The reasons for temporarily suspending or permanently terminating the study may include but are not limited to the following:

- The incidence or severity of AEs in this or other studies indicates a potential health hazard to subjects (eg, drug-related SAEs, anaphylactic reaction, hepatic enzyme alterations meeting Hy's law, signs of renal toxicity, QT interval prolongation).
- Subject enrolment is unsatisfactory
- Noncompliance that might significantly jeopardise the validity or integrity of the study
- Sponsor decision to terminate development of the investigational product for this indication
- Sponsor decision to terminate the study

If MedImmune determines that temporary suspension or permanent termination of the study is required, MedImmune will discuss the reasons for taking such action with all participating investigators (or head of the medical institution, where applicable). When feasible, MedImmune will provide advance notice to all participating investigators (or head of the medical institution, where applicable) of the impending action.

If the study is suspended or terminated for safety reasons, MedImmune will promptly inform all investigators, heads of the medical institutions (where applicable), and/or institutions conducting the study. MedImmune will also promptly inform the relevant regulatory authorities of the suspension/termination along with the reasons for such action. Where required by applicable regulations, the investigator or head of the medical institution must inform the Independent Ethics Committee (IEC) promptly and provide the reason(s) for the suspension/termination. If the study is suspended for safety reasons and it is deemed appropriate by the sponsor to resume the study, approval from the relevant regulatory authorities (and IECs when applicable) will be obtained prior to resuming the study. In the event that the study is prematurely terminated, biological samples will be retained for up to 15 years as described previously unless consent is withdrawn as described in Section 4.1.8.

4.5 Investigational Products

4.5.1 Identity of Investigational Product(s)

MedImmune will provide the investigator(s) with the investigational products (Table 12) using designated distribution centres.

Table 12 Identification of Investigational Products

Investigational Product	Manufacturer	Concentration and Formulation as Supplied
**Part A**		
MEDI0382	MedImmune	[redacted]
[redacted]	[redacted]	[redacted]
[redacted]	[redacted]	[redacted]
Placebo	MedImmune	[redacted]
**Part B**		
MEDI0382 Pen	MedImmune	Solution for injection in multidose prefilled pen device containing 2.7 mL. Each pen can deliver doses of 50-600 µg, in increments of 50 µg.
Placebo Pen	MedImmune	Solution for injection in multidose prefilled pen device containing 2.7 mL. Each pen can deliver doses of 50-600 µL, in increments from 50 µL.
Liraglutide (Victoza)	Sourced from Novo Nordisk	Solution for injection in multidose prefilled pen containing 3 mL

[redacted]

MEDI0382 and placebo for use in the study will be supplied to the site in blinded kits each containing 8 prefilled syringes. Each kit has a unique number that is printed on all labels within the kit (ie, the outer carton label and the label of the prefilled syringe within the carton). When supplying the investigational product for at-home dosing, each subject will receive sufficient quantity of investigational product to last until the next visit.

[redacted]

[redacted]

Liraglutide (6.0 mg/mL, open label) as active drug will be administered by SC injection once daily for 35 days in Part B of the study using a multidose pen injector containing 3 mL of solution. Liraglutide will be repacked/relabelled by MedImmune as applicable according to regulatory requirements.

4.5.1.1 Investigational Product Handling

In-clinic Investigational Product Handling

For Part A the investigational product manager or study personnel who receives, inspects, and stores the investigational product will be unblinded. For Part B the investigational product manager is fully blinded, and therefore can remain blinded.

For Part A, the investigational product kits must be stored at 2°C to 8°C (36°F to 46°F) in their original container. For Part B, the multidose pen injector must be stored at 2°C to 8°C (36°F to 46°F) prior to use. After first use, the individual pen being used for dosing may either be stored at 2°C to 8°C (36°F to 46°F), or at room temperature (below 30°C), for up to 9 days. After 9 days, the pen should be discarded and a new pen used.

For additional information regarding investigational product, study staff at the clinic and subjects should refer to the Instructions for Use (IFU) for MEDI0382 and placebo and to the Patient Information Leaflet (PIL) for liraglutide.

At-home Investigational Product Handling

Subjects will be given training in self-administration and disposal of investigational product.

Investigational product handling steps for at-home administration will be provided within the relevant IFU booklet. IFU booklets for MEDI0382 and placebo and a PIL for liraglutide must be provided to each subject on each occasion investigational product is dispensed for at-home use. Subjects will also be asked to return used investigational product kit boxes along with sharps bins and unused prefilled syringes/pen injectors to the investigational site.

The entire kit of investigational product (MEDI0382 or placebo or liraglutide) should be stored in the refrigerator. Subjects should be asked to ensure they have a normal domestic refrigerator at home, which should be between 2°C and 8°C. Investigational product should be protected from heat and light. Subjects should be instructed to refer to the IFU of investigational product. The subject is to avoid the risk of freezing the investigational product by carefully placing the investigational product within their refrigerator, and they should not use investigational product if it has been frozen.

In Part A of the study, the subject is to remove from the refrigerator only the prefilled syringe of MEDI0382 or placebo required for their daily dose. All other syringes are to be kept in the refrigerator until required. The prefilled syringe should always be safely discarded after use. The subject will return sharps bins with used syringes and any unused prefilled syringes to the site for accountability at each outpatient/inpatient visit.

In Part B, subjects randomised to receive MEDI0382 or placebo will remove the multidose pen injector required for their daily dose from the refrigerator. Subjects randomised to receive open-label liraglutide will remove from the refrigerator the multidose pen injector required for their daily dose. The pen should always be safely discarded when empty. The subject will return sharps bins with used multidose pen injectors and any unused multidose pen injectors to the site for accountability at each outpatient/inpatient visit.

4.5.1.2 Investigational Product Inspection

For Part A: MEDI0382 is supplied as a sterile liquid solution in a prefilled syringe for single use. Each syringe selected for in-clinic treatment administration should be inspected by the unblinded investigational product manager or unblinded study personnel prior to injection. MEDI0382 is supplied at a concentration of 0.5 mg/mL. The solution should not be cloudy, discoloured, or contain any visible particles.

For Part B: MEDI0382 or placebo is supplied as a sterile liquid solution in a multi-dose pen. Each pen selected for administration should be inspected prior to injection. The solution in the cartridge should not be cloudy, discoloured, or contain any visible particles.

Liraglutide is supplied as a 6 mg/mL clear, colourless solution in a prefilled, multidose pen injector that delivers doses of 0.6 mg, 1.2 mg, or 1.8 mg. The solution in the cartridge of the pen should not be cloudy, discoloured, or contain any visible particles (Victoza package insert, 2017).

Subjects should be instructed to refer to the IFU for MEDI0382 and placebo and to the PIL for liraglutide.

If there are any defects noted with the investigational product, the investigator and site monitor should be notified immediately. Refer to the Reporting Product Complaints (Section 4.5.1.5) for further instructions.

4.5.1.3 Treatment Administration

In-clinic Treatment Administration

MEDI0382, placebo, or liraglutide should be stored at 2°C to 8°C in the original container.

The dose to be administered should be allowed to warm up at room temperature for about 30 minutes before injection. The first day of dosing with MEDI0382 or placebo (or liraglutide Part B only) is considered Day 1. On the day of each dose, investigational product will be administered according to the schedule of procedures. Investigational product (MEDI0382 or placebo) will be administered by SC injection in the lower abdomen.

Doses of liraglutide are to be administered by SC injection in the abdomen, thigh, or upper arm. The staff at the clinic should refer to the PIL for liraglutide for additional product information.

At-home Treatment Administration

Investigational product (MEDI0382 or placebo) should be removed from the refrigerator for about 30 minutes for temperature equilibration prior to dosing.

[redacted]

[redacted]

[redacted]

[redacted]

Subjects should always refer to the IFU document for at-home administration details.

For at-home self-administration of liraglutide, subjects should refer to the label on the carton containing the prefilled liraglutide multidose pen injector and to the PIL for liraglutide.

If a dose of MEDI0382, placebo, or liraglutide is missed, subjects should take the dose as soon as it is remembered unless it is almost time for the next dose, in which case subjects should skip the missed dose and take the investigational product at the next regularly scheduled time.

4.5.1.4 Monitoring of Dose Administration

As with any exogenous peptide delivered subcutaneously, allergic reactions to dose administration are possible.

In prior experience with MEDI0382 there has been one injection site reaction and no anaphylactoid reactions. During visits to the clinical unit, the site of administration will be checked for signs of injection site reaction as required (Section 4.3.2.2).

4.5.1.5 Reporting Product Complaints

Any defects with the investigational product must be reported *immediately* (ie, at the first opportunity) to the MedImmune Product Complaint Department by the site with further notification to the site monitor. All defects will be communicated to MedImmune and investigated further with the Product Complaint Department. During the investigation of the product complaint, all investigational product must be stored at labelled conditions unless otherwise instructed.

[redacted]

[redacted]

[redacted]

[redacted]

4.5.2 Additional Study Medications

Subjects entering the study must have been treated with daily stable doses of oral, blood glucose-lowering therapy with metformin for at least 3 months prior to screening. Metformin is not provided by the sponsor.

After screening, eligible subjects on metformin monotherapy will complete two 5-day metformin washout periods (at the beginning of the study starting from Day -4 with metformin dosing resuming on Day 2 and at the end of the study starting from Day 25 with metformin dosing resuming on Day 30).

4.5.3 Labelling

Labels for the investigational product will be prepared in accordance with Good Manufacturing Practice (GMP) and local regulatory guidelines. Label text will be translated into local languages, as required.

4.5.4 Storage

All investigational product should be kept in a secure place under appropriate storage conditions. The label on the investigational product kit specifies the appropriate storage.

4.5.5 Treatment Compliance

In-clinic administration

At the clinic, investigational product is administered by study site personnel, who will monitor compliance.

At-home administration

Compliance during the at-home dosing period to be monitored via returned prefilled syringes (Part A), and returned multidose pen injectors (Part B). The distribution of investigational product for self-administration should be recorded in the appropriate sections of the eCRF.

4.5.6 Accountability

The investigator's or site's designated investigational product manager is required to maintain accurate investigational product accountability records and will account for all investigational product dispensed to and returned by the subject. Study-site staff, if applicable, or the site monitor delegated to the investigational product management will account for all investigational product received at the site, unused investigational product, and for appropriate disposition of investigational product in accordance to local procedures. Upon completion of the study, copies of investigational product accountability records will be returned to MedImmune. All unused investigational product will be returned to a MedImmune-authorised depot or disposed of upon authorisation by MedImmune.

In the case of a malfunctioning prefilled syringe or a liraglutide prefilled multidose pen, the designated investigational product manager (pharmacist/study nurse) should contact the site monitor delegated to investigational product management to initiate a product complaint process according to Section 4.5.1.5.

4.6 Treatment Assignment and Blinding

4.6.1 Methods for Assigning Treatment Groups

An IXRS will be used for randomisation to a treatment group and assignment of investigational product kit numbers. A subject is considered randomised into the study when the investigator notifies the IXRS that the subject meets eligibility criteria, and the IXRS provides the assignment of investigational product kit numbers to the subject.

Eligible subjects will be randomised at a 1:1 ratio following screening to receive either MEDI0382 SC or placebo SC (Part A) or randomised at a 1:1:1 ratio following screening to receive either MEDI0382 SC, placebo SC, or open-label liraglutide (Part B). The IXRS will assign a unique randomisation code and treatment group to the subject at the time of randomisation. Subjects who withdraw from the study may be replaced, if deemed necessary by the medical monitor, to ensure that safety data are collected on a sufficient number of subjects (Section 4.1.7).

4.6.2 Methods to Ensure Blinding

Part A: This is a double-blind study in which MEDI0382 and placebo are not identical in the prefilled syringe titration volumes. Neither the subject nor any of the investigator or sponsor staff who are involved in the treatment or clinical evaluation of the subjects will be aware of the treatment received (ICH E9).

The different fill volumes of investigational product (MEDI0382 and placebo) and the relative position of the plunger rods will be visually distinct during administration. To maintain the blind in Part A, investigational product (MEDI0382 and placebo) prefilled syringes will be handled by an unblinded investigational product manager or unblinded study personnel who will not be involved in the treatment or clinical evaluation of subjects. An unblinded site monitor will perform investigational product accountability, and this will be a different person to the blinded site monitor who will oversee other aspects of the study at the clinical site.

In Part B, the MEDI0382 and placebo multidose pens are indistinguishable. Each subject will know the volume of investigational product they have been instructed to select and administer, the subject and site staff are blinded with respect to whether they are receiving MEDI0382 or placebo. Liraglutide used in Part B will be open-label.

The site will maintain a written plan detailing which staff members are blinded/unblinded and the process of investigational product administration used to maintain the blind.

4.6.3 Methods for Unblinding

4.6.3.1 Unblinding in the Event of a Medical Emergency

In the event of a medical emergency, the investigator may unblind an individual subject's investigational product allocation. Instructions for unblinding an individual subject's investigational product allocation are contained in the IXRS manual. In general, unblinding should only occur if management of the medical emergency would be different based on the subject having received investigational product. In the majority of cases, the management of a medical emergency would be the same whether or not investigational product was received by the subject. If this was the case, the investigational product allocation should not be unblinded.

MedImmune retains the right to unblind the treatment allocation for SAEs that are unexpected and are suspected to be causally related to an investigational product and that potentially require expedited reporting to regulatory authorities.

If a subject's investigational product allocation is unblinded, the subject should be discontinued from investigational product.

4.7 Restrictions During the Study and Concomitant Treatment(s)

The investigator must be informed as soon as possible about any medication taken from the time of screening until the end of the study (final study visit). Any concomitant medication(s), including herbal preparations, taken during the study will be recorded in the eCRF.

4.7.1 Permitted Concomitant Medications

Investigators may prescribe concomitant medications or treatments deemed necessary to provide adequate supportive care except for those medications identified as "excluded" as listed in Section 4.1.3. Specifically, subjects should receive full supportive care during the study, including transfusions of blood and blood products, and treatment with antibiotics, antiemetics, anti-diarrhoeals, and analgesics, and other care as deemed appropriate, and in accordance with their institutional guidelines.

4.7.2 Prohibited Concomitant Medications

Other than the medications described above, use of concomitant medications including over-the-counter medications, herbal supplements, vitamins, etc., at the times specified in the list below is not permitted. Subjects must be instructed not to take any medications, including over-the-counter products, without first consulting with the investigator.

Subjects should not be initiated on any new medications to control blood glucose levels during the screening, treatment, and follow-up period unless it is deemed necessary by the investigator for safety and following discussion with the medical monitor. Refer to Section 3.1.4.3 for further details.

Use of the following concomitant medications is not permitted:

- Concurrent or previous use of a GLP-1 analogue containing preparation within the last 30 days or 5 half-lives of the drug, whichever is longest, prior to the start of screening (Visit 1)
- Concurrent use of any herbal preparations or medicinal products licensed for control of body weight or appetite and within 1 week prior to the start of the study (Visit 2)
- Concurrent or previous use of drugs approved for weight loss (eg, orlistat, bupropion naltrexone, phentermine-topiramate, phentermine, lorcaserin) and within the last 30 days or 5 half-lives of the drug, whichever is longest, prior to the start of the study (Visit 2)
- Concurrent use of opiates, domperidone, metoclopramide, or other drugs known to alter gastric emptying and within 2 weeks prior to the start of the study (Visit 2)
- Concurrent use of glucagon or glucagon analogues within 4 weeks prior to the start of the study (Visit 2)
- Concurrent use of warfarin within 4 weeks prior to the start of the study (Visit 2)

4.8 Statistical Evaluation

4.8.1 General Considerations

Tabular summaries will be presented by treatment group. Categorical data will be summarised by the number and percentage of subjects in each category. Continuous variables will be summarised by descriptive statistics. The study objectives will be evaluated on Part A and Part B separately and also on the combined cohorts as appropriate. Additional details of statistical analyses will be described in the statistical analysis plan.

4.8.1.1 Analysis Populations

Efficacy Analysis Population

The efficacy analysis will be based on the intent-to-treat (ITT) population, which is defined as all subjects that are randomised and received at least one dose of investigational product, and subjects will be analysed according to randomised treatment assignments. All efficacy analyses will be performed on the ITT population unless otherwise specified.

Safety Analysis Population

The safety analysis will be based on the As-treated population, which includes all subjects who receive at least one dose of any investigational product and subjects will be analysed according to the treatment they actually received.

Pharmacokinetic Analysis Population

The PK population includes all subjects who received at least one dose of investigational product and had at least one PK blood sample taken that is above the lower limit of quantitation.

4.8.2 Sample Size

In Part A, the sample size of 8 completers in each of the MEDI0382 and placebo groups provides ~80% power to detect a difference of 19% reduction in the baseline glycogen concentration adjusted for liver volume (as measured by MRS at T = 4 hours post standardised morning meal) after 28 days of treatment in the MEDI0382 versus placebo arms. This is under the assumption of baseline glycogen content of 283 µmol/mL and standard deviation of 41 µmol/mL for both groups and a two-sided significance level at 0.1 (Krssak et al, 2004; Rothman et al, 1991).

In Part B, the sample size of 10 completers in each MEDI0382 and placebo groups provides > 80% power to detect a 24.2% reduction in fasting glycogen concentration adjusted for liver volume (as measured by MRS at T = 24 hours post standardised liquid morning meal) after 35 days of treatment in the MEDI0382 versus placebo arms. This is under the assumption of a common standard deviation of 17.0 % for both groups, a two-sided significance level at 0.05 and is based on the results of Part A.

4.8.3 Efficacy

4.8.3.1 Primary Efficacy Analysis

The primary endpoint, change in glycogen concentration (adjusted for liver volume) as measured by MRS at T = 4 hours post standardised morning meal from baseline (Day -1) to the end of 28 days of treatment, will be compared between the MEDI0382 and placebo groups using an analysis of covariance (ANCOVA) model adjusting for baseline value and treatment group (Part A only).

The primary endpoint, percentage change in fasting glycogen concentration (adjusted for liver volume) as measured by MRS at T = 24 hours post standardised morning meal from baseline (Day -1) to the end of 35 days of treatment, will be compared between the MEDI0382 and placebo groups using an analysis of covariance (ANCOVA) model adjusting for baseline value and treatment group.

4.8.3.2 Secondary Efficacy Analyses

The secondary endpoints, percentage change in glycogen concentration (adjusted for liver volume) as measured by MRS at T = 24 hours post standardised morning meal from baseline (Day 1) to the end of 35 days treatment, will be compared between MEDI0382 and liraglutide groups using the ANCOVA model adjusting for baseline value and treatment group in Part B of the study only. A similar analysis will be applied to measure change in hepatic fat fraction

from baseline (Day -1 or Day 1) to the end of 35 days of treatment (Day 35 or 36) versus placebo.

4.8.3.3 Exploratory Analyses

The exploratory endpoints will be summarised by treatment arms and formal statistical model analysis may be performed if deemed necessary.

4.8.4 Safety

4.8.4.1 Analysis of Adverse Events

Treatment-emergent AEs and SAEs will be summarized by type, incidence, severity and relationship to investigational product by system organ class (SOC) and preferred term (PT). Adverse events will be coded using the Medical Dictionary for Regulatory Activities (MedDRA). Specific AEs will be counted once for each subject for calculating rates, but will be presented in total in subject listings. In addition, if the same AE occurs multiple times within a particular subject, the highest severity and level of causality will be reported. If any associations of interest between AEs and baseline characteristics are observed, additional stratified results may be presented. All treatment-emergent AEs will be summarized overall and by MedDRA SOC and PT, by severity and relationship to investigational product. In addition, summaries of deaths, SAEs, and treatment discontinuations due to AEs will be provided.

4.8.4.2 Analysis of Clinical Laboratory Parameters

Other safety data, such as vital signs and clinical laboratory data, will be descriptively summarised at each time point. Change from baseline to each post baseline time point in these data will also be summarised, where appropriate. ECG parameters will also be assessed and summarised descriptively.

4.8.5 Analysis of Pharmacokinetics

Plasma C_{trough} concentrations of MEDI0382 will be summarised by dose level and by day in both Part A and Part B.

4.8.6 Analysis of Immunogenicity

ADA incidence rate and titre will be tabulated for each treatment to monitor immunogenicity. Tiered analyses will be performed to include screening, confirmatory, and titre assay components; samples confirmed positive for ADA will be tested and analysed for antibody titre and reported and may be utilised for further characterisation of the ADA response.

4.8.7 Interim Analysis

An interim analysis after the last subject completes their last visit in Part A of the study is planned.

5 ASSESSMENT OF SAFETY

5.1 Definition of Adverse Events

The ICH Guideline for Good Clinical Practice E6(R1) defines an AE as:

> Any untoward medical occurrence in a patient or clinical investigation subject administered a pharmaceutical product and which does not necessarily have a causal relationship with this treatment. An AE can therefore be any unfavorable and unintended sign (including an abnormal laboratory finding), symptom, or disease temporally associated with the use of a medicinal product, whether or not considered related to the medicinal product.

An AE includes but is not limited to any clinically significant worsening of a subject's pre-existing condition. An abnormal laboratory finding (including ECG finding) that requires medical intervention by the investigator, or a finding judged by the investigator as medically significant should be reported as an AE. If clinical sequelae are associated with a laboratory abnormality, the diagnosis or medical condition should be reported (eg, renal failure, hematuria) not the laboratory abnormality (eg, elevated creatinine, urine red blood cell increased). Abnormal laboratory values that are not, in the investigator's opinion, medically significant and do not require intervention should not be reported as AEs.

AEs may be treatment emergent (ie, occurring after initial receipt of investigational product) or nontreatment emergent. A nontreatment-emergent AE is any new sign or symptom, disease, or other untoward medical event that begins after written informed consent has been obtained but before the subject has received investigational product.

Elective treatment or surgery or preplanned treatment or surgery (that was scheduled prior to the subject being enrolled into the study) for a documented pre-existing condition that did not worsen from baseline is not considered an AE (serious or nonserious). An untoward medical event occurring during the prescheduled elective procedure or routinely scheduled treatment should be recorded as an AE or SAE.

5.2 Definition of Serious Adverse Events

An SAE is any AE that:

- Results in death
- Is immediately life-threatening
- Requires inpatient hospitalisation or prolongation of existing hospitalisation
- Results in persistent or significant disability/incapacity
- Is a congenital anomaly/birth defect in offspring of the subject

- Is an important medical event that may jeopardise the subject or may require medical intervention to prevent one of the outcomes listed above

Medical or scientific judgment should be exercised in deciding whether expedited reporting is appropriate in this situation. Examples of medically important events are intensive treatment in an emergency room or at home for allergic bronchospasm, blood dyscrasias, or convulsions that do not result in hospitalisations; or development of drug dependency or drug abuse.

5.2.1 Hepatic Function Abnormalities

Refer to Section 5.5.2 and Section 10.5 for the definition and follow-up for liver abnormalities.

5.3 Recording of Adverse Events

Adverse events will be recorded on the eCRF using a recognised medical term or diagnosis that accurately reflects the event. Adverse events will be assessed by the investigator for severity, relationship to the investigational product, possible etiologies, and whether the event meets criteria of an SAE and therefore requires immediate notification to the sponsor (Section 5.4). See Section 5.4 for the definition of SAEs and Section 10.3 for guidelines for assessment of severity and relationship. If an AE evolves into a condition that meets the regulatory definition of "serious," it will be reported in the eCRF.

5.3.1 Time Period for Collection of Adverse Events

AEs will be collected from the time of start of investigational product throughout the study including the follow-up period (28 days after the last dose of investigational product; Visit 6 [Part A] and Visit 7 [Part B]). Additionally, all SAEs will be collected from time of signature of informed consent throughout the study including the follow-up period as described above.

5.3.2 Follow-up of Unresolved Adverse Events

Any AEs that are unresolved at the subject's last visit in the study are followed up by the investigator for as long as medically indicated, but without further recording in the eCRF. MedImmune retains the right to request additional information for any subject with ongoing AE(s)/SAE(s) at the end of the study, if judged necessary. Updates regarding SAEs that were ongoing at the time of the subject's completion of study participation should be submitted to the study representative using a paper SAE follow-up form.

5.4 Reporting of Serious Adverse Events

All SAEs have to be reported, whether or not considered causally related to the investigational product, or to the study procedure(s). All SAEs will be recorded in the eCRF.

If any SAE occurs in the course of the study, then investigators or other site personnel must inform the appropriate sponsor representative(s) within 1 day, ie, immediately but no later than 24 hours after becoming aware of the event.

The designated sponsor representative works with the investigator to ensure that all the necessary information is provided to the sponsor's patient safety data entry site within 1 calendar day of initial receipt for fatal and life-threatening events and within 5 calendar days of initial receipt for all other SAEs.

For fatal or life-threatening AEs where important or relevant information is missing, active follow-up is undertaken immediately. Investigators or other site personnel inform sponsor representatives of any follow-up information on a previously reported SAE within 1 calendar day, ie, immediately but no later than 24 hours after becoming aware of the event.

Once the investigators or other site personnel indicate an AE is serious in the electronic data capture (EDC) system, an automated email alert is sent to inform the designated sponsor representative(s).

If the EDC system is not available, then the investigator or other study site personnel reports an SAE to the appropriate sponsor representative by telephone. The sponsor representative will advise the investigator/study site personnel how to proceed.

5.5 Other Events Requiring Immediate Reporting

5.5.1 Overdose

An overdose is defined as a subject receiving a dose of investigational product in excess of that specified in this protocol.

- An overdose with associated AEs is recorded as the AE diagnosis/symptoms on the relevant AE modules in the eCRF and on the Overdose eCRF module.
- An overdose without associated symptoms is only reported on the Overdose eCRF module.

If an overdose on a MedImmune investigational product occurs during the course of the study, then the investigator or other site personnel inform appropriate sponsor representatives immediately, or no later than 24 hours after becoming aware of the event.

The designated sponsor representative works with the investigator to ensure that all relevant information is provided to the sponsor's Patient Safety data entry site.

For overdoses associated with an SAE, the standard reporting timelines apply; see Section 6.5. For other overdoses, reporting must occur within 30 days.

5.5.2 Hepatic Function Abnormality

Cases where a subject shows elevation in liver biochemistry may require further evaluation and occurrences of AST or ALT ≥ 3 × ULN together with total bilirubin ≥ 2 × ULN may need to be reported as SAEs. Please refer to Section 10.5 (Appendix 5) for further instruction on cases of increases in liver biochemistry and evaluation of Hy's Law.

5.5.3 Pregnancy

All pregnancies and outcomes of pregnancy should be reported to the sponsor.

5.5.3.1 Maternal Exposure

Women of childbearing potential who are sexually active with a nonsterilised male partner are required to use one form of contraception as described in the inclusion criteria. Pregnancy should be avoided for at least 28 days after receiving investigational product or until the subject completes participation in the study, whichever is longer. If a subject becomes pregnant during the course of the study, investigational product should be discontinued immediately.

Pregnancy itself is not regarded as an AE unless there is a suspicion that the investigational product under study may have interfered with the effectiveness of a contraceptive medication. Congenital abnormalities/birth defects and spontaneous miscarriages should be reported and handled as SAEs. Elective abortions without complications should not be handled as AEs. The outcome of all pregnancies (spontaneous miscarriage, elective termination, ectopic pregnancy, normal birth or congenital abnormality) should be followed up and documented even if the subject was discontinued from the study.

If any pregnancy occurs during the course of the study, then the investigator or other site personnel will inform the appropriate sponsor representatives within 1 day, ie, immediately but **no later than 24 hours** after becoming aware of the event.

The designated sponsor representative works with the investigator to ensure that all relevant information is provided to the sponsor's patient safety data entry site within 1 or 5 calendar days for SAEs (see Section 5.4) and within 30 days for all other pregnancies.

The same timelines apply when outcome information is available.

The pregnancy reporting module in the eCRF is used to report the pregnancy and the pregnancy outcome module is used to report the outcome of the pregnancy.

Any subject who becomes pregnant during the course of the study will be followed so that pregnancy outcome can be determined and reported to the sponsor and the regulatory authorities.

6 STUDY AND DATA MANAGEMENT

6.1 Training of Study Site Personnel

Before the first subject is entered into the study, a MedImmune representative will review and discuss the requirements of the protocol and related documents with the investigational staff and also train them in any study-specific procedures and system(s) utilised.

The Principal Investigator will ensure that appropriate training relevant to the study is given to all of these staff, and that any new information relevant to the performance of this study is forwarded to the staff involved.

The Principal Investigator will maintain a record of all individuals involved in the study (medical, nursing, and other staff).

6.2 Monitoring of the Study

During the study, a MedImmune representative will have regular contacts with the study site, including visits to:

- Provide information and support to the investigator(s)
- Confirm that facilities remain acceptable
- Confirm that the investigational team is adhering to the protocol, that data are being accurately and timely recorded in the eCRFs, that biological samples are handled in accordance with the Laboratory Manual and that study drug accountability checks are being performed
- Perform source data verification (a comparison of the data in the eCRFs with the subject's medical records at the hospital or practice, and other records relevant to the study) including verification of informed consent of participating subjects. This will require direct access to all original records for each subject (eg, clinic charts).
- Ensure withdrawal of informed consent to the use of the subject's biological samples is reported and biological samples are identified and disposed of/destroyed accordingly, and the action is documented, and reported to the subject.

The MedImmune representative will be available between visits if the investigator(s) or other staff at the centre needs information and advice about the study conduct.

6.2.1 Source Data

Refer to the Clinical Study Agreement for location of source data.

6.2.2 Study Agreements

The Principal Investigator at each of the centres should comply with all the terms, conditions, and obligations of the Clinical Study Agreement, or equivalent, for this study. In the event of

any inconsistency between this protocol and the Clinical Study Agreement, the terms of protocol shall prevail with respect to the conduct of the study and the treatment of subjects and in all other respects, not relating to study conduct or treatment of subjects, the terms of the Clinical Study Agreement shall prevail.

Agreements between MedImmune and the Principal Investigator must be in place before any study-related procedures can take place, or subjects are enrolled.

6.2.3 Archiving of Study Documents

The investigator follows the principles outlined in the Clinical Study Agreement.

6.3 Study Timetable and End of Study

An individual subject will be considered to have completed the study if the subject was followed through to their last protocol-specified visit/assessment, regardless of the number of doses of investigational product that was received.

Subjects will be considered not to have completed the study if consent was withdrawn or the subject was lost to follow-up (see Section 4.1.5 and Section 4.1.6).

The end of the study ("study completion") is defined as the date of the last protocol-specified visit/assessment for the last subject in the study.

6.4 Data Management

Data management will be performed by MedImmune Data Management staff according to the Data Management Plan.

An electronic data capture system will be used for data collection and query handling. The investigator will ensure that data are recorded in the eCRFs as specified in the study protocol and in accordance with the eCRF instructions provided.

The investigator ensures the accuracy, completeness, and timeliness of the data recorded and of the provision of answers to data queries according to the Clinical Study Agreement. The investigator will sign the completed eCRFs. A copy of the completed eCRFs will be archived at the study site.

6.5 Medical Monitor Coverage

Each subject will be provided with contact information for the principal investigator. In addition, each subject will receive a toll-free number intended to provide the subject's physician access to a medical monitor 24 hours a day, 7 days a week in the event of an emergent situation where the subject's health is deemed to be at risk. In this situation, when a subject presents to a medical facility where the treating physician or health care provider

requires access to a physician who has knowledge of the investigational product and the clinical study protocol and the principal investigator is not available, the treating physician or health care provider can contact a medical monitor through this system, which is managed by a third party vendor.

7 ETHICAL AND REGULATORY REQUIREMENTS

7.1 Subject Data Protection

Each subject will be assigned a SID to ensure that personally identifiable information is kept separate from the study data. Subject data that are relevant to the trial, eg, demographic information, physical or mental health condition, diagnosis, comorbidities, laboratory test results, etc. will only be collected with the subject's informed consent. The Informed Consent Form will incorporate (or, in some cases, be accompanied by a separate document incorporating) wording that describes how subject data will be collected, used, and distributed in compliance with relevant data protection and privacy legislation.

Extra precautions will be taken to preserve confidentiality and prevent genetic data being linked to the identity of the subject. MedImmune will not provide individual genotype results to subjects, any insurance company, any employer, their family members, general physician or any other third party, unless required to do so by law.

7.2 Ethics and Regulatory Review

The IEC responsible for each site must review and approve the final study protocol, including the final version of the informed consent form (ICF) and any other written information and/or materials to be provided to the subjects. The IEC must also approve all advertising used to recruit subjects for the study. The investigator is responsible for submitting these documents to the applicable IEC and distributing them to the study site staff.

The opinion of the IEC must be given in writing. The investigator must provide a copy of the written approval to MedImmune before enrolment of any subject into the study.

The IEC should approve all advertising used to recruit subjects for the study.

MedImmune should approve any modifications to the ICF that are needed to meet local requirements.

If required by local regulations, the protocol must be re-approved by the IEC annually.

Before the study is initiated, MedImmune will ensure that the national regulatory authority in each country has been notified and their approval has been obtained, as required. MedImmune will provide safety updates/reports according to local requirements, including suspected

unexpected serious adverse reactions where relevant, to regulatory authorities, IEC, and principal investigators.

Each principal investigator is responsible for providing reports of any serious and unexpected adverse drug reactions from any other study conducted with the investigational product to the IEC. MedImmune will provide this information to the principal investigator so that he/she can meet these reporting requirements.

7.3 Informed Consent

Informed consent of each subject will be obtained through a written and verbal explanation process that addresses all elements required by ICH/GCP. MedImmune will develop a core ICF for use by all investigators in the clinical study. MedImmune must approve any modifications to the ICF that are needed to meet local requirements.

The principal investigator(s) at each centre will:

- Ensure each subject is given full and adequate oral and written information about the nature, purpose, possible risk, and benefit of the study
- Ensure each subject is notified that they are free to discontinue from the study at any time
- Ensure that each subject is given the opportunity to ask questions and allowed time to consider the information provided
- Ensure each subject provides signed and dated informed consent before conducting any procedure specifically for the study
- Ensure the original, signed ICF(s) is/are stored in the Investigator's Study File
- Ensure a copy of the signed ICF is given to the subject
- Ensure that any incentives for subjects who participate in the study as well as any provisions for subjects harmed as a consequence of study participation are described in the ICF that is approved by an IEC

7.4 Changes to the Protocol and Informed Consent Form

Study procedures will not be changed without the mutual agreement of the principal investigator and MedImmune.

Substantial changes must be documented in a study protocol amendment. MedImmune will distribute amended versions of the protocol to the principle investigator(s). Before implementation, amended protocols must be approved by relevant IEC (see Section 7.2) and according to local requirements, the national regulatory authority approval. The IEC must also approve revisions to the ICF, advertising, and any other written information and/or materials resulting from the change to the protocol.

If local regulations require, any unsubstantial changes will be communicated to or approved by each IEC.

7.5 Audits and Inspections

Authorised representatives of MedImmune, a regulatory authority, or an IEC may perform audits or inspections at the centre, including source data verification. The purpose of an audit or inspection is to systematically and independently examine all study-related activities and documents, to determine whether these activities were conducted, and data were recorded, analysed, and accurately reported according to the protocol, GCP, guidelines of the ICH, and any applicable regulatory requirements. The investigator will contact MedImmune immediately if contacted by a regulatory agency about an inspection at the site.

8 REFERENCES

Bagger et al, 2015
Bagger JI, Holst JJ, Hartmann B, Andersen B, Knop FK, Vilsbøll T. Effect of oxyntomodulin, glucagon, GLP-1, and combined glucagon +GLP-1 infusion on food intake, appetite, and resting energy expenditure. J Clin Endocrinol Metab. 2015;100(12):4541-52.

Cherrington et al, 1981
Cherrington AD, Williams PE, Shulman GI, Lacy WW. Differential time course of glucagon's effect on glycogenolysis and gluconeogenesis in the conscious dog. Diabetes. 1981 Mar;30(3):180-7.

Habegger et al, 2013
Habegger KM, Stemmer K, Cheng C, Müller TD, Heppner KM, Ottaway N, et al. Fibroblast growth factor 21 mediates specific glucagon actions. Diabetes. 2013;62(5):1453-63.

Krssak et al, 2004
Krssak M, Brehm A, Bernroider E, Anderwald C, Nowotny P, Dalla Man C, et al. Alterations in postprandial hepatic glycogen metabolism in type 2 diabetes. Diabetes. 2004 Dec;53(12):3048-56.

Landau et al, 1995
Landau BR, Wahren J, Chandramouli V, Schumann WC, Ekberg K, Kalhan SC. Use of ^{2}H2O for estimating rates of gluconeogenesis. Application to the fasted state. J Clin Invest. 1995 Jan;95(1):172-8.

Leung et al, 1986
Leung NW, Farrant P, and Peters TJ. Liver volume measurement by ultrasonography in normal subjects and alcoholic patients. J Hepatol. 1986;2(2):157-64.

Lynch et al 2014
Lynch AM, Pathak N, Pathak V, O'Harte FP, Flatt PR, Irwin N, et al. A novel DPP IV-resistant C-terminally extended glucagon analogue exhibits weight-lowering and diabetes-protective effects in high-fat-fed mice mediated through glucagon and GLP-1 receptor activation. Diabetologia. 2014;57(9):1927-36.

Magnusson et al, 1992
Magnusson I, Rothman DL, Katz LD, Shulman RG, and Shulman GI. Increased rate of gluconeogenesis in type II diabetes mellitus. A 13C nuclear magnetic resonance study. J Clin Invest. 1992 Oct;90(4):1323-7.

Moore et al, 2017
Moore MC, Smith MS, Farmer B, Kraft G, Shiota M, Williams PE, Cherrington AD. Priming Effect of a Morning Meal on Hepatic Glucose Disposition Later in the Day. Diabetes. 2017 May;66(5):1136-1145.

Petersen et al, 2005
Petersen KF, Dufour S, Befroy D, Lehrke M, Hendler RE, Shulman GI. Reversal of non-alcoholic hepatic steatosis, hepatic insulin resistance, and hyperglycemia by moderate weight reduction in patients with type 2 diabetes. Diabetes. 2005 Mar;54(3):603-8.

Rothman et al, 1991
Rothman DL, Magnusson I, Kat LD, Shulman RG, Shulman GI. Quantitation of hepatic glycogenolysis and gluconeogenesis in fasting humans with 13C NMR. Science. 1991 Oct 25;254(5031):573-6.

Savage et al, 2007
Savage DB, Petersen KF, Shulman GI. Disordered Lipid Metabolism and the Pathogenesis of Insulin Resistance. Physiol Rev. 2007 Apr;87(2):507-20.

Skyder et al, 2017
Skyder JS, Bakris GL, Bonifacio E, Darsow T, Eckel RH, Groop L, et al. Differentiation of diabetes by pathophysiology, natural history, and prognosis. Diabetes. 2017 Feb;66(2):241-55.

Uhlén et al, 2015
Uhlén M1, Fagerberg L, Hallström BM, Lindskog C, Oksvold P, Mardinoglu A, et al. Proteomics. Tissue-based map of the human proteome. Science. 2015 Jan 23;347 (6220): 1260419. doi: 10.1126/science.1260419.

Victoza package insert, 2017
Victoza package insert. Bagsvaerd, DK: Novo Nordisk A/S, 2017. |

Wynne et al, 2006
Wynne K, Park AJ, Small CJ, Meeran K, Ghatei MA, Frost GS. Oxyntomodulin increases energy expenditure in addition to decreasing energy intake in overweight and obese humans: a randomized controlled trial. Int J Obes. 2006;30(12):1729-36.

9 CHANGES TO THE PROTOCOL

9.1 Protocol Amendment 6, 15Jul2020

Various corrections and clarifications have been made to align with the duration of Part B, and a window in which subjects receive standardised solid meals following the mixed-meal tolerance test was revised for consistency due to previous typographical errors. Text was added to ensure the safety of subjects during the coronavirus disease (COVID-19) pandemic, and the provision for anti-drug antibody (ADA) samples was revised, including revision of the ADA titre threshold from 80 to 10 to expand ADA testing. Other minor formatting and editorial changes have also been made, including the deletion of obsolete contact numbers for reporting product complaints and an update of the sponsor representative name and contact information.

Changes to Protocol Amendment 6 are summarised in the table below. All changes have been deemed nonsubstantial.

Section Changed	Key Details of the Amendment
**Amendment 6 / 15Jul2020**	
[redacted]	[redacted]
Section 4.2 (Schedule of Study Procedures)	• Stated that other measures for carrying out protocol-related activities may be required due to the COVID-19 pandemic
Table 9 (Schedule of Treatment Period Study Procedures [Day -4 through Day 22] [Part B]) and Table 10 (Schedule of Treatment Period Study Procedures [Day 32 through Day 36] [Part B])	Corrected various footnotes, including:<br>• Footnote related to standardised meals: Revised window in which subjects receive standardised solid meals following the MMTT for consistency (ie, revised "± 30" to "± 5"on Day -1 for evening meal and Day 35 for lunch and evening meals)<br>• Footnote related to investigational product administration: Deleted text on predose PK sampling for Day 8 (Visit 3) since not applicable<br>• Added MRS abbreviation to footnotes<br>• Footnote related to 5-day metformin washout: Corrected day subjects should stop taking metformin from "Day 30" to "Day 32"<br>• Footnote related to collection of blood samples for non-genetic future research: Revised "4 (± 30 min)" to "220 min (± 5 min)" for consistency<br>• Footnote related to collection of optional stool sample for microbiome research: Corrected

Section Changed	Key Details of the Amendment
	“from Day 33 to Day 36” to “from Day 34 to Day 36”
Table 11 (Schedule of Follow-up Procedures)	• Revised immunogenicity (ADA) footnote to specify that subjects with an ADA titre ≥ 10 at the follow-up visit will be asked to return to provide another ADA sample at 3-month intervals until the titre is < 10
Section 4.3.1.1 (Mixed-meal Tolerance Tests), Section 4.3.4 (Pharmacodynamic Evaluation and Measures), Section 4.3.7 (Biomarker Evaluation and Methods), and Section 5.3.1 (Time Period for Collection of Adverse Events)	• Specified 35-day treatment length and/or Part A versus Part B time points as needed throughout for clarification
Section 4.5.1 (Identity of Investigational Product[s])	• Corrected number of days liraglutide to be administered for Part B of the study
Section 4.5.1.5 (Reporting Product Complaints)	• Deleted an obsolete phone number and fax number
Section 10.1 (Appendix 1 – Signatures)	• Updated the sponsor representative name and contact information

ADA = anti-drug antibody; COVID-19 = coronavirus disease; MMTT = mixed-meal tolerance test; MRS = magnetic resonance spectroscopy; PK = pharmacokinetic

9.2 Protocol Amendment 5, 03May2019

Based on the analysis of data from Part A of this study, changes to Part B have been made to improve tolerability while still evaluating efficacy. The introduction of a multi-dose pen device to administer the investigational product facilitated the introduction of 50 µg starting dose but this has had an impact on the duration of dosing. Formatting changes have also been made in line with the current process of the sponsor.

Changes relating to Part B of the study have been made in Protocol Amendment 5 and are summarized below. Other minor editorial changes have also been made.

Section Changed	Key Details of the Amendment
**Amendment 5 / 03May2019**	
Section 1.7 (Research Hypothesis), Section 2.1 (Primary Objective and Associated Endpoints), Section 2.2 (Secondary Objective and Associated Endpoints), Section 2.3 (Exploratory Objective and Associated Endpoints), Section 3.2.3.1 (Primary Endpoint),	• Change in the primary endpoint to % change in fasting glycogen (at T = 24 hrs time point)<br>• Change in MRS schedule from 0, 4, 9, 14, and 24 hrs to 0, 5, 14 and 24 hrs<br>• Addition of analysis of endpoints are Day 35 for Part B
Section 3.1.1 (Overview), Section 3.1.2 (Treatment), Section 3.1.3 (Dose Escalation), Table 1, Section	• The dosing schedule for MEDI0382 will now commence at a lower start dose of 50 µg and will be uptitrated in 7-day intervals to 300 µg

Section Changed	Key Details of the Amendment
3.2.3.3 (Exploratory Endpoints), Section 4.5 (Investigational Products), Section 4.8.3 (Efficacy)	across 35 days; the end of treatment assessments have been altered to reflect this.<br>• The delivery device for MEDI0382 will be a pen device rather than PFS<br>• Introduction of a 14 hour fast prior to measurement of the scans related to the primary endpoint<br>• Alteration to visits; one less outpatient visit and two additional remote contacts to guide dose titration and collect AEs/SAEs.
Section 3.1.1 (Overview), Section 4.1.1 (Number of Subjects), Section 4.8.2 (Sample Size)	• The number of subjects for Part B has increase to 30 (maximum 35 subjects)
Table 9 (Schedule of Treatment Period Study Procedures (Part B), Table 10 (Schedule of Treatment Period Study Procedures (Part B), Section 4.3.5 (Pharmacokinetic Evaluation and Methods)	• Tables were added<br>• On Day 8, collection of laboratory assessments and predose PK and clarification of footnotes.<br>• Day 15 is now converted to a remote contact
Section 3.1.1 (Overview), Section 4.3.1.1 (Mixed-meal Tolerance Test)	• Removal of optional C13 glucose administration
Section 4.5.1.3 (At Home Administration)	• Instructions specific to the use of the pen were added
Section 4.6.2 (Methods to Ensure Blinding)	• Blinding instructions for the pen were added.
Section 10.5 (Appendix 5 - Actions Required in Cases of Increases in Liver Biochemistry and Evaluation of Hy's Law)	• Update of Hy's Law monitoring in line with Sponsots current practice
Section 10.9 (Appendix 9 – Summary of Potential Risks)	• This appendix has been removed as the current version of the IB should be referenced for this information and hold the most up to date summary of potential risks.

AE = adverse event; hrs = hours; IB = Investigator's Brochure; MRS = magnetic resonance spectroscopy, PFS = pre-filled syringe; PK = pharmacokinetics; SAE = serious adverse event; T = time

9.3 Protocol Amendment 4, 24Sep2018

Changes have been made in Protocol Amendment 4. Major changes are summarised below. Other minor editorial changes have also been made.

Key Details of Amendment	Reason for Amendment
**Amendment 4 / 24Sep2018**	
Synopsis, Section 3.1.1, 3.1.2, and 4.1.1<br>Increased subject numbers	The number of subjects to be randomised in Part A was increased from 16 to 20 in order to ensure that at least 16 subjects complete the study. The total number of subjects in Part A and Part B therefore also increased to 44.

Key Details of Amendment	Reason for Amendment
Tables 4.2.2-1 and 4.2.2-2<br>Correction of typographic errors	Correction of error message for the footnote reference on the Serial MRS scans row. Footnote reference "m" added.<br>Correction of timeframe for MMTT procedure and administration of standard liquid meal for this test so that footnotes match.
Section 10.7 (Appendix 7)<br>Added clarity	Updates to wording about sample storage as some samples will be stored at TS Biorepository and others at AZ Biobank in Gothenburg (due to long term storage requirements in Sweden)
Section 10.8 (Appendix 8)<br>Added clarity	Modified to include only Standardised Meals, Deuterated water, MMTTs, and Serial MRS Scans (removed blood samples except for those taken along with the MMTT)

AZ = AstraZeneca; MMTT = mixed-meal tolerance test; MRS = magnetic resonance spectroscopy; TS = Translational Sciences

9.4 Protocol Amendment 3, 08May2018

Key Details of Amendment	Reason for Amendment
**Amendment 3 / 08May2018**	
Protocol Synopsis, Section 3.1.1, Table 4.2.2-1 and footnote p to update randomisation time to occur on Day -1 or Day 1	Text was amended to provide clarity.
Protocol Synopsis and Section 4.8.7 to reflect Part A interim analysis will be done after last subject last visit	Text was amended to provide clarity.
Section 4.3.4, revised to reflect that 70% 2H2O will be used instead of 100% 2H2O and calculations adjusted accordingly	Text was amended to provide clarity.
Tables 4.2.2-1 (footnote g), added to reflect that subjects assigned to liraglutide do not need to undergo blood tests for ADAs	Text was amended to provide clarity.
Table 4.2.2-2 (footnotes e and n added) to reflect that subjects assigned to liraglutide do not need to undergo blood tests for ADAs, and to reinforce that investigational product (MEDI0382 or placebo) will be dispensed on Day 26	Text was amended to provide clarity.
Table 4.2.3-1 (footnote b revised) to reflect that subjects assigned to liraglutide do not need to undergo blood tests for ADAs	Text was amended to provide clarity.

Key Details of Amendment	Reason for Amendment
Section 4.3.2.3 to reflect lead II may also be used for ECG analysis	Text was amended to provide clarity.
Section 3.2.1 and Section 4.8.2	Typographic corrections (ie, missing D for D Code corrected and addition of 0 to 0.1, respectively)
Section 4.5.1 to remove reference to a take home carton. Syringes are pre-packaged into cartons with 8 syringes.	Text was amended to provide clarity.
Section 4.8.7, revised to: An interim analysis after the last subject 'completes their last visit'	Text was amended to provide clarity.

9.5 Protocol Amendment 2, 29Mar2018

Key Details of Amendment	Reason for Amendment
**Amendment 2 / 29Mar2018**	
Synopsis and Sections 3.1.1, 3.2.3.1 and 4.3.1.1 and corresponding footnote k in table 4.2.2.1 and footnotes h and j in 4.2.2.2 have been updated to reflect that co-administration of C-13 labelled glucose to augment the MRS signal may not be required and is therefore now an optional component to this study.	Update to make a procedure that was previously a core part of the main study into an optional assessment.

9.6 Protocol Amendment 1, 27Mar2018

Changes have been made in Protocol Amendment 1 at the request of the Swedish Medical Products Agency. A summary of these changes is provided below.

Key Details of Amendment	Reason for Amendment
**Amendment 1 / 27Mar2018**	
Section 1.6 and Appendix 9, more detail has been added regarding the benefit risk associated with the study.	At the request of the Swedish Medical Products Agency.
Section 4.1.3 Exclusion criterion 3 and Section 4.7.2 have been modified to ensure subjects who take warfarin are not enrolled and to clarify that warfarin is a prohibited concomitant medication	
Sections 4.1.6 and 4.4 have been modified to clarify that study suspension applies to both Parts A and B	
Synopsis and Section 4.8.1.1 have been modified to specify that the efficacy analyses will be performed using an ITT population (according to the randomised treatment).	

ITT = intent=to-treat

10 APPENDICES

10.1 Appendix 1 - Signatures

Sponsor Signature(s)

An Exploratory Phase 2, Randomised, Double-Blind, Placebo-Controlled and Open-Label Active Comparator Study to Evaluate the Effect of MEDI0382 on Hepatic Glycogen Metabolism in Overweight and Obese Subjects with Type 2 Diabetes Mellitus

I agree to the terms of this protocol.

Signature and date: Electronic signature attached

[redacted]

[redacted]

[redacted]

[redacted]

10.2 Appendix 2 – Contraception Guidance

For females of childbearing potential:

- Females of childbearing potential are defined as those who are not surgically sterile (ie, surgical sterilisation includes bilateral tubal ligation, bilateral oophorectomy, or complete hysterectomy) or those who are not postmenopausal (defined as at least 1 year since last menses and/or having an elevated follicle-stimulating hormone level in the postmenopausal range in previous laboratory test results).
- A highly effective method of contraception is defined as one that results in a low failure rate (ie, less than 1% per year) when used consistently and correctly. The acceptable methods of contraception are described in Table 13.
- Female subjects must refrain from egg cell donation and breastfeeding while on study and for 28 days after the final dose of investigational product.

Table 13 Highly Effective Methods of Contraception

• Tubal occlusion<br>• Copper T intrauterine device<br>• Levonorgestrel-releasing intrauterine system (eg, Mirena®)<br>• Medroxyprogesterone injections (eg, Depo-Provera®)<br>• Etonogestrel implants (eg, Implanon®, Norplan®)<br>• Norelgestromin/ethinyl estradiol transdermal system<br>• Intravaginal device (eg, NuvaRing®)

10.3 Appendix 3 - Additional Safety Guidance

Further Guidance on the Definition of a Serious Adverse Event (SAE)

Life threatening

'Life-threatening' means that the subject was at immediate risk of death from AE as it occurred or it is suspected that use or continued use of the product would result in the subject's death. 'Life-threatening' does not mean that had an AE occurred in a more severe form it might have caused death (eg, hepatitis that resolved without hepatic failure).

Hospitalisation

Outpatient treatment in an emergency room is not in itself a serious AE, although the reasons for it may be (eg, bronchospasm, laryngeal oedema). Hospital admissions and/or surgical operations planned before or during a study are not considered AEs if the illness or disease existed before the subject was enrolled in the study, provided that it did not deteriorate in an unexpected way during the study.

Important Medical Event or Medical Intervention

Medical and scientific judgment should be exercised in deciding whether a case is serious in situations where important medical events may not be immediately life threatening or result in

death, hospitalisation, disability or incapacity but may jeopardize the subject or may require medical intervention to prevent one or more outcomes listed in the definition of serious. These should usually be considered as serious.

Simply stopping the suspect drug does not mean that it is an important medical event; medical judgment must be used.

Examples of such events are:

- Angioedema not severe enough to require intubation but requiring intravenous hydrocortisone treatment
- Hepatotoxicity caused by paracetamol (acetaminophen) overdose requiring treatment with N-acetylcysteine
- Intensive treatment in an emergency room or at home for allergic bronchospasm
- Blood dyscrasias (eg, neutropenia or anaemia requiring blood transfusion) or convulsions that do not result in hospitalisation
- Development of drug dependency or drug abuse

Assessment of Severity

Assessment of severity is one of the responsibilities of the investigator in the evaluation of AEs and SAEs. The determination of severity should be made by the investigator based upon medical judgment and the severity categories of Grade 1 to 5 as defined below.

Grade 1	An event of mild intensity that is usually transient and may require only minimal treatment or therapeutic intervention. The event does not generally interfere with usual activities of daily living.
Grade 2	An event of moderate intensity that is usually alleviated with additional specific therapeutic intervention. The event interferes with usual activities of daily living, causing discomfort but poses no significant or permanent risk of harm to the subject.
Grade 3	A severe event that requires intensive therapeutic intervention. The event interrupts usual activities of daily living, or significantly affects the clinical status of the subject.
Grade 4	An event, and/or its immediate sequelae, that is associated with an imminent risk of death or with physical or mental disabilities that affect or limit the ability of the subject to perform activities of daily living (eating, ambulation, toileting, etc).

Grade 5 Death as a result of an event.

It is important to distinguish between serious criteria and severity of an AE. Severity is a measure of intensity whereas seriousness is defined by the criteria in Section 5.2. A Grade 3 AE need not necessarily be considered an SAE. For example, a Grade 3 headache that persists for several hours may not meet the regulatory definition of an SAE and would be considered a nonserious event, whereas a Grade 2 seizure resulting in a hospital admission would be considered an SAE.

Assessment of Relationship

Relationship to Investigational Product

The investigator is required to provide an assessment of relationship of AEs and SAEs to the investigational product.

An event will be considered "not related" to use of the investigational product if any of the following tests are met:

- An unreasonable temporal relationship between administration of the investigational product and the onset of the event (eg, the event occurred either before, or too long after, administration of the investigational product for it to be considered product-related)
- A causal relationship between the investigational product and the event is biologically implausible (eg, death as a passenger in an automobile accident)
- A clearly more likely alternative explanation for the event is present (eg, typical adverse reaction to a concomitant drug and/or typical disease-related event)

Individual AE/SAE reports will be considered "related" to use of the investigational product if the "not related" criteria are not met.

"Related" implies that the event is considered to be "associated with the use of the drug" meaning that there is "a reasonable possibility" that the event may have been caused by the product under investigation (ie, there are facts, evidence, or arguments to suggest possible causation).

Relationship to Protocol Procedures

The investigator is also required to provide an assessment of relationship of SAEs to protocol procedures on the SAE Report Form. This includes nontreatment-emergent SAEs (ie, SAEs that occur prior to the administration of investigational product) as well as treatment-emergent SAEs. A protocol-related SAE may occur as a result of a procedure or intervention required during the study (eg, blood collection, washout of an existing medication). The following guidelines should be used by investigators to assess the relationship of SAEs to the protocol:

Protocol related:	The event occurred due to a procedure/intervention that was described in the protocol for which there is no alternative etiology present in the subject's medical record.
Not protocol related:	The event is related to an etiology other than the procedure/ intervention that was described in the protocol (the alternative etiology must be documented in the study subject's medical record).

10.4 Appendix 4 - National Institute of Allergy and Infectious Disease and Food Allergy and Anaphylaxis Network Guidance for Anaphylaxis Diagnosis

Sampson HA, Muñoz-Furlong A, Campbell RL, Adkinson FN Jr, Bock SA, Branum A, et al. Second symposium on the definition and management of anaphylaxis: Summary report -- Second National Institute of Allergy and Infectious Disease/Food Allergy and Anaphylaxis Network symposium. J Allergy Clin Immunol. 2006;117:391-7.

National Institute of Allergy and Infectious Disease (NAID) and Food Allergy and Anaphylaxis Network (FAAN) define anaphylaxis as a serious allergic reaction that is rapid in onset and may cause death. They recognize 3 categories of anaphylaxis, with criteria designated to capture from 80% of cases (category 1) to > 95% of all cases of anaphylaxis (for all 3 categories).

1 Acute onset of an illness (minutes to several hours) with involvement of the skin, mucosal tissue, or both (eg, generalised hives, pruritus or flushing, swollen lips-tongue-uvula) AND AT LEAST ONE OF THE FOLLOWING
   (a) Respiratory compromise (eg, dyspnoea, wheeze-bronchospasm, stridor, reduced peak expiratory flow (PEF), hypoxaemia)
   (b) Reduced BP or associated symptoms of end-organ dysfunction (eg, hypotonia [collapse], syncope, incontinence)
2 Two or more of the following that occur rapidly after exposure to a likely allergen for that patient (minutes to several hours):
   (a) Involvement of the skin-mucosal tissue (eg, generalised hives, itch-flush, swollen lips-tongue-uvula)
   (b) Respiratory compromise (eg, dyspnoea, wheeze-bronchospasm, stridor, reduced PEF, hypoxaemia)
   (c) Reduced BP or associated symptoms (eg, hypotonia [collapse], syncope, incontinence)
   (d) Persistent gastrointestinal symptoms (eg, crampy abdominal pain, vomiting)
3 Reduced BP after exposure to known allergen for that patient (minutes to several hours):
   (a) Infants and children: low systolic BP (age specific) or greater than 30% decrease in systolic BP
   (b) Adults: systolic BP of less than 90 mm Hg or greater than 30% decrease from that person’s baseline

10.5 Appendix 5 - Actions Required in Cases of Increases in Liver Biochemistry and Evaluation of Hy’s Law

10.5.1 Introduction

This appendix describes the process to be followed in order to identify and appropriately report potential Hy’s Law cases and Hy’s Law cases. It is not intended to be a comprehensive guide to the management of elevated liver biochemistries.

During the course of the study the investigator will remain vigilant for increases in liver biochemistry. The investigator is responsible for determining whether a subject meets potential Hy’s Law (PHL) criteria at any point during the study.

All sources of laboratory data are appropriate for the determination of PHL and HL events; this includes samples taken at scheduled study visits and other visits including central and all local laboratory evaluations even if collected outside of the study visits; for example, PHL criteria could be met by an elevated ALT from a central laboratory **and/or** elevated TBL from a local laboratory.

The investigator will also review AE data (for example, for AEs that may indicate elevations in liver biochemistry) for possible potential Hy’s Law events.

The investigator participates, together with MedImmune clinical project representatives, in review and assessment of cases meeting PHL criteria to agree whether Hy’s Law (HL) criteria are met. HL criteria are met if there is no alternative explanation for the elevations in liver biochemistry other than Drug Induced Liver Injury (DILI) caused by the investigational product.

The investigator is responsible for recording data pertaining to PHL/HL cases and for reporting adverse events (AE) and serious adverse events (SAE) according to the outcome of the review and assessment in line with standard safety reporting processes.

10.5.2 Definitions

10.5.2.1 Potential Hy’s Law

AST or ALT $\geq 3 \times$ ULN **together with** TBL $\geq 2 \times$ ULN at any point during the study following the start of study medication irrespective of an increase in alkaline phosphatase.

10.5.2.2 Hy’s Law

AST or ALT $\geq 3 \times$ ULN together with total bilirubin (TBL) $\geq 2 \times$ ULN at any point during the study following the start of investigational product irrespective of an increase in alkaline phosphatase.

For potential Hy's Law and Hy's Law, the elevation in transaminases must precede or be coincident with (ie, on the same day) the elevation in TBL, but there is no specified timeframe within which the elevations in transaminases and TBL must occur.

10.5.3 Identification of Potential Hy's Law Cases

In order to identify cases of PHL it is important to perform a comprehensive review of laboratory data for any subject who meets any of the following identification criteria in isolation or in combination:

- ALT ≥ 3 × ULN
- AST ≥ 3 × ULN
- TBL ≥ 2 × ULN

The investigator will, without delay, review each new laboratory report and if the identification criteria are met will:

- Notify the sponsor study representative
- Determine whether the subject meets potential Hy's Law criteria (see Section 10.5.2.2) by reviewing laboratory reports from all previous visits
- Promptly enter the laboratory data into the laboratory CRF

10.5.4 Follow-up

10.5.4.1 Potential Hy's Law Criteria Are Not Met

If the subject does not meet potential Hy's Law criteria the investigator will:

- Inform the study representative that the subject has not met potential Hy's Law criteria
- Perform follow-up on subsequent laboratory results according to the guidance provided in the Clinical Study Protocol.

10.5.4.2 Potential Hy's Law Criteria Are Met

If the subject does meet potential Hy's Law criteria the investigator will:

- Determine whether potential Hy's Law criteria were met at any study visit prior to starting study treatment
- Notify the sponsor study representative who wil then inform the study team.
- Within 1 day of potential Hy's Law criteria being met, the investigator will report the case as an SAE of Potential Hy's Law; serious criteria 'Important medical event' and causality assessment 'yes/related' according to clinical study protocol process for SAE reporting

The medical monitor contacts the investigator, to provide guidance, discuss and agree on an approach for the study subjects' follow-up (including any further laboratory testing) and the continuous review of data.

Subsequent to this contact the investigator will:

- Monitor the subject until liver biochemistry parameters and appropriate clinical symptoms and signs return to normal or baseline levels, or as long as medically indicated. Complete follow-up SAE form as required.
- Investigate the etiology of the event and perform diagnostic investigations as discussed with the medical monitor
- Complete the relevant eCRF Modules as information becomes available

10.5.5 Review and Assessment of Potential Hy's Law Cases

The instructions in this section should be followed for all cases where potential Hy's Law criteria are met.

As soon as possible after the biochemistry abnormality was initially detected, the medical monitor will contact the investigator in order to review available data and agree on whether there is an alternative explanation for meeting potential Hy's Law criteria other than DILI caused by the investigational product, to ensure timely analysis and reporting to health authorities per local requirements from the date potential Hy's Law criteria were met. The medical monitor and Global Safety Physician will also be involved in this review together with other subject matter experts as appropriate.

According to the outcome of the review and assessment, the investigator will follow the instructions below.

Where there is an agreed alternative explanation for the ALT or AST and TBL elevations, a determination of whether the alternative explanation is an AE will be made and subsequently whether the AE meets the criteria for a SAE:

- If the alternative explanation is **not** an AE, record the alternative explanation on the appropriate CRF
- If the alternative explanation is an AE/SAE, update the previously submitted potential Hy's Law SAE and AE CRFs accordingly with the new information (reassessing event term; causality and seriousness criteria) following sponsor's standard processes

If it is agreed that there is **no** explanation that would explain the ALT or AST and TBL elevations other than the investigational product:

- Send the updated SAE (report term 'Hy's Law') according to the sponsor's standard processes.
- The 'Medically Important' serious criterion should be used if no other serious criteria apply
- As there is no alternative explanation for the Hy's Law case, a causality assessment of 'related' should be assigned

If, there is an unavoidable delay of over 15 calendar days in obtaining the information necessary to assess whether or not the case meets the criteria for Hy's Law, then it is assumed that there is no alternative explanation until such time as an informed decision can be made:

- Provide any further update to the previously submitted SAE of Potential Hy's Law (report term now 'Hy's Law case'), ensuring causality assessment are related to the investigational product and seriousness criteria is medically important, according to the clinical study protocol process for SAE reporting
- Continue follow-up and review according to agreed plan. Once the necessary supplementary information is obtained, repeat the review and assessment to determine whether Hy's Law criteria are still met. Update the previously submitted potential Hy's Law SAE report following clinical study protocol process for SAE reporting, according to the outcome of the review and amend the reported term if an alternative explanation for the liver biochemistry elevations is determined

10.5.6 Laboratory Tests

The list below represents the standard, comprehensive list of follow-up tests which are recommended but not mandatory when using a central laboratory. For studies using a local laboratory, the list may be modified based on clinical judgement. If required, for additional assistance on which tests could be used to evaluate other potential causes of liver dysfunction, consult with the Hepatic Safety Knowledge Group. Any test results need to be recorded.

Hy's Law lab kit for central laboratories

Additional standard chemistry and coagulation tests	GGT<br>LDH<br>Prothrombin time<br>INR
Viral hepatitis	IgM anti-HAV<br>IgM and IgG anti-HBc<br>HBsAg<br>HBV DNA<br>IgM and IgG anti-HCV<br>HCV RNA<br>IgM anti-HEV<br>HEV RNA
Other viral infections	IgM & IgG anti-CMV<br>IgM & IgG anti-HSV<br>IgM & IgG anti-EBV
Alcoholic hepatitis	Carbohydrate deficient transferrin (CD-transferrin)
Autoimmune hepatitis	Antinuclear antibody (ANA)<br>Anti-Liver/Kidney Microsomal Ab (Anti-LKM)<br>Anti-Smooth Muscle Ab (ASMA)
Metabolic diseases	alpha-1-antitrypsin<br>Ceruloplasmin<br>Iron<br>Ferritin<br>Transferrin<br>Transferrin saturation

10.5.7 References

Aithal et al 2011, Clinical Pharmacology and Therapeutics 89(6):806-815.

FDA Guidance for Industry (issued July 2009) 'Drug-induced liver injury: Premarketing clinical evaluation'

10.6 Appendix 6 - Genetic Research

Rationale and Objectives

MedImmune intends to collect and store DNA for genetic research to explore how genetic variations may affect clinical parameters, risk and prognosis of diseases, and the response to medications. Genetic research may lead to better understanding of diseases, better diagnosis of diseases or other improvements in health care and to the discovery of new diagnostics, treatments or medications. Genetic research samples may be used to perform whole genome and whole exome sequencing.

In addition, collection of DNA samples from populations with well described clinical characteristics may lead to improvements in the design and interpretation of clinical trials and, possibly, to genetically guided treatment strategies.

Genetic Research Plan and Procedures

Selection of genetic research population and study selection record

All subjects will be asked to participate in this genetic research. Participation is voluntary and if a subject declines to participate there will be no penalty or loss of benefit. The subject will not be excluded from any aspect of the main study.

Inclusion Criteria

For inclusion in this genetic research, subjects must fulfill all of the inclusion criteria described in the main body of the Clinical Study Protocol **and** provide informed consent for the genetic sampling and analyses.

Exclusion Criteria

Exclusion from this genetic research may be for any of the exclusion criteria specified in the main study or any of the following:

- Previous allogeneic bone marrow transplant
- Non-leukocyte depleted whole blood transfusion in 120 days of genetic sample collection

Discontinuation of Subjects from this Genetic Research

Specific reasons for discontinuing a subject from this genetic research are:

Withdrawal of consent for genetic research: Subjects may withdraw from this genetic research at any time, independent of any decision concerning participation in other aspects of the main study. Voluntary discontinuation will not prejudice further treatment. Procedures for discontinuation are outlined in Section 4.1.6 of the main Protocol.

Collection of Samples for Genetic Research

The blood sample for genetic research will be obtained from the subjects at Visit 2 provided that the subject has given explicit consent for this. Although DNA variants are a stable parameter, early sample collection is preferred to avoid introducing bias through excluding subjects who may withdraw due to an AE, such subjects would be important to include in any genetic analysis. If for any reason the sample is not collected at Visit 2, it may be taken at any visit until the last study visit. Only one sample should be collected per subject for genetic research during the study. Samples will be collected, labelled, stored and shipped as detailed in the Laboratory Manual.

Coding and Storage of DNA Samples

The processes adopted for the coding and storage of samples for genetic analysis are important to maintain subject confidentiality. Samples will be stored for a maximum of *15 years*, from the date of last subject last visit, after which they will be destroyed. DNA is a finite resource that is used up during analyses. Samples will be stored and used until no further analyses are possible or the maximum storage time has been reached.

An additional second code will be assigned to the blood either before or at the time of DNA extraction replacing the information on the sample tube. Thereafter, the sample will be identifiable by the second, unique number only. This number is used to identify the sample and corresponding data at the MedImmune genetics laboratories, or at the designated organisation. No personal details identifying the individual will be available to any person (MedImmune employee or designated organisations working with the DNA).

The link between the subject randomisation code and the second number will be maintained and stored in a secure environment, with restricted access at MedImmune or designated organisations. The link will be used to identify the relevant DNA samples for analysis, facilitate correlation of genotypic results with clinical data, allow regulatory audit, and trace samples for destruction in the case of withdrawal of consent.

Ethical and Regulatory Requirements

The principles for ethical and regulatory requirements for the study, including this genetics research component, are outlined in Section 7.2 of the main Protocol.

Informed Consent

The genetic component of this study is optional and the subject may participate in other components of the main study without participating in the genetic component. To participate in the genetic component of the study the subject must sign and date both the consent form for the main study and the genetic component of the study. Copies of both signed and dated consent forms must be given to the subject and the original filed at the study centre. The principal investigator(s) is responsible for ensuring that consent is given freely and that the

subject understands that they may freely discontinue from the genetic aspect of the study at any time.

Subject Data Protection

MedImmune will not provide individual genotype results to subjects, any insurance company, any employer, their family members, or general physician unless required to do so by law.

Extra precautions are taken to preserve confidentiality and prevent genetic data being linked to the identity of the subject. In exceptional circumstances, however, certain individuals might see both the genetic data and the personal identifiers of a subject. For example, in the case of a medical emergency, a MedImmune physician or an investigator might know a subject's identity and also have access to his or her genetic data. Also Regulatory authorities may require access to the relevant files, though the subject's medical information and the genetic files would remain physically separate.

Data Management

Any genotype data generated in this study will be stored at a secure system at MedImmune and/or designated organisations to analyse the samples.

The results from this genetic research may be reported in a separate report from the CSR or published in scientific journals.

MedImmune and its designated organisations may share summary results (such as genetic differences from groups of individuals with a disease) from this genetic research with other researchers, such as Hospitals, Academic Organisations, or Health Insurance Companies. This can be done by placing the results in scientific databases, where they can be combined with the results of similar studies to learn even more about health and disease. The researchers can only use this information for health-related research purposes. Researchers may see summary results but they will not be able to see individual subject data or any personal identifiers.

Some or all of the clinical datasets from the main study may be merged with the genetic data in a suitable secure environment separate from the clinical database.

Statistical Methods and Determination of Sample Size

The number of subjects that will agree to participate in the genetic research is unknown. It is therefore not possible to establish whether sufficient data will be collected to allow a formal statistical evaluation or whether only descriptive statistics will be generated. A Statistical Analysis Plan will be prepared where appropriate.

10.7 Appendix 7 - Biological Samples

Storage, Re-use and Destruction of Biological Samples

Biological samples will be stored for a maximum of 15 years from the date of the Last Subject's Last Visit, after which they will be destroyed.

The results of biomarker and microbiome research will be reported either in the CSR itself or as an addendum, or separately in a scientific report or publication. The results of this research may be pooled with biomarker data from other studies with the study drug to generate hypotheses to be tested in future research. ADA samples will be stored for up to 2 years after marketing approval.

Labelling and Shipment of Biological Samples

The principal investigator ensures that samples are labelled and shipped in accordance with the Laboratory Manual and the Biological Substance, Category B Regulations (materials containing or suspected to contain infectious substances that do not meet Category A criteria).

Any samples identified as Infectious Category A materials are not shipped and no further samples will be taken from the subject unless agreed with MedImmune and appropriate labelling, shipment and containment provisions are approved.

Chain of Custody of Biological Samples

A full chain of custody is maintained for all samples throughout their lifecycle.

The principal investigator at each study site keeps full traceability of collected biological samples from the subjects while in storage at the study site until shipment or disposal (where appropriate) and keeps documentation of receipt of arrival. The sample receiver keeps full traceability of the samples while in storage and during use until used or disposed of or until further shipment and keeps documentation of receipt of arrival.

MedImmune keeps oversight of the entire life cycle through internal procedures, monitoring of study sites and auditing of external laboratory providers.

Stool samples retained for further use are registered in the Translational Sciences Biorepository during the entire life cycle. Blood samples for future genetic and non-genetic testing along with any surplus PK or ADA samples will go to the AstraZeneca Biobank in Gothenburg.

10.8 [redacted]

[redacted] [redacted]

[redacted]	[redacted]	[redacted]	[redacted]	[redacted]
[redacted]	[redacted]	[redacted]	[redacted]	[redacted]
[redacted]	[redacted]	[redacted]	[redacted]	[redacted]
[redacted]	[redacted]	[redacted]	[redacted]	[redacted]
[redacted]	[redacted]	[redacted]	[redacted]	[redacted]
[redacted]	[redacted]	[redacted]	[redacted]	[redacted]
[redacted]	[redacted]	[redacted]	[redacted]	[redacted]
[redacted]	[redacted]	[redacted]	[redacted]	[redacted]
[redacted]	[redacted]	[redacted]	[redacted]	[redacted]
[redacted]	[redacted]	[redacted]	[redacted]	[redacted]
[redacted]	[redacted]	[redacted]	[redacted]	[redacted]
[redacted]	[redacted]	[redacted]	[redacted]	[redacted]
[redacted]	[redacted]	[redacted]	[redacted]	[redacted]
[redacted]	[redacted]	[redacted]	[redacted]	[redacted]
[redacted]	[redacted]	[redacted]	[redacted]	[redacted]
[redacted]	[redacted]	[redacted]	[redacted]	[redacted]
[redacted]	[redacted]	[redacted]	[redacted]	[redacted]
[redacted]	[redacted]	[redacted]	[redacted]	[redacted]
[redacted]	[redacted]	[redacted]	[redacted]	[redacted]
[redacted]	[redacted]	[redacted]	[redacted]	[redacted]

Table 14 [redacted]

[redacted]	[redacted]	[redacted]	[redacted]	[redacted]
[redacted]	[redacted]	[redacted]	[redacted]	[redacted]
[redacted]	[redacted]	[redacted]	[redacted]	[redacted]
[redacted]	[redacted]	[redacted]	[redacted]	[redacted]
[redacted]	[redacted]	[redacted]	[redacted]	[redacted]

[redacted]

SIGNATURE PAGE

This is a representation of an electronic record that was signed electronically and this page is the manifestation of the electronic signature

**Document Name:** d5670C00022-amendment-6		
**Document Title:**	d5670C00022-amendment-6	
**Document ID:**	Doc ID-004264174	
**Version Label:**	1.0 CURRENT LATEST APPROVED	
**Server Date (dd-MMM-yyyy HH:mm 'UTC'Z)**	**Signed by**	**Meaning of Signature**
[redacted]	[redacted]	[redacted]
[redacted]	[redacted]	[redacted]
[redacted]	[redacted]	[redacted]
[redacted]	[redacted]	[redacted]
[redacted]	[redacted]	[redacted]

Notes: (1) Document details as stored in ANGEL, an AstraZeneca document management system.

Statistical Analysis Plan

An Exploratory Phase 2, Randomised, Double-blind, Placebo-controlled, and Open-label Active Comparator Study to Evaluate the Effect of MEDI0382 on Hepatic Glycogen Metabolism in Overweight and Obese Subjects with Type 2 Diabetes Mellitus

Protocol Number: D5670C00022

TABLE OF CONTENTS

1 INTRODUCTION 6
2 STUDY OVERVIEW 6
2.1 Study Objectives 6
2.1.1 Primary Study Objective(s) 6
2.1.2 Secondary Study Objectives 6
2.1.3 Exploratory Study Objectives 6
2.2 Study Design 7
2.3 Treatment Assignment and Blinding 8
2.4 Sample Size 9
3 STATISTICAL METHODS 9
3.1 General Considerations 9
3.2 Analysis Populations 10
3.3 Study Subjects 11
3.3.1 Subject Disposition and Completion Status 11
3.3.2 Demographics and Baseline Characteristics 11
3.3.3 Study Drug Exposure 11
3.3.4 Concomitant Medications 11
3.4 Efficacy Analyses 12
3.4.1 Primary Efficacy Endpoint(s) and Analyses 12
3.4.1.1 Primary Efficacy Endpoint(s) 12
3.4.1.2 Handling of Dropouts and Missing Data 12
3.4.1.3 Primary Efficacy Analysis 12
3.4.2 Secondary Efficacy Endpoint(s) and Analyses 13
3.4.2.1 Secondary Efficacy Endpoint(s) 13
3.4.2.2 Secondary Efficacy Analyses 13
3.4.3 Exploratory Efficacy Endpoint(s) and Analyses 14
3.4.3.1 Exploratory Efficacy Endpoint(s) 14
3.4.3.2 Exploratory Efficacy Analyses 14
3.5 Pharmacodynamic Analyses 14
3.5.1 Exploratory Pharmacodynamic Endpoint(s) and Analyses 14
3.5.1.1 Exploratory Pharmacodynamic Endpoint(s) 14
3.5.1.2 Exploratory Pharmacodynamic Analysis 15
3.6 Safety Analyses 15
3.6.1 Adverse Events and Serious Adverse Events 15
3.6.2 Deaths and Treatment Discontinuations due to Adverse Events 16
3.6.3 Clinical Laboratory Evaluation 16
3.6.4 Other Safety Evaluations 16

3.6.4.1 Vital Signs 16
3.6.4.2 Electrocardiogram 16
3.7 Immunogenicity 17
3.8 Pharmacokinetics 17
3.9 Protocol Deviations 18
4 INTERIM ANALYSIS 18
5 REFERENCES 18
6 VERSION HISTORY 18

LIST OF TABLES

Table 3.2-1 Analysis Populations 11

List of Abbreviations

Abbreviation or Specialized Term	Definition
AE	Adverse event
ANCOVA	Analysis of covariance
ATC	Anatomical Therapeutic Chemical
AUC	Area under the concentration-time curve
BMI	Body mass index
C2	Carbon atom number 2 in glucose molecule (used to assess ^{2}H enrichment)
C6	Carbon atom number 6 in glucose molecule (used to assess ^{2}H enrichment)
CI	Confidence interval
C_{trough}	Trough plasma concentration, measured at the end of a dosing interval, taken directly before next administration
eCRF	Electronic Case Report Form
eGFR	Estimated glomerular filtration rate
ECG	Electrocardiogram
GLP-1	glucagon-like peptide-1
^{2}H	deuterium
HbA1c	Glycated hemoglobin
IM	Immunogenicity
ITT	Intent-to-treat
IV	intravenous
IWRS	Interactive web response system
MedDRA	Medical dictionary for regulatory activities
MMTT	Mixed-meal tolerance test
MRI	Magnetic resonance imaging
MRS	Magnetic resonance spectroscopy
P	Probability
PD	Pharmacodynamic(s)
PK	Pharmacokinetic(s)
PR	ECG PR interval
PT	MedDRA preferred term
QRS	ECG QRS interval
QT	ECG QT interval
QTcF	ECG QT interval corrected for heart rate using Fridericia's formula
RR	ECG RR interval
SAE	Serious adverse event
SAP	Statistical analysis plan
SPP	Statistical Programming Plan

Abbreviation or Specialized Term	Definition
SC	Subcutaneous
SID	Subject ID
SOC	MedDRA system organ class
T	Time
T2DM	Type 2 Diabetes Mellitus
ULN	upper limit of normal
WHO-DD	WHO Drug Dictionary enhanced

1 INTRODUCTION

This document describes the statistical analysis methodology for protocol D5670C00022 (Amendment 6, 15Jul2020), a Phase 2 study to evaluate the effect of MEDI0382 on hepatic glycogen levels post prandially versus placebo in Part A and to compare the effect of MEDI0382 on hepatic glycogen levels versus placebo and liraglutide in Part B in overweight or obese subjects with T2DM. This document details the statistical summaries relating to each study objective and describes the general conventions and definitions that will be used. In addition, a set of table templates and specifications will be included in a statistical programming plan (SPP) to complement this document.

2 STUDY OVERVIEW

2.1 Study Objectives

2.1.1 Primary Study Objective(s)

- To assess the effect of MEDI0382 on hepatic glycogen levels post prandially versus placebo after 28 days (Part A) and 35 days (Part B) of treatment

2.1.2 Secondary Study Objectives

- To assess the effect of MEDI0382 on hepatic glycogen levels post prandially versus liraglutide after 35 days of treatment (Part B only)
- To assess the effect of MEDI0382 on hepatic fat fraction versus placebo after 35 days of treatment (Part B only)
- To evaluate the safety and tolerability of MEDI0382 [redacted]
- To characterize the immunogenicity profile of MEDI0382 [redacted]

2.1.3 Exploratory Study Objectives

- [redacted]

[redacted]

[redacted]

- [redacted]

[redacted]

[redacted]

[redacted]

[redacted]s
[redacted]

[redacted]

2.2 Study Design

This exploratory Phase 2 study has 2 parts (Part A and Part B).

Part A is a randomized, double-blind, placebo-controlled sub-study to evaluate the effect of MEDI0382 ([redacted] administered once daily SC for 28 days on hepatic glycogen metabolism in overweight or obese subjects with T2DM. Part A is planned to randomize up to 20 subjects. Subjects will be consented, screened for suitability, and randomized within 60 days if eligible. Subjects from Part A will not be re-enrolled in Part B.

Part B is contingent on the results from Part A such that the combined sample size of two parts will ensure at least 80% power to detect the treatment effect observed from Part A using the standard deviation information also observed from Part A. If the minimal required sample size for Part B is less than or equal to 10 subjects per arm, Part B will be conducted.

Part B is an exploratory Phase 2 randomized, double-blind, placebo-controlled and open label active comparator sub-study to evaluate the effect of MEDI0382 on hepatic glycogen metabolism in overweight and obese subjects with T2DM subjects with the exact sample size determined by the results from Part A. Subjects in Part B will be randomized to receive double-blind MEDI0382 ([redacted] or placebo or open label liraglutide (titrated from 0.6 to 1.8 mg) once daily SC for 35 days.

Part A:

The study will involve measurement of hepatic glycogen content using a carbon (C)-13 MRS based technique before and after 28 days of treatment. Subjects will undergo a 5-day washout

period where metformin therapy will be suspended at the beginning of the study starting from Day -4 (metformin dosing to resume on Day 2) and the end of the study starting from Day 25 (metformin dosing to resume on Day 30). Across the course of the study (up to 126 days in total including screening) subjects will have a total of 6 study visits, 6 nights of inpatient stay and will undergo a total of 10 MRS scans alongside additional assessments and blood sampling.

Part B:

In Part B a comparison will be made between MEDI0382, [redacted] placebo and liraglutide once daily titrated from 0.6 to 1.8 mg in 7-day intervals. The treatment duration will be 35 days in each treatment group, with subjects randomized to liraglutide spending 21 days at 1.8 mg, in contrast subjects randomized to MEDI0382 will spend 14 days at top dose of 300 µg.

The procedures for subjects who go through to the completion of the visit on Day 8, are identical to Part A; aside from the MRS scanning and related activities that will occur at baseline prior to treatment, then at T = 0, 5, 14 and 24 hours and at the end of the 35 day treatment period as detailed in the schedule of events. The duration of MRS scans will be approximately 40 minutes with the exception of the baseline and end of treatment scans used for liver fat evaluation which will be prolonged and up to 1 hour 45 minutes in duration.

2.3 Treatment Assignment and Blinding

An IWRS will be used for randomization to a treatment group and assignment of investigational product kit numbers. A subject is considered randomized into the study when the IWRS provides the assignment of the subject's investigational product kit number.

Eligible subjects will be randomized at a 1:1 ratio following screening to receive either blinded MEDI0382 SC or placebo SC (Part A) or randomized at a 1:1:1 ratio following screening to receive either blinded MEDI0382 SC or placebo SC or open-label liraglutide SC (Part B). The IWRS will assign a unique randomization code and treatment group to the subject at the time of randomization. Subjects who withdraw from the study may be replaced, if deemed necessary by the medical monitor, to ensure that safety data are collected on a sufficient number of subjects.

In Part A only, this is a double-blind study in which MEDI0382 and placebo [redacted] Neither the subject nor any of the investigator or sponsor staff who are involved in the treatment or clinical evaluation of subjects will be aware of treatment received.

[redacted] To maintain the blind in Part A of the study, investigational product (MEDI0382 and placebo) prefilled syringes will be handled by an unblinded investigational product manager or unblinded study personnel who will not be involved in the treatment or clinical evaluation of subjects. An unblinded site monitor will perform investigational product accountability, and this will be a different person from the blinded site monitor who will oversee other aspects of the study at the clinical site.

[redacted]. Each subject will know the volume of investigational product they have been instructed to select and administer, the subject and site staff are blinded with respect to whether they are receiving MEDI0382 or placebo. Liraglutide used in Part B will be open-label.

The site will maintain a written plan detailing which staff members are blinded/unblinded and the process of investigational product administration used to maintain the blind.

2.4 Sample Size

In Part A, the sample size of 8 completers in each of MEDI0382 and placebo groups provides approximately 80% power to detect a difference of 19% reduction in the baseline glycogen concentration adjusted for liver volume (as measured by MRS at T = 4 hours post standardized liquid morning meal) after 28 days of treatment in the MEDI0382 versus placebo arms. This is under the assumption of baseline glycogen content of 283 µmol/mL and common standard deviation of 41 µmol/mL for both groups and a two-sided significance level at 0.1 for a t-test. (Krssak et al, 2004; Rothman et al, 1991).

In Part B that sample size of 10 completers in each MEDI0382 and placebo groups provides >80% power to detect a 24.2% reduction in fasting glycogen concentration adjusted for liver volume (as measured by MRS at T = 24 hours post standardized liquid morning meal) after 35 days of treatment in the MEDI0382 versus placebo arms. This is under the assumption of a common standard deviation of 17.0 % for both groups, a two-sided significance level at 0.10 for a t-test and is based on the results of Part A.

3 STATISTICAL METHODS

3.1 General Considerations

In the event that there is a delay in data delivery or the full set of results is not expected to be available in timely manner (for example; follow-up of positive ADA results at 3 and 6

months after the follow-up visit) an initial database lock in Part B for the purpose of clinical study analysis and reporting will be performed when the last randomized subject completes the final visit or is discontinued prior to that visit. Final database lock will occur once all results have been received in the database.

In the event that an initial DBL is required, results received after this point may be reported in the clinical study report addendum.

Data will be presented in data listings sorted by study part, treatment, subject number and date collected, where appropriate. Tabular summaries will be presented by study part and treatment. Categorical data will be summarized by study part and treatment with the number and percentage of subjects within each category. In general, continuous variables will be summarized by study part and treatment with descriptive statistics including mean, standard deviation, median, minimum, and maximum. For some variables, the geometric mean and 95% CI may be presented.

All available data will be included in the analyses and missing data will not be imputed except as specified in the calculation of the AUC values. Unless specified otherwise, baseline values will be defined as the last valid assessment prior to the first administration of study medication.

The study objectives will be evaluated with analyses that include data from both Part A and Part B, if Part B is conducted. Analyses will also be performed separately for each study part. In general, the efficacy analyses will be analyzed with the ITT population and all other analyses will be performed with the As-treated population.

All statistical tests will be 2-sided at an alpha = 0.1 significance level unless stated otherwise. There will be no adjustment for multiplicity.

Data analyses will be performed using SAS® version 9.3 or higher (SAS Institute Inc., Cary, NC) in a UNIX environment.

3.2 Analysis Populations

The analysis populations are defined in Table 3.2-1.

Table 3.2-1 Analysis Populations

Population	Description
Intent-to-treat (ITT) population	Subjects who receive any study investigational product will be included in the ITT population and subjects will be analyzed according to their randomized treatment group.
As-treated population	Subjects who receive any study investigational product will be included in the as-treated population and subjects will be analyzed according to the treatment they actually received.
Pharmacokinetic population	The PK population includes all subjects who received at least one dose of investigational product and had at least one PK blood sample taken that is above the lower limit of quantitation.

3.3 Study Subjects

3.3.1 Subject Disposition and Completion Status

A summary of subject eligibility and randomization as well as treatment group received (including summary of subjects randomized but not treated) will be provided by study part. In addition, disposition of subjects throughout the study with respect to completion of treatment and follow-up will be provided.

3.3.2 Demographics and Baseline Characteristics

Demographic information and baseline characteristics will be summarized by study part and by treatment. Demographic information will include: gender, age (years), ethnicity, race, weight (kg), height (cm), and body mass index (BMI) (kg/m^2). A summary of baseline characteristics may include but not limited to eGFR, baseline medication use, HbA1c, glucose, blood pressure, and pulse rate.

3.3.3 Study Drug Exposure

The number of doses and total dose received will be summarized by study part for each investigational product (MEDI0382 or placebo or liraglutide) by descriptive statistics and frequencies.

3.3.4 Concomitant Medications

Concomitant medications will be coded using the current WHO Drug Dictionary enhanced (WHO-DD). The number and percentage of subjects who took concomitant medications for the highest anatomical therapeutic chemical (ATC) class and preferred term will be summarized by study part and treatment. The summary of concomitant medications will include all concomitant medications taken on or after the date of first dose of investigational

product or any concomitant medication started prior to first dose of investigational product that continued beyond the date of first dose of investigational product.

3.4 Efficacy Analyses

3.4.1 Primary Efficacy Endpoint(s) and Analyses

3.4.1.1 Primary Efficacy Endpoint(s)

Change in hepatic glycogen concentration adjusted for liver volume as measured by MRS at T = 4 hours post standardized morning meal from baseline (Day -1) to the end of 28 days of treatment (Part A only)

Percentage change in fasting glycogen concentration adjusted for liver volume as measured by MRS at T = 24 hours post standardized morning meal from baseline (Day 1) to the end of 35 days of treatment (Part B only)

3.4.1.2 Handling of Dropouts and Missing Data

Since there is only one post-baseline measurement MRS, subjects who do not have a valid baseline evaluation or a valid Day 28 (Part A) and Day 35 (Part B) MRS evaluation at 4 hours (Part A) and 24 hours (Part B) post standardized morning meal will not contribute to the analysis.

3.4.1.3 Primary Efficacy Analysis

The primary efficacy analysis will be performed using the ITT population.

The primary endpoint, change in hepatic glycogen concentration (measured by MRS, adjusted for liver volume) at T = 4 hours post standardized morning meal from baseline (Day -1) to the end of 28 days of treatment will be analyzed using an analysis of covariance (ANCOVA) model with baseline covariate and treatment group. Pairwise comparisons between MEDI0382 and placebo will be performed within this analysis (Part A only).

The primary endpoint, percentage change in fasting hepatic glycogen concentration (adjusted for liver volume) as measured by MRS at T=24 hours post standardized morning meal from baseline (Day 1) to the end of 35 days of treatment (Day 36), will be compared between the MEDI0382 and placebo groups using an analysis of covariance (ANCOVA) model adjusting for baseline value and treatment group (Part B only).

3.4.2 Secondary Efficacy Endpoint(s) and Analyses

3.4.2.1 Secondary Efficacy Endpoint(s)

- Percentage change in fasting hepatic glycogen concentration adjusted for liver volume as measured by MRS at T = 24 hours post standardized morning meal from baseline (Day -1) to the end of 28 days of treatment (versus placebo, Part A only)
- Percentage change in fasting hepatic glycogen concentration adjusted for liver volume as measured by MRS at T = 24 hours post standardized morning meal from baseline (Day 1) to the end of 35 days of treatment (versus liraglutide, Part B only)
- Change in hepatic fat fraction from baseline as measured by magnetic resonance imaging (Day -1) to the end of 28 days of treatment (Part A only)
- Change in hepatic fat fraction from baseline as measured by magnetic resonance imaging (Day 1) to the end of 35 days of treatment (Part B only)
- Measures of safety and tolerability (vital signs, ECGs, laboratory test results, AEs)
- Development of ADA and titre (if confirmed positive)

3.4.2.2 Secondary Efficacy Analyses

The secondary efficacy analyses will be performed using the ITT population.

The percentage change in fasting hepatic glycogen concentration (measured by MRS, adjusted for liver volume) at T = 24 hours post standardized morning meal from baseline (Day 1) to the end of 35 days of treatment, will be compared between MEDI0382 and liraglutide groups only in Part B of the study. This will be done using the ANCOVA model adjusting for baseline value and treatment group. A similar analysis will be applied to measure change in hepatic fat fraction from baseline (Day -1 or Day 1) to the end of 35 days of treatment (Day 35 or 36) versus placebo (Part B only).

The percentage change in fasting glycogen concentration (measured by MRS, adjusted for liver volume) at T = 24 hours post standardized morning meal from baseline (Day -1) to the end of 28 days of treatment, will be compared between MEDI0382 and placebo groups only in Part A of the study. This will be done using the ANCOVA model adjusting for baseline value and treatment group. A similar analysis will be applied to measure change in hepatic fat fraction from baseline (Day -1) to the end of 28 days of treatment versus placebo (Part A only).

3.4.3 Exploratory Efficacy Endpoint(s) and Analyses

3.4.3.1 Exploratory Efficacy Endpoint(s)

- [redacted]
- [redacted]
- [redacted]
- [redacted]
- [redacted]
- [redacted]
- [redacted]

3.4.3.2 Exploratory Efficacy Analyses

The exploratory efficacy analyses with the ITT population will be performed using an analysis of covariance (ANCOVA) model adjusting for baseline value and treatment group. The analyses will be performed as well as analyzed separately for each part.

3.5 Pharmacodynamic Analyses

3.5.1 Exploratory Pharmacodynamic Endpoint(s) and Analyses

3.5.1.1 Exploratory Pharmacodynamic Endpoint(s)

- [redacted]

- [redacted]

[redacted]

3.5.1.2 Exploratory Pharmacodynamic Analysis

The exploratory efficacy analyses will be performed using an analysis of covariance (ANCOVA) model adjusting for baseline value and treatment group. The analyses will be performed only separately for each part.

3.6 Safety Analyses

3.6.1 Adverse Events and Serious Adverse Events

Adverse events will be coded with MedDRA version 21.0 or higher. Analysis of adverse events will include the type, incidence, severity and relationship to study investigational product summarized by MedDRA System Organ Class (SOC) and Preferred Term (PT) by study part treatment group as well as for combined study parts. The AEs summaries will include only treatment-emergent AEs, ie, those occurring after initial administration of investigational product. Subjects will be counted once for specific PT or MedDRA SOC when calculating incidence rates. If the same AE Preferred Term occurs multiple times within a subject, the highest severity and level of relationship observed will be reported. Non-treatment-emergent AEs/serious adverse events (SAEs) will be presented in the listings.

3.6.2 Deaths and Treatment Discontinuations due to Adverse Events

A listing of any deaths will be provided which will include the MedDRA system organ class and preferred term. AEs resulting in permanent discontinuation from study drug will be summarized by study part and treatment. The summary will include results summarized overall and by MedDRA System Organ Class and Preferred Term.

3.6.3 Clinical Laboratory Evaluation

Hematology, serum chemistry, and urinalysis laboratory evaluations will be performed during the study. The hematology and serum chemistry (including calcitonin, lipase, and amylase) parameters as well as their changes from baseline will be summarized with descriptive statistics (number of subjects, mean, and standard deviation, median, minimum and maximum) by study part and treatment group. Other laboratory parameters collected will be summarized in a similar manner. The hematology and serum chemistry results will also be classified as low, normal, or high. The urinalysis results will be classified as normal or abnormal. The shift from baseline hematology, serum chemistry, and urinalysis results will be summarized by study part and treatment at each evaluation time.

3.6.4 Other Safety Evaluations

3.6.4.1 Vital Signs

Vital signs including pulse rate (beats/min), systolic and diastolic blood pressure (mmHg), temperature (oC), and respiratory rate (breaths/min), as well as the change from baseline for each of those parameters, will be descriptively summarized study part and treatment.

3.6.4.2 Electrocardiogram

Electrocardiogram parameters will be assessed with a single ECG at each evaluation (Screening, Day 1, and Day 26) using a digitally recorded standard 12-lead electrocardiograph. The ECG parameters: Heart rate, RR, PR, QRS and QT intervals as well as derived parameter QT corrected interval QTcF (Fridericia's formula $\frac{QT}{\sqrt[3]{RR}}$) will be summarized. The results at Day 26 as well as the change from baseline (Day 1) will be descriptively summarized by study part and treatment. For the qualitative variable ECG interpretation, the ECG Interpretation results will be summarized with frequencies and percentages at each evaluation.

3.7 Immunogenicity

All subjects in the safety analysis set with reported anti-drug antibody (ADA) results (ADA positive or ADA negative, titer, cross-reactivity to GLP1 (positive or negative), cross-reactivity to glucagon (positive or negative) will be shown in the data listing.

ADA status (positive vs. negative) will be summarized by treatment group according to the following categories:

ADA prevalence: subjects who are ADA positive at any visit (including baseline)

Subjects who are ADA positive at baseline only

Subjects who are ADA positive at baseline and positive post baseline

Subjects who are ADA positive post-baseline only (treatment-induced ADA)

Subjects who are persistently positive; persistently positive is defined as at least 2 post-baseline ADA positive measurements (with >=16 weeks apart) or an ADA positive result at the last available assessment

Proportion of subjects who are transiently positive; transiently positive is defined as at least one post-baseline ADA positive measurement and not fulfilling the conditions for persistently positive.

Proportion of subjects who are treatment-boosted ADA; treatment-boosted ADA is defined as baseline ADA titer that was boosted to a 4-fold or higher level following drug administration

ADA incidence (treatment-emergent ADA), defined as the sum of treatment-induced ADA (post-baseline positive only) and treatment-boosted ADA

Similar summary will be performed for ADA cross-reactivity to GLP-1 and/or glucagon (as data allows and if applicable).

The association of ADA status with PK, PD endpoints, efficacy, and safety may be evaluated if data allows and if applicable.

3.8 Pharmacokinetics

The analysis of the pharmacokinetic data is described below. However, the results of these analyses will be provided in the pharmacokinetic report that is prepared by the

pharmacokineticist; therefore no tables, figures or listings of pharmacokinetic data will be specified in the SPP.

Plasma C_{trough} concentrations of MEDI0382 at steady state (Part A Day 15 and Day 28; Part B Day 35) will be summarized with descriptive statistics.

3.9 Protocol Deviations

A summary of important protocol deviations (IPD) will be prepared by deviation category as well as overall. A listing of the important protocol deviations will be also provided.

4 INTERIM ANALYSIS

An interim analysis (ie, Part A analysis) was performed after Part A subjects have completed the study.

5 REFERENCES

None

6 VERSION HISTORY

Version	Date	Summary of Changes	Reason for Change
1.0	14May2018	Initial document	Initial document
2.0	04Jun2018	- Updated Protocol D5670C00022 (Amendment 3, 8May2018).<br>- Add subsection 3.9 – Protocol Deviations A summary of important protocol deviation (IPD) will be prepared by deviation category as well as overall, A listing of the important protocol deviations will be also provided.	Incorporated Team Review Comments
3.0	31May2021	- Updated Protocol D5670C00022 (Amendment 6, 15July2020).<br>- Amend for potential 2 DBLs of Part B	Incorporated Team Review Comments

SIGNATURE PAGE

This is a representation of an electronic record that was signed electronically and this page is the manifestation of the electronic signature

**Document Name:** d5670c00022-sap-ed-3		
**Document Title:**	Statistical Analysis Plan Edition 3	
**Document ID:**	Doc ID-004586895	
**Version Label:**	1.0 CURRENT LATEST APPROVED	
**Server Date (dd-MMM-yyyy HH:mm 'UTC'Z)**	**Signed by**	**Meaning of Signature**
04-Jun-2021 14:02 UTC	[redacted]	[redacted]
[redacted]	[redacted]	[redacted]

Notes: (1) Document details as stored in ANGEL, an AstraZeneca document management system.
