# Supplementary material for: Cotadutide promotes glycogenolysis in people with overweight or obesity diagnosed with type 2 diabetes
Source: Nat Metab. 2023 Dec 8;5(12):2086–93. doi: 10.1038/s42255-023-00938-0 (PMC10730390; doi:10.1038/s42255-023-00938-0)
Supplement: Supplementary file 2 — Reporting Summary [file 42255_2023_938_MOESM2_ESM.pdf]

nature portfolio

Corresponding author(s): Victoria ER Parker
Last updated by author(s): Oct 4, 2023

Reporting Summary

Nature Portfolio wishes to improve the reproducibility of the work that we publish. This form provides structure for consistency and transparency in reporting. For further information on Nature Portfolio policies, see our Editorial Policies and the Editorial Policy Checklist.

Statistics

For all statistical analyses, confirm that the following items are present in the figure legend, table legend, main text, or Methods section.

n/a	Confirmed	
☐	☒	The exact sample size (*n*) for each experimental group/condition, given as a discrete number and unit of measurement
☐	☒	A statement on whether measurements were taken from distinct samples or whether the same sample was measured repeatedly
☐	☒	The statistical test(s) used AND whether they are one- or two-sided<br>*Only common tests should be described solely by name; describe more complex techniques in the Methods section.*
☐	☒	A description of all covariates tested
☐	☒	A description of any assumptions or corrections, such as tests of normality and adjustment for multiple comparisons
☐	☒	A full description of the statistical parameters including central tendency (e.g. means) or other basic estimates (e.g. regression coefficient) AND variation (e.g. standard deviation) or associated estimates of uncertainty (e.g. confidence intervals)
☐	☒	For null hypothesis testing, the test statistic (e.g. *F*, *t*, *r*) with confidence intervals, effect sizes, degrees of freedom and *P* value noted<br>*Give P values as exact values whenever suitable.*
☒	☐	For Bayesian analysis, information on the choice of priors and Markov chain Monte Carlo settings
☒	☐	For hierarchical and complex designs, identification of the appropriate level for tests and full reporting of outcomes
☒	☐	Estimates of effect sizes (e.g. Cohen's *d*, Pearson's *r*), indicating how they were calculated

Our web collection on statistics for biologists contains articles on many of the points above.

Software and code

Policy information about availability of computer code

Data collection	*Provide a description of all commercial, open source and custom code used to collect the data in this study, specifying the version used OR state that no software was used.*
Data analysis	SAS® version 9.3 or higher (SAS Institute Inc., Cary, NC) in a UNIX environment; MATLAB (MATLAB 2014b, The MathWorks, Inc., Natick, MA, USA).

For manuscripts utilizing custom algorithms or software that are central to the research but not yet described in published literature, software must be made available to editors and reviewers. We strongly encourage code deposition in a community repository (e.g. GitHub). See the Nature Portfolio guidelines for submitting code & software for further information.

Data

Policy information about availability of data

All manuscripts must include a data availability statement. This statement should provide the following information, where applicable:

- Accession codes, unique identifiers, or web links for publicly available datasets
- A description of any restrictions on data availability
- For clinical datasets or third party data, please ensure that the statement adheres to our policy

Data underlying the findings described in this article may be obtained in accordance with AstraZeneca’s data sharing policy described at: https://

astrazenecagrouptrials.pharmacm.com/ST/Submission/Disclosure. Information regarding where to submit a request to access data, conditions of access, time frame for response to requests and details of any restrictions imposed on data use via data use agreements are available here.

Research involving human participants, their data, or biological material

Policy information about studies with human participants or human data. See also policy information about sex, gender (identity/presentation), and sexual orientation and race, ethnicity and racism.

Reporting on sex and gender	This study included both sexes and gender data were not collected. Owing to the small sample size, sex was not considered in the study design and data were not disaggregated for sex.
Reporting on race, ethnicity, or other socially relevant groupings	Owing to the small sample size, race, ethnicity or any other socially relevant groupings were not considered in the study design. All participants were white, influenced primarily by the geographical location of study sites in Northern Europe.
Population characteristics	Both parts of the study included men and women aged ≥18 years, with a BMI of 27–40 kg/m2 and type 2 diabetes mellitus (HbA1c ≤ 8.0% (64 mmol/mol)) receiving metformin monotherapy. Part A included 21 patients (13 males, 8 females) with a mean age of 67.3 years old, mean BMI of 32.1 kg/m2 and mean HbA1c of 6.4%. Part B included 30 patients (21 males, 9 females) with a mean age of 63.7 years old, mean BMI of 30.9 kg/m2 and mean HbA1c of 6.9%. Sex was determined on the basis of self-report.
Recruitment	Independent Ethics Committees at the study sites approved all advertising used to recruit patients for the study. Patients were enrolled by study site investigators and randomized after assessment of study eligibility was complete. Investigators ensured each subject was given full and adequate oral and written information about the nature, purpose, possible risk, and benefit of the study. They ensured that each potential participant was given the opportunity to ask questions and allowed time to consider the information provided. No self-selection bias was anticipated nor identified. Eligible patients for both parts of the study were men and women aged ≥18 years, with a BMI of 27–40 kg/m2 and type 2 diabetes mellitus (HbA1c ≤8.0% (64 mmol/mol)) receiving metformin monotherapy with no significant dose adjustment (increase or decrease ≥500 mg/day) 3 months prior to enrolment. Patients had to have AST and ALT levels <3 times the upper limit of normal (ULN) and bilirubin levels <2 times ULN, as well as an estimated glomerular filtration rate ≥30 ml/min/1.73 m2 (Modification of Diet in Renal Disease calculation). Prior use of GLP-1 receptor analogue-based therapy within 30 days of enrolment and daily insulin within 90 days of enrolment was not allowed. Other key exclusion criteria included recurrent hypoglycaemia (blood glucose <3.0 mmol/l (<54 mg/dl) at least twice within 6 months prior to enrolment), clinically significant liver disease other than NAFLD or NASH and a history of acute or chronic pancreatitis, type 1 diabetes mellitus or diabetic ketoacidosis and history of heavy alcohol use. Participants required a negative alcohol test at screening and randomization.
Ethics oversight	The study was conducted in accordance with the principles of the Declaration of Helsinki, the International Council for Harmonization Guidance for Good Clinical Practice and was approved by Independent Ethics Committees at Maastricht University Medical Centre, Maastricht, Netherlands and Linköping University, Linköping, Sweden. National regulatory authority in each country were notified of and approved the study. Written informed consent forms and any other written information/materials to be provided to participants were approved by the Independent Ethics Committees. Written informed consent from all participants was required for inclusion into the trial. The study is registered at clinicaltrials.gov (NCT03555994).

Note that full information on the approval of the study protocol must also be provided in the manuscript.

Field-specific reporting

Please select the one below that is the best fit for your research. If you are not sure, read the appropriate sections before making your selection.

☒ Life sciences ☐ Behavioural & social sciences ☐ Ecological, evolutionary & environmental sciences

For a reference copy of the document with all sections, see nature.com/documents/nr-reporting-summary-flat.pdf

Life sciences study design

All studies must disclose on these points even when the disclosure is negative.

Sample size	In Part A, the sample size of eight in each study group would provide > 80% power to detect 19% difference between treatment groups for the primary endpoint, with a two-sided significance level of 0.1 (corresponding to 90% CI, selected to minimize sample size in this exploratory study), assuming mean baseline glycogen concentration of 283 µmol/ml (SD, 41 µmol/ml) for both groups. In Part B, the sample size of 10 in the cotadutide and placebo groups would provide > 80% power to detect 24.3% difference between treatment groups for the primary endpoint, with a two-sided significance level of 0.1, assuming a SD of 17% for both groups. Given the small sample size and small number of sites, sex and gender sub-analyses were not performed as they would not be sufficiently powered to generate meaningful results. In view of this sex was not considered in the study design and gender data was not collected.
Data exclusions	All available data were included in the analyses and missing data was not be imputed. Safety was assessed in all randomized patients who received at least one dose of study treatment. Secondary and exploratory analyses were performed in all randomized patients who received treatment and for whom data were available at baseline and end of treatment follow-up visit.
Replication	No findings were replicated in this phase 2a clinical trial.

Randomization	In Part A, randomization was 1:1 to either subcutaneous once-daily cotadutide titrated 100–300 µg or matching placebo. In Part B, randomization was 1:1:1 to either subcutaneous once-daily cotadutide titrated 50–300 µg , subcutaneous once-daily liraglutide titrated 0.6–1.8 mg, or placebo. Assignment to treatment groups and investigation product kit numbers was determined by a computer-generated randomized sequence (Covance, Inc.), with use of interactive web response systems and interactive voice-response systems.
Blinding	Part A was double-blinded, with both investigators, patients and sponsor staff who are involved in the treatment or clinical evaluation of patients unaware of treatment allocation. An unblinded site monitor, whom was not involved in treatment or clinical evaluation of patients, performed investigational product accountability. Part B was a part-blinded, randomized, active-comparator study with investigators and patients unaware of allocation to either placebo or cotadutide, but liraglutide was open-label. The cotadutide and placebo multidose pens were indistinguishable.

Reporting for specific materials, systems and methods

We require information from authors about some types of materials, experimental systems and methods used in many studies. Here, indicate whether each material, system or method listed is relevant to your study. If you are not sure if a list item applies to your research, read the appropriate section before selecting a response.

Materials & experimental systems

n/a	Involved in the study	
☒	☐	Antibodies
☒	☐	Eukaryotic cell lines
☒	☐	Palaeontology and archaeology
☒	☐	Animals and other organisms
☐	☒	Clinical data
☒	☐	Dual use research of concern
☒	☐	Plants

Methods

n/a	Involved in the study	
☒	☐	ChIP-seq
☒	☐	Flow cytometry
☒	☐	MRI-based neuroimaging

Clinical data

Policy information about clinical studies

All manuscripts should comply with the ICMJE guidelines for publication of clinical research and a completed CONSORT checklist must be included with all submissions.

Clinical trial registration	ClinicalTrials.gov: NCT03555994
Study protocol	D5670C00022
Data collection	The study took place between May 2018 and April 2021. Data were collected at the study sites (Maastricht University Medical Centre, Maastricht, Netherlands and Linköping University, Linköping, Sweden)
Outcomes	The overall primary objective was to evaluate the effect of cotadutide on hepatic glycogen levels compared with placebo, as evidence of glucagon receptor engagement (Part A primary endpoint: change from baseline to day 28 in postprandial hepatic glycogen with cotadutide versus placebo; Part B primary endpoint:Part B was change from baseline to day 35 of treatment in fasting hepatic glycogen adjusted for liver volume). Secondary objectives were to evaluate the effect of cotadutide on hepatic glycogen levels adjusted for liver volume and hepatic fat fraction compared with liraglutide (Part B) and to evaluate the safety and tolerability of cotadutide titrated up to a dose level of 300 µg.
